# Pupillometry and autonomic nervous system responses to cognitive load and false feedback: an unsupervised machine learning approach

**DOI:** 10.3389/fnins.2024.1445697

**Published:** 2024-08-30

**Authors:** Evgeniia I. Alshanskaia, Galina V. Portnova, Krystsina Liaukovich, Olga V. Martynova

**Affiliations:** ^1^Faculty of Social Sciences, School of Psychology, National Research University Higher School of Economics, Moscow, Russia; ^2^Institute of Higher Nervous Activity and Neurophysiology of the Russian Academy of Sciences, Moscow, Russia; ^3^Centre for Cognition and Decision Making, Institute for Cognitive Neuroscience, National Research University Higher School of Economics, Moscow, Russia

**Keywords:** pupillometry, cognitive load, machine learning, heart rate variability, oculomotor parameters, k-means clustering

## Abstract

**Objectives:**

Pupil dilation is controlled both by sympathetic and parasympathetic nervous system branches. We hypothesized that the dynamic of pupil size changes under cognitive load with additional false feedback can predict individual behavior along with heart rate variability (HRV) patterns and eye movements reflecting specific adaptability to cognitive stress. To test this, we employed an unsupervised machine learning approach to recognize groups of individuals distinguished by pupil dilation dynamics and then compared their autonomic nervous system (ANS) responses along with time, performance, and self-esteem indicators in cognitive tasks.

**Methods:**

Cohort of 70 participants were exposed to tasks with increasing cognitive load and deception, with measurements of pupillary dynamics, HRV, eye movements, and cognitive performance and behavioral data. Utilizing machine learning k-means clustering algorithm, pupillometry data were segmented to distinct responses to increasing cognitive load and deceit. Further analysis compared clusters, focusing on how physiological (HRV, eye movements) and cognitive metrics (time, mistakes, self-esteem) varied across two clusters of different pupillary response patterns, investigating the relationship between pupil dynamics and autonomic reactions.

**Results:**

Cluster analysis of pupillometry data identified two distinct groups with statistically significant varying physiological and behavioral responses. Cluster 0 showed elevated HRV, alongside larger initial pupil sizes. Cluster 1 participants presented lower HRV but demonstrated increased and pronounced oculomotor activity. Behavioral differences included reporting more errors and lower self-esteem in Cluster 0, and faster response times with more precise reactions to deception demonstrated by Cluster 1. Lifestyle variations such as smoking habits and differences in Epworth Sleepiness Scale scores were significant between the clusters.

**Conclusion:**

The differentiation in pupillary dynamics and related metrics between the clusters underlines the complex interplay between autonomic regulation, cognitive load, and behavioral responses to cognitive load and deceptive feedback. These findings underscore the potential of pupillometry combined with machine learning in identifying individual differences in stress resilience and cognitive performance. Our research on pupillary dynamics and ANS patterns can lead to the development of remote diagnostic tools for real-time cognitive stress monitoring and performance optimization, applicable in clinical, educational, and occupational settings.

## 1 Introduction

The physiological response to deceptive cues under increasing cognitive load provides critical insights into both neuroscientific understanding and the advancement of neurophysiological and psychological assessment methods. Pupillometry, a non-invasive technique, effectively monitors autonomic nervous system (ANS) responses (Ferencová et al., 2021; Lowenstein and Loewenfeld, 1950; Sirois and Brisson, 2014) in real time and is associated with cognitive load and stress (Rattan et al., 2023; Sabatino DiCriscio et al., 2018; Simonovic et al., 2018; Wilhelm et al., 2014), and it is linked to the locus coeruleus-noradrenaline (LC-NA) system (Bari et al., 2020; Borodovitsyna et al., 2018; Ressler and Nemeroff, 2001), and functions such as memory and attention (Daniels et al., 2012; Viglione et al., 2023) and cognitive effort (Franzen et al., 2022; Marshall, 2007; Ruiz Puentes et al., 2023; Yoo et al., 2021). Furthermore, oculomotor metrics like saccades, fixations, and blinks are essential for understanding cognitive load or integrated with pupillometry to offer insights into cognitive states (Angelidis et al., 2019; Bezrukikh et al., 2018; Calancie et al., 2022; Herten et al., 2017; Kan et al., 2021; Kim et al., 2022; Lipp and Hardwick, 2003; Marquart et al., 2015; Nakano and Kuriyama, 2017; Sciaraffa et al., 2021; Skaramagkas et al., 2023; Zargari Marandi et al., 2018).

Heart Rate Variability (HRV) is another metric used to assess ANS responses to stress and deception. Studies have shown HRV’s correlation with stress (Peabody et al., 2023) and cognitive load. Pupillometry and cardiac metrics were also combined to assess patterns of ANS responses (Hoogerbrugge et al., 2022; Ma et al., 2024; Venkata Sivakumar et al., 2020). Integrating existing research on deception, spanning eye-tracking, blink patterns, saccades behavioral, and skin-conductance (Fang et al., 2021; Fukuda, 2001; Macatee et al., 2017; Proudfoot et al., 2016; Suchotzki and Gamer, 2019; Tomash and Reed, 2015; Wang et al., 2010; Webb et al., 2009), with HRV metrics could offer a comprehensive understanding of ANS responses to deceit.

Previous studies (Ganis, 2003; Kozel et al., 2004; Ströfer et al., 2015) also utilized fMRI to analyze the neural processes associated with misleading and deception. However, the research incorporating Skin Galvanic Response (SGR) or electrodermal activity (EDA) alongside fMRI (Ganis, 2003; Kozel et al., 2004; Ströfer et al., 2015) provides mixed results, despite it being primarily influenced by the sympathetic nervous system (SNS) but also modulated by parasympathetic neurotransmitters (Coon and Low, 2023) and very promising for real-time stress analysis (Setz et al., 2010) with machine learning techniques (Rahma et al., 2022). Additionally, studies investigating breathing with HRV (Meehan and Shaffer, 2024), and under cognitive stress and deception (Grassmann et al., 2016; Kaplan et al., 2023; Kurohara et al., 2001) underscore the necessity for further research to corroborate and extend upon these findings. There is a growing trend in employing unsupervised algorithms to refine the way for personalized diagnostics and deception detection (Celniak et al., 2023; Chang and Chen, 2023; Constâncio et al., 2023; Khalil et al., 2022).

Physiologically, in pupillary function, the SNS uses alpha-1 adrenergic receptors to dilate the pupil via the superior cervical ganglion. The parasympathetic nervous system (PNS) uses M3 muscarinic receptors to constrict the pupil via the Edinger-Westphal nucleus and oculomotor nerve (cranial nerve III) (Ferencová et al., 2021; May et al., 2019). Sympathetic signals contract radial muscles with norepinephrine, while parasympathetic signals contract circular muscles with acetylcholine, demonstrating precise regulation of pupil size. Interestingly, these systems show high coherence of switching in certain reactions. The coherence of pupillometry and HRV parameters, as it was mentioned in previous works (Ma et al., 2024; Macatee et al., 2017; Shi et al., 2022), provides insights warranting further research of SNS-PNS balance and potential therapeutic targets for mental states.

The coherent functioning and imbalances of the ANS branches are investigated to gain a better understanding of their distinct properties in depression, epilepsy, schizophrenia, post-Covid conditions, and healthy volunteers (Asarcikli et al., 2022; Buchholz et al., 2017; McCraty and Zayas, 2014; Sgoifo et al., 2015; Stogios et al., 2021; Udupa et al., 2007). The regulation of the heart rate, breathing, skin conductance, and other vegetative functions, signal intensity in both sympathetic and parasympathetic ganglia is modulated by specific subtypes of nicotinic acetylcholine receptors (nAChRs) such as α3β4, α7, and α4β2 (Halder and Lal, 2021; Ho et al., 2020; Scholze and Huck, 2020; Tizabi et al., 2023), facilitating fast synaptic transmission. Additionally, peripheral signal intensity is regulated by inhibitory alpha-2 adrenergic receptors (Drouin et al., 2017), alongside the balance between sympathetic and parasympathetic inputs. In the heart, this equilibrium is governed by sympathetic beta-1 adrenergic receptors and parasympathetic M2 muscarinic acetylcholine receptors (Bernstein et al., 2011; Harvey and Belevych, 2003). The oculomotor dynamics involving cranial nerves IV (trochlear nerve) and VI (abducens nerve) are part of complex neural circuits (Siegelbaum, 2021). The non-invasive regulation and analysis of ANS responses provide additional insights for understanding individual stress-coupling behavior.

Despite advancements, gaps remain in understanding of the consistency and interplay among physiological measures (Iacono and Ben-Shakhar, 2019; Kozel et al., 2004). This study aims to provide an integrated understanding for leveraging the potential of neurophysiological diagnostics. As the size of the pupil is influenced by both the sympathetic and parasympathetic branches of the nervous system, we theorized that changes in pupil size dynamics under cognitive load, when combined with misleading feedback, can predict individual behavior, as well as ANS reactions that reflect specific adaptation to cognitive stress. In order to examine this, we utilized an unsupervised machine learning method to identify clusters of individuals differentiated by the dynamics of pupil dilation, and then evaluated their ANS responses in relation to reaction time, performance, and self-esteem indicators during cognitive tasks. By utilizing high-resolution pupillometry, oculomotor metrics, HRV, respiratory and EDA along with behavioral parameters, and focusing on reactions to deceptive stimuli under increasing cognitive load, this study contributes to the development of precise pupillometry-based diagnostic tools by investigating the complex interrelation of cognitive and autonomic nervous system responses.

## 2 Materials and methods

### 2.1 Participants

A cohort of 73 individuals, initially recruited between the ages of 18 and 45 years, participated in the study. After removing 3 individuals due to technical issues in their data recordings, 70 subjects (26.17 ± 8.44 years, 25 males and 45 females) were included in the final analysis. Participants were invited via social networks and the faculty’s public website. Uniform assessments, consisting of questionnaires and medical histories, were conducted for all participants following a standardized protocol that ensured confidentiality and data anonymization. All participants signed the informed consent agreement prior to participation, and all the data were anonymized. The study adhered to the ethical statement of the Declaration of Helsinki and the study was approved by the local ethics committee of the institute. To minimize physiological variations that could influence the results, participants were instructed to abstain from consuming caffeine (coffee and tea) and nicotine (smoking) for at least 12 h before the experiment.

### 2.2 Preliminary assessment

The participants underwent a detailed anamnestic review to report their smoking habit and questionnaires. Smoking was assessed using a binary scale, where ‘0’ indicated no smoking during the individual’s last year and ‘1’ indicated the presence of any smoking, vapes, hookah or shisha on a regular basement in the past year. Preliminary assessments included the Beck Depression Inventory (BDI-II) (Beck, 1961; Beck et al., 2011), The Epworth Sleepiness Scale (ESS) (Johns, 1991) to assess the excessive daytime sleepiness, Trait Version (STAI) by Spielberger (Spielberger, 2012; Spielberger and Reheiser, 2009), to differentiate between state and trait anxiety.

### 2.3 Stimuli and procedure

For inducing cognitive load, we employed the cognitive Colour Matching Task (CMT) (Arsalidou et al., 2010). Participants were instructed to view images of colored balloons and compare each with the preceding one to identify color matches. Key 1 of the computer keyboard was designated for matches, and key 2 for mismatches, with a primary emphasis on color rather than position. The task consists of three blocks (Block 1, Block 2, and Block 3), each comprising six escalating difficulty levels (Level 1 to Level 6). Each level includes 17 trials, totaling 102 samples per block (306 samples for 3 blocks). It commences with recognizing a single color change (Level 1) in an image with balloons and escalates up to six color changes (Level 6). Each block took about 7 min, varying with response times. The total experiment lasted up to 24 min per participant, with 30-min breaks between blocks. Participants received immediate feedback after each response. The experiment included deceptive incorrect feedback in Block 2, levels 3–6. After finishing a level, participants assessed their self-esteem on a 5-point scale regarding their task performance, with ratings from 1 (many errors) to 5 (perfect execution). All participant responses were documented and subsequently analyzed in relation to their satisfaction with task completion.

We assessed how participants managed cognitive load by correlating their physiological responses with their correct answers and time they spent. Participants’ rewards included a base amount for participation, supplemented by a performance-based bonus calculated according to the number of correct answers, following the methodology employed in the pilot studies (Portnova et al., 2023; Proskurnina et al., 2023).

### 2.4 Recording and signal processing

Eye-tracking data, include pupillometry, oculomotor and time of response parameters, were acquired with EyeLink Portable Duo SR Research eye-tracker with 2 ms temporal resolution (at 500 Hz sampling rate) (Mississauga, ON, Canada: SR Research Ltd., 2020) in a head-stable mode. Optimizing the frequency resolution to 10 Hz is generally recommended for data analysis and storage (Laeng and Alnaes, 2019; Steinhauer et al., 2004) or 50 Hz downsampling (Van Rij et al., 2019), but other approaches are also applied (Hershman et al., 2023; Mathôt and Vilotijević, 2022). We averaged the pupil size data for each trial exposition lasting a few seconds, so we did not need the sampling rate of 10 Hz. in this study we were not interested in momentary reactions of the pupil, but focused on the more longitudinal coarse-grained dynamics of the reaction to increasing cognitive load throughout the entire experiment, allowing us to understand the reaction pattern. The research demonstrated effects on function processing at frequencies up to 200 Hz for gazes and 50 Hz for pupillometry (Kucewicz et al., 2018). Additionally, studies (Naber et al., 2013; Graff et al., 2019) revealed robust responses at 500 Hz in the visual system and significant temporal processing at 250 Hz in the auditory system, respectively.

We investigated the participants’ pupillometry dynamic patterns in response to increasing difficulty, clustering them to analyze ANS responses. Consequently, we averaged the pupil size over the duration of each trial. The subjects performed a total of 306 trials, resulting in a dataset comprising 306 time-ordered measurements of pupil size. We analyzed these datasets to identify patterns in response to increasing complexity, the number of tasks completed, and the presence of false feedback in Block 2 at difficulty levels 3 to 6. This approach eliminates the necessity for intermediate downsampling from 500 Hz to 10 Hz and demonstrates the application of the k-means clustering method in pupil dynamics analysis.

The process of normalization was carried out by computing the mean pupil size and the standard deviation of the pupil size for each participant over the entire test period. Subsequently, each individual measurement of the participant’s pupil size was adjusted by subtracting the mean pupil size of the participant and then dividing the difference by the standard deviation of the participant’s pupil size. This procedure yielded the normalized pupil size for each participant (Fink et al., 2023).

Stimuli sequence and data for the analysis of behavioral responses were obtained using Experiment Builder 2.3.1 synchronized with electrocardiogram (ECG) and EDA, breathing parameters (RSP) and skin conductance response (SGR). ECG, RSP and SGR/EDA data were continuously recorded to assess autonomic nervous system reactions using a rheograph-polyanalyzer RGPA-6/12 (Medicom-MTD, Taganrog, Russia), with a sampling rate of 250 Hz and with the following filtration ranges: 0.5–75 Hz for ECG, 0.05–2 Hz for SGR/EDA and 0.05–5 Hz for RSP.

ECG measurements utilized sensors strategically placed on the right and left wrists, as well as the right ankle. The QRS complexes in the ECG signal were identified based on their gradient steepness, with R-peaks recognized as local maxima (Brammer, 2020; Makowski et al., 2021). The ECG rough signal was preprocessed with HRV BioPsyKit, known for its artifact detection capabilities (Richer et al., 2021), with fifth-order Butterworth high-pass filter with a cutoff frequency of 0.5 Hz was applied, in combination with a 50 Hz power line to filter additional peaks (Berntson et al., 1990). Following the initial processing with Neurokit2 (Makowski et al., 2021) for the analysis of HRV, the metrics derived from the time-domain, frequency-domain analyses include the following: MeanNN (mean of normal-to-normal RR peaks intervals) reflecting vagal parasympathetic activity, SDNN (standard deviation of normal-to-normal intervals) higher values indicate better autonomic function, RMSSD (root mean square of successive differences, indicating parasympathetic activity), LF (low frequency power), associated with both sympathetic and parasympathetic activity, but higher values may indicate increased sympathetic activity; HF (high frequency power) linked to parasympathetic activity; LF/HF ratio (low frequency/high frequency ratio) indicate the balance between sympathetic and parasympathetic activity and higher values suggest greater sympathetic dominance (Task Force of the European Society of Cardiology the North American Society of Pacing Electrophysiology, 1996; Pham et al., 2021; Shaffer and Ginsberg, 2017).

The SGR/EDA sensors were placed on the distal phalanx of the index and ring fingers of the left hand and the RSP abdominal sensor was placed on the diaphragm area. For the analysis of and respiratory and skin conductance parameters (RSP Amplitude Mean, RSP Phase Duration Expiration, RSP Phase Duration Inspiration, RSP Phase Duration Ratio, RSP Rate Mean and SCR Peaks Amplitude Mean, SCR Peaks Number, respectively) was employed Neurokit2 software.

### 2.5 Machine learning clustering method

The pupil size data were normalized for mean and variance for each participant to eliminate individual differences in pupil size from consideration before clustering. Furthermore, the data preprocessed in Python included a stage dedicated to data cleansing, which involved the removal of anomalies and missing values. The PyDS package was utilized to detect outliers in the data, employing the unsupervised method known as Isolation Forest (Eze et al., 2023; Lai et al., 2021; Laptev et al., 2015; Liu et al., 2008).

The study aimed to divide participants into groups based on changes in pupil size dynamics relative to task difficulty. Each participant is represented by a vector of 306 parameters, which are the averaged pupil sizes over the representations. Each element of this vector corresponds to the mean pupil size value across a presentation, thus representing the trajectory of pupil size fluctuations throughout the experiment. This pupil size vector underwent a smoothing process via convolution, using a window size aligned with the number of representations at each difficulty level.

We applied K-mean clustering to these vectors, resulting in two distinct clusters with significant differences in pupil size dynamics in response to task complexity. The K-Means algorithm (MacQueen, 1967) from the tslearn library (Tavenard et al., 2020) was employed for clustering, with Euclidian distance to measure the dissimilarity between vectors. The K-Means algorithm partitions data into a set number of clusters by assigning each point to the nearest centroid, iteratively adjusting centroids to minimize within-cluster variance until stabilization. The clustering resulted in two groups of participants with markedly different pupil size dynamics corresponding to the complexity of the task. Previously, the clustering algorithm (Shi et al., 2021; Yuan and Yang, 2019) was utilized in the pilot study (Alshanskaia and Martynova, 2023).

### 2.6 Statistical analysis

In this study, we computed descriptive statistics for two clusters, including the mean and standard deviation. The normality of the data distribution was assessed using Shapiro-Wilk tests. For normally distributed data, we utilized t-tests (with reported T-statistics and *p*-values) and estimated effect sizes using d, along with confidence intervals to assess statistical difference between two cluster ANS patterns and behavioral features. For data that did not follow a normal distribution, we applied non-parametric methods such as the Mann-Whitney U test (with reported test statistics and *p*-values). Effect sizes for non-normally distributed data were determined using Rank-biserial correlation (RBC), Common Language Effect Size (CLES), and the correlation coefficient (r), each with corresponding confidence intervals. For ranked parameters, the Chi-squared test was utilized. All analyses were conducted using Python with libraries such as NumPy (Harris et al., 2020), SciPy (Virtanen et al., 2020), and Pandas (McKinney, 2010), computational tools for statistical testing and effect size calculation.

## 3 Results

**FIGURE 1:**
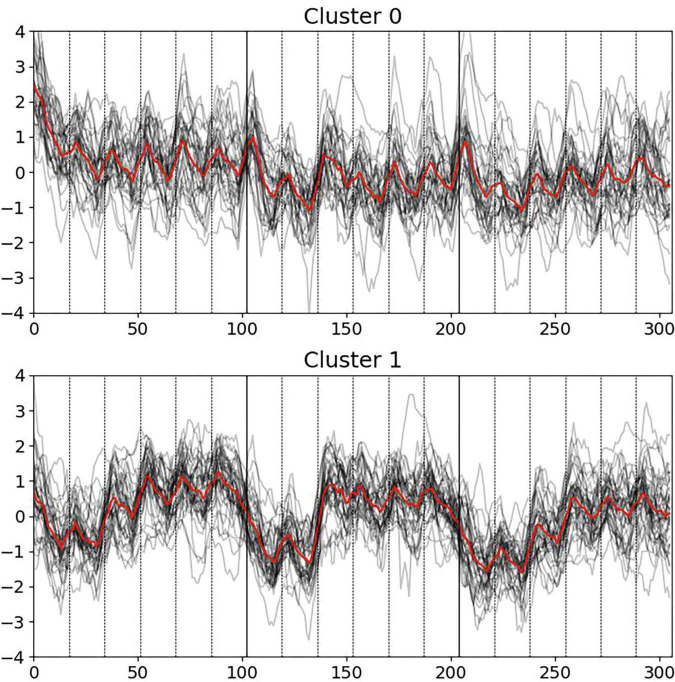
Pupillometry dynamics via k-means clustering across three blocks of six escalating cognitive levels each, with deceptive feedback in Block 2, levels 3 to 6. Cluster 0 exhibits significant initial responses at task onset. Cluster 1 shows pronounced pupillary responses to deceptive feedback in Block 2.

In the study, two distinct clusters (groups of individuals) were identified based on pupillometry dynamics (see Figure 1). Cluster 0 consisted of 33 participants (9 males, 24 females) with an average age of 24.6 ± 6.7 years old. Cluster 1 included 37 participants (16 males, 21 females), also with an average age of 27.6 ± 9.6 years old. These clusters were used to compare other psychological and vegetative parameters between them, to find distinct parameters and properties, and to see the general generalizing picture.

### 3.1 Psychological assessment results

For psychological and preliminary assessment, statistical significance was found in the Epworth Sleepiness Scale, indicating a higher average sleepiness level for Cluster 0 (9.78 ± 3.38) compared to Cluster 1 (8.03 ± 3.81). The comparison of smoking status between clusters also showed statistical significance (χ^2^ = 3.854, p = 0.049) (see Tables 1, 2). There was no statistical significance for sleep start time before the task, wake-up time before the task, time of sleep duration, Taylor anxiety scores, body mass index, Beck Depression scores, Spielberger State Anxiety scores, Spielberger Trait Anxiety, and deceptive feedback noticed after the task (which was taken at the end of the examination).

**TABLE 1:** Comparative analysis statistics of physiological and psychological variables between Cluster 0 and Cluster 1 by levels.

Variable	Cluster 0 mean (SD)	Cluster 1 mean (SD)	Statistics	Effect size (CI)	Additional statistics
Body mass index	21.7 (2.9)	22.3 (2.8)	*p*-value = 0.39 Student’s *t* = −0.87	d = −0.208 [−0.687, 0.271]	*r* = −0.122, CI *r* = [−0.356, 0.113]
Time of sleep duration (min)	469.8 (57.5)	466.4 (83.0)	*p*-value = 0.704 U = 624		RBC = −0.054, CLES = 0.527
Epworth’s test	9.8 (3.4)	8.0 (3.8)	*p*-value = **0.046** U = 738		RBC = −0.281, CLES = 0.641
Taylor anxiety	20.2 (8.4)	20.1 (9.2)	*p*-value = 0.916 U = 620		RBC = −0.016, CLES = 0.508
Beck Depression	9.6 (7.3)	9.1 (6.5)	*p*-value = 0.76 Student’s *t* = 0.31	d = 0.075 [−0.406, 0.557]	*r* = 0.005, CI *r* = [−0.231, 0.241]
Spielberger State Anxiety	43.7 (14.3)	41.8 (11.7)	*p*-value = 0.554 U = 605.5		RBC = −0.085, CLES = 0.543
Spielberger Trait Anxiety	44.2 (9.8)	45.3 (9.7)	*p*-value = 0.655 U = 522		RBC = 0.065, CLES = 0.468

The bold value indicates statistical significance observed in the Epworth’s test result, with a *p*-value = 0.046 (U = 738, RBC = –0.281, CLES = 0.641).

**TABLE 2:** Chi-square analysis of self-reported behaviors and conditions.

Self-Reported Information	χ^2^	*p*-value
Sleep start time before task (1−Early, 2−Late)	0.002	0.962
Wake up time before task (1−Early, 2−Late)	0.421	0.516
Smoking (0−No, 1−Yes)	**3.854**	**0.049**
History of head injury (0−No, 1−Yes)	3.674	0.055
Presence of sleep disorders (0−No, 1−Yes)	0.085	0.771
Deceptive feedback noticed (0−No, 1−Yes)	0.017	0.897

The bold value indicates statistical significance in the variable “Smoking (0–No, 1–Yes),” with a test statistic of 3.854 and a *p*-value = 0.049.

### 3.2 HRV metrics

#### 3.2.1 Time domains of heart rate variability

The MeanNN in Cluster 0 consistently displayed higher values than Cluster 1, reaching a peak at 870 ms in Block 2, Level 3 with the start of deceptive feedback. Significant statistical differences were observed in almost all blocks, except for Block 1, Level 5 and Block 2, Level 2 where the differences were not significant. Notably, both clusters peaked at Block 2, Level 3, when misleading deceptive feedback was introduced, Cluster 1 peaked at a lower value of 801 ms.

In the SDNN parameter, Cluster 0 also demonstrated higher values throughout the task, with significant differences especially notable at the beginning and end of the blocks. However, only Block 1 Level 5, Block 2 Level 2, Block 2 Level 4, and Block 2 Level 6 showed statistically significant differences. Block 3 presented the highest values for Cluster 0 with significant differences particularly evident in Levels 1, 4, and 5. No pronounced reaction to deception was observed.

**FIGURE 2:**
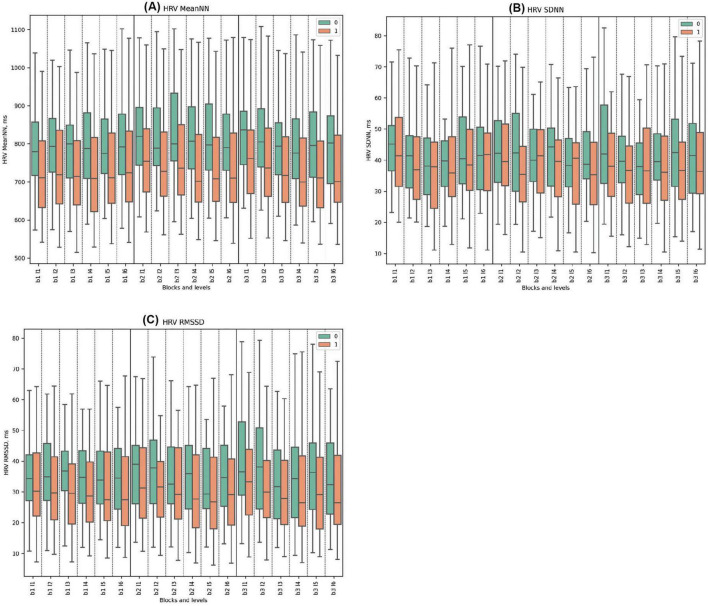
Time Domains of HRV Metrics. **(A)** HRV MeanNN for clusters 0 and 1, displaying mean normal-to-normal intervals (ms). **(B)** HRV SDNN showcasing variability between clusters across blocks, measured as the standard deviation of NN intervals (ms). **(C)** HRV RMSSD for clusters 0 and 1, highlighting short-term variability, calculated as the root mean square of successive differences (ms).

For the RMSSD metric, Cluster 0 demonstrated higher mean values than Cluster 1 across all experimental blocks and levels, with statistically significant differences observed in each instance. Specifically, Cluster 0 reached its peak RMSSD value of 49.6 ms during Block 3, Level 1, and recorded its minimum at 41.1 ms in Block 3, Level 6. By comparison, Cluster 1 attained its maximum mean RMSSD at 34.9 ms in Block 3, Level 1, and exhibited its minimum value of 29.6 ms in Block 3, Level 4. No pronounced physiological responses to deception were detected. (See Figure 2 and Supplementary Tables 1–3 for detailed statistical analysis for all time domains).

#### 3.2.2 Frequency domains of heart rate variability

For the HRV HF, Cluster 0 consistently showed higher values than Cluster 1 across all blocks. Significant statistical differences between the clusters were observed in Block 1, Levels 1, 3, 4. Similarly, in Block 2, significant differences were noted in Levels 1, 3; while in Block 3, Levels 3 and 4 demonstrated statistically significant disparities. The highest mean value for Cluster 0 was in Block 2 Level 1 (0.051) and the lowest was in Block 2 Level 6 (0.041). For Cluster 1, the highest mean was in Block 2 Level 2 (0.044), while the lowest was in Block 2 Level 3 (0.026) with the deceptive feedback with statistical significance between groups.

In terms of HRV LF, The both clusters showed increasing trends in HRV LF values across blocks, with Cluster 1 exhibiting higher peaks and greater variability, particularly in Block3. The only statistically significant difference was observed in Block 3, Level 1, indicating that Cluster 1 had higher HRV LF values. No pronounced reaction to deception was observed.

**FIGURE 3:**
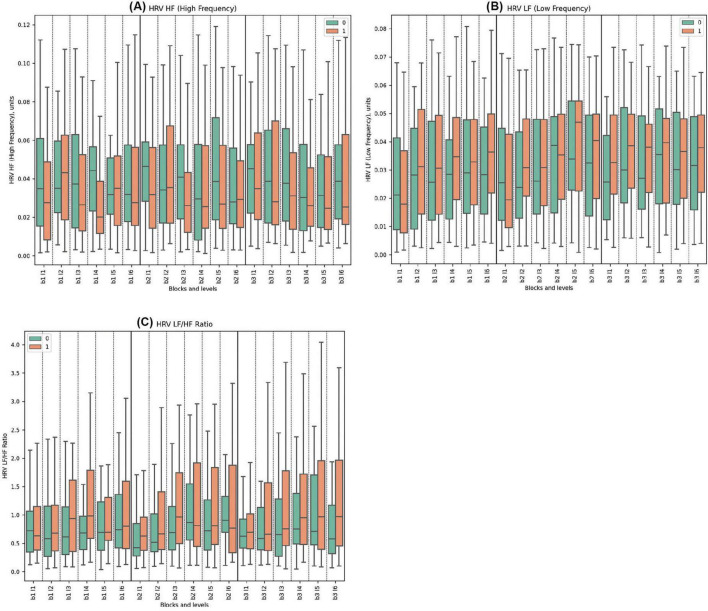
Frequency Domains of HRV Metrics. **(A)** HRV HF for clusters 0 and 1, showing high-frequency components, associated with parasympathetic activity. **(B)** HRV LF comparing low-frequency components across clusters, reflecting both sympathetic and parasympathetic influences. **(C)** LF/HF ratio depicting autonomic balance between clusters, a unitless measure.

Regarding the HRV LF/HF ratio, Cluster 0 consistently displayed lower mean values compared to Cluster 1 across all blocks. Statistically significant differences were found in Block 1, Levels 3 and 4; in Block 2, Levels 3, 5, and 6; and in Block 3, Levels 3, 4, 5, and 6. No specific reaction to deception was observed.

(See Figure 3 and Supplementary Tables 4–6 for detailed statistical analysis for all frequency domains).

### 3.3 Oculomotor metrics

#### 3.3.1 Normalized pupil size parameters

The data on normalized maximum pupil size indicates that Cluster 0 began each block with higher initial values compared to Cluster 1, which decreased and stabilized toward the end. Conversely, Cluster 1 generally displayed its lowest values at the start of the blocks, with trends increasing in response to rising difficulties. Significant differences were observed at the start of every block (Block 1, Levels 1 and 2; Block 2, Level 1; Block 3, Level 1) and during levels with deceptive feedback (Block 2, Levels 3, 4, 5, 6).

In the normalized pupil size mean measurements across all blocks, Cluster 0 consistently demonstrated higher mean values initially, followed by a pronounced decrease compared to Cluster 1, which showed increasing trends and greater variability. Statistically significant differences were noted in Block 1, Levels 1, 2, 5, and 6. For Block 2, both clusters peaked, with Cluster 0 reaching its maximum mean at Block 2, Level 3 with deceptive feedback. Cluster 1 peaked at the same level. Very significant differences were observed in Block 2, Levels 1, 3, 4, and 5. In Block 3, Cluster 0 exhibited variation from a low to a high until the end, while Cluster 1 demonstrated opposite trends. Significant differences were observed in Block 3, Levels 1 and 4.

**FIGURE 4:**
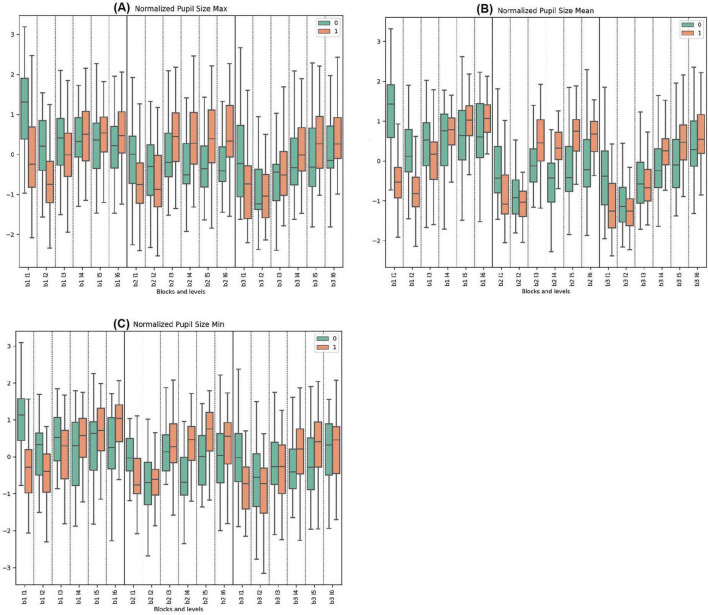
Oculomotor Metrics: Normalized Pupil Size. **(A)** Max normalized pupil size variations for clusters 0 and 1. **(B)** Mean normalized pupil size, showing average changes. **(C)** Min normalized pupil size, detailing minimum pupil size fluctuations.

In the normalized pupil size minimum parameters across all tasks, Cluster 0 consistently exhibited higher initial mean values with subsequent decrease and stabilization, in contrast to Cluster 1, which mirrored this behavior in other pupillometry properties. Pronounced statistically significant differences between the clusters were observed in Block 1, Levels 1, 4, and 6. For Block 2, Cluster 0 showed lower and less pronounced trends compared to Block 1, whereas Cluster 1 started lower and peaked towards the end. Significant disparities were noted in Block 2, Levels 1, 4, and 5. Similar trends were repeated in Block 3. Statistically significant differences were found in Block 3, Levels 1, 2, and 4. (See Figure 4 and Supplementary Tables 7–9 for detailed statistical analysis).

#### 3.3.2 Saccade parameters

Throughout all tasks, Cluster 1 exhibited higher mean values for saccade count compared to Cluster 0. Both groups showed a trend of increasing saccade counts from the beginning to the end of every block. Significant differences were noted in all levels except Block 1, Level 1, and Block 3, Level 6 (beginning and the end of the task).

For saccade velocity mean, Cluster 1 consistently demonstrated higher mean values in degrees per second (°/s) compared to Cluster 0 throughout all blocks. In Block 1, Cluster 0 showed a decreasing trend from the beginning to the end, which was the same for Cluster 1. Statistically significant differences between the clusters were identified only in Block 1, Level 4, with Cluster 0 having lower parameters. For Block 2, both clusters had the highest mean in Block 2, Level 3 with deceptive feedback, but without statistical differences. In Block 3, the mean values for both clusters ranged from high to low.

Throughout all blocks, Cluster 1 consistently exhibited higher mean values of saccade duration sum compared to Cluster 0. Both Cluster 0 and Cluster 1 demonstrated increasing trends. In Block 1, Cluster 0’s trend ranged from a minimum to a maximum, with an increasing difference that decreased afterward. Cluster 1 showed a similar trend but with a prolonged response. Statistically significant differences between the clusters were identified in Block 1 Level 5 and Level 6. In Block 2, both clusters peaked in Block 2 Level 3, at the start of deceptive feedback. Significant disparities were observed in all levels in Block 2, with the most significant difference in Block 2 Level 3 (*t*-test = −3.42, *p*-value = 0.001), where Cluster 0 had lower values. In Block 3, both clusters ranged from a low to a high. Significant differences were noted in all levels except the last one.

**FIGURE 5:**
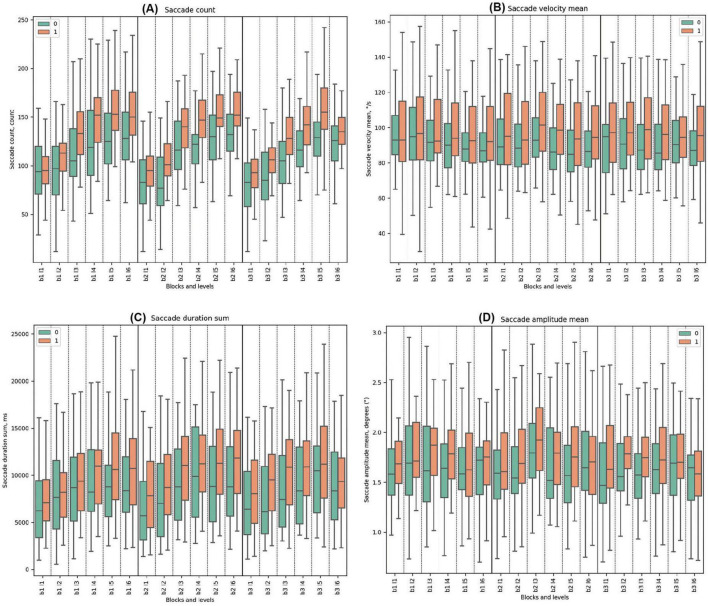
Saccade Metrics. **(A)** Saccade count across clusters, emphasizing eye movement frequency. **(B)** Mean saccade velocity, indicating average speed of saccades (degrees per second). **(C)** Sum of saccade durations, showing total time of saccades (ms). **(D)** Mean saccade amplitude, reflecting average magnitude of eye movements.

In all the blocks, Cluster 0 consistently demonstrated lower mean values of saccade amplitude mean compared to Cluster 1. Both clusters demonstrated pronounced fluctuations at the beginning of the task in the first levels. In Block 2, both clusters had the highest mean in Block 2 Level 3 with the start of deceptive feedback, but a statistically significant difference was observed only in Block 2 Level 5. In Block 3, significant differences were noted in Block 3 Level 2 and Block 3 Level 4. (See Figure 5 and Supplementary Tables 10–13 for detailed statistical analysis for all saccade properties).

#### 3.3.3 Fixation parameters

In all the blocks, Cluster 0 consistently exhibited higher mean values of fixation duration mean compared to Cluster 1. Both clusters had decreasing trends from the start to the end of every block. Statistically significant differences between the clusters were observed across all blocks. In Block 2 Level 3, where deception detection started, the most significant difference between groups was observed (t-test = 4.26, *p* = 0.0001).

**FIGURE 6:**
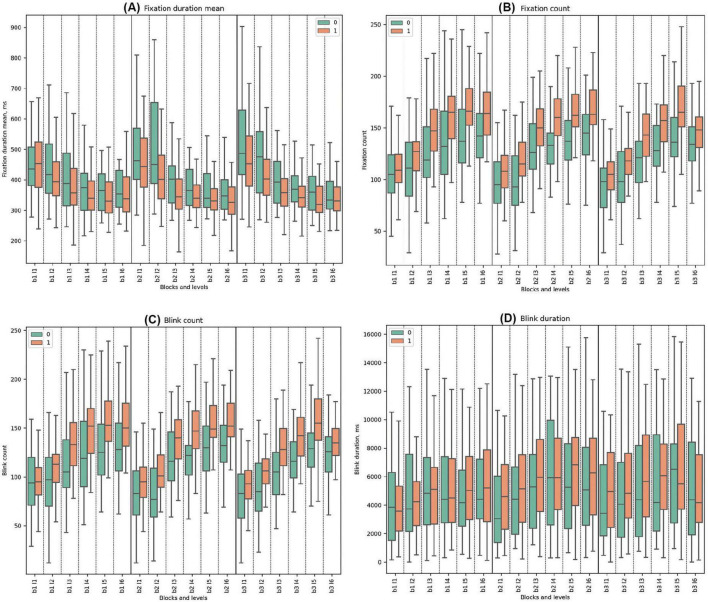
Fixation and Blink Metrics. **(A)** Mean fixation duration, highlighting average time of eye fixations (ms). **(B)** Fixation count, detailing number of fixations made. **(C)** Blink count, representing frequency of blinks. **(D)** Blink duration, showing average time of blinks (ms).

Cluster 1 consistently demonstrated higher mean values of fixation count compared to Cluster 0, with a rising trend for both clusters. Significant differences were observed throughout all blocks except in the initial Block 1 Level 1. The highest significance was especially noted in levels with the false feedback: Block 2 Level 3 (t-test = −4.58, *p* = 0.00002), Block 2 Level 4 (U-test = 248.5, *p* = 0.00002), Block 2 Level 5 (U-test = 275.5, *p* = 0.00008), and Block 2 Level 6 (t-test = −3.56, *p* = 0.001). In Block 3, the trends indicate a gradual increase in mean values for both clusters across the block. (See Figure 6 and Supplementary Tables 14, 15 for detailed statistical analysis for fixation properties).

#### 3.3.4 Blink parameters

Cluster 1 demonstrated higher mean blink counts compared to Cluster 0 throughout all blocks. Both clusters exhibited increasing trends from the beginning to the end of each block. Statistically significant differences between the clusters were identified in all blocks and levels except for Block 1 Level 1 and Block 3 Level 6. In the deceptive levels (beginning in Block 2 Level 3), statistical significance became pronounced.

In all three blocks, Cluster 1 consistently had higher blink durations across the study except for the initial Level 1 in Block 1, with significant disparities in the early segments of Blocks 2 and 3. During Block 1, both clusters demonstrated a similar slightly increasing and fluctuating pattern. No statistically significant differences were observed between the clusters in Block 1. In Block 2, the trend was similar to the first block, with statistical significance observed only in Block 2 Level 1. At the beginning of Block 3, Cluster 0 had statistically significantly lower blink durations compared to Cluster 1. Statistically significant differences between the clusters were found in Block 3 Levels 1, 2, and 3 (See Figure 6 and Supplementary Tables 16, 17 for detailed statistical analysis of blink properties).

### 3.4 Cognitive metrics

#### 3.4.1 Self-esteem

Cluster 1 demonstrated higher self-esteem parameters compared to Cluster 0 throughout all blocks, with the exceptions of Block 1, Level 1, and Block 3, Levels 1 and 6. In both clusters, self-esteem decreased with the increase in difficulty. The lowest self-esteem score for Cluster 0 was observed in Block 2, Level 6 (2.2) at the end of the deceptive period, and the highest was in Block 3, Level 1 (4.8). For Cluster 1, the highest scores were in Block 2, Levels 1 and 2 (4.8), and the lowest was in Block 2, Level 6 (2.4), similar to the trends in Cluster 0. The only statistically significant difference between the clusters was in Block 1, Level 2 (t-test = −2.07, p = 0.04), Block 2, Level 2, and Block 2, Level 3 at the start of the deceptive trial. In Block 3, no significant differences were noted (see the table with the detailed statistics in the Supplementary Table 18).

#### 3.4.2 Response time

Regarding the time of response, Cluster 0 demonstrated lower mean values compared to Cluster 1, with one exception in Block 1 Level 1. Both clusters demonstrated increasing trends in time with rising difficulty. The slowest reaction time for Cluster 0 was in Block 2 Level 6 (end of the deceptive trial), while the fastest reaction time was observed in Block 3 Level 1 (beginning of the last block in the task). For Cluster 1, the fastest reaction time was observed in Block 3 Level 4, while the highest mean was at the end of the trial with deceptive falls feedback in Block 2 Level 6, similar to the other cluster.

**FIGURE 7:**
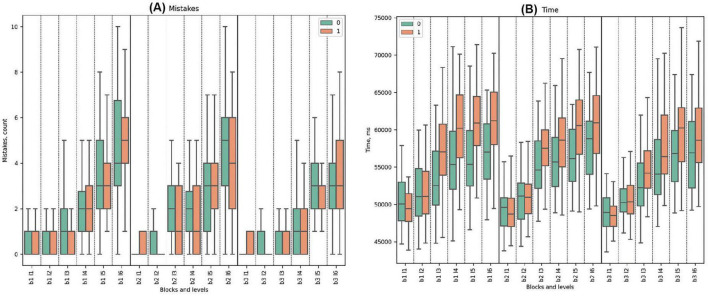
Performance Metrics: Mistakes and Time. **(A)** Mistake count per cluster across tasks. **(B)** Time taken for task completion, showing efficiency (ms).

Statistically significant differences between the clusters were observed in Block 1 Levels 3, 4, 5, and 6; Block 2 Levels 3, 4, and 5 (false feedback trial); and Block 3 Levels 2, 3, 4, and 5. There was no statistical significance at the beginning of every block and at the end of the deceptive trial. Total_Time exhibited higher mean values for Cluster 1 compared to Cluster 0, with a statistically significant difference (*p*-value = 0.003), where Cluster 0 had a pronounced shorter reaction time. See Figure 7 and Supplementary Table 19 for detailed statistical analysis of time properties.

#### 3.4.3 Mistakes

Cluster 0 generally demonstrated a higher mistake rate than Cluster 1 across all tasks. Across the entire dataset, both clusters demonstrated increasing trends, with the highest mistake rate at the end of the blocks (see Figure 7). Significant differences were noted in Block 2 Level 2 (*U*-test = 707.5, *p*-value = 0.043). Detailed statistical analysis of mistake properties is provided in the Supplementary Table 20.

### 3.5 Other ANS metrics

**FIGURE 8:**
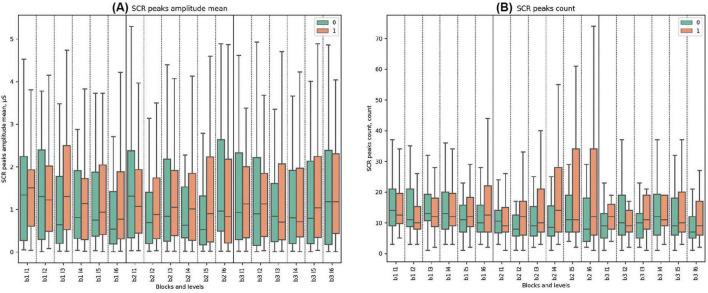
SCR (Skin Conductance Response) Metrics. **(A)** Mean amplitude of SCR peaks, detailing physiological response levels (microsiemens, μS). **(B)** Count of SCR peaks, highlighting frequency of responses.

In the present study, no statistically significant differences between clusters were observed for all analyzed respiratory and skin conductance response (SCR) parameters (see Figure 8, Supplementary Figures 7, 8 and Supplementary Tables 21–27 for detailed statistical analysis of ANS metrics in SGR dynamics).

### 3.6 False feedback detection

Regarding reactions to false feedback, individuals from Cluster 1 demonstrated distinct peaking reactions compared to Cluster 0. HRV MeanNN peaked in Block 2 Level 3 (870 ms) in cluster 1, showing a statistically significant difference from cluster 0. Unique to cluster 1 were the minimum HRV HF (0.026) and maximum normalized pupil size (0.71), both in Block 2 Level 3, without corresponding peaks in cluster 0. Cluster 1 also peaked in saccade velocity mean (107.7 °/s) in Block 2 Level 3, though this was not statistically different from cluster 0. Both clusters peaked in saccade amplitude mean in Block 2 Level 3, with cluster 0 at 1.88 and cluster 1 at 2.03, but without statistical significance. These findings highlight that cluster 1 exhibits more pronounced peaking parameters and distinct autonomic and ocular responses, suggesting a more reactive profile compared to cluster 0 (see the tables with the statistics in the Supplementary Tables 1–27).

### 3.7 Parameters by blocks

The generalized analysis of the data was conducted by blocks. This analysis identified several parameters with statistically significant differences between clusters, as indicated by *p*-values less than or equal to 0.05. The HRV MeanNN showed significance in block 1 (*p*-value = 0.044), block 2 (*p*-value = 0.017), and block 3 (*p*-value = 0.015). The HRV SDNN was significant in block 3 (*p*-value = 0.030). For HRV RMSSD, significance was found in block 1 (*p*-value = 0.003), block 2 (*p*-value = 0.010), and block 3 (*p*-value = 0.008). The HRV HF demonstrated significance in block 1 (*p*-value = 0.001), block 2 (*p*-value = 0.008), and block 3 (*p*-value = 0.034). The HRV LF/HF parameter showed significance in block 1 (*p*-value = 0.012), block 2 (*p*-value = 0.004), and block 3 (*p*-value = 0.003). The normalized pupil size mean parameter exhibited significance in block 2 (*p*-value = 0.001) and block 3 (*p*-value = 0.023).

For saccade count, blocks 1, 2, and 3 all showed significance with *p*-values ≤ 0.000. The saccade duration sum parameter displayed significance in block 1 (*p*-value = 0.043), block 2 (*p*-value = 0.002), and block 3 (*p*-value = 0.004). The fixation duration mean parameter was significant in block 1 (*p*-value = 0.001), block 2 (*p*-value = 0.000), and block 3 (*p*-value = 0.000). The fixation count for blocks 1, 2, and 3 all displayed significance with *p*-values of 0.000. Similarly, blink count for blocks 1, 2, and 3 all showed significance with *p*-values of 0.000. Finally, the Sum Time parameter showed significance in block 1 (*p*-value = 0.004), block 2 (*p*-value = 0.003), and block 3 (*p*-value = 0.004). See the tables with the statistics and figures in the Supplementary Tables 1–27, and Supplementary Figures 1–8.

## 4 Discussion

**FIGURE 9:**
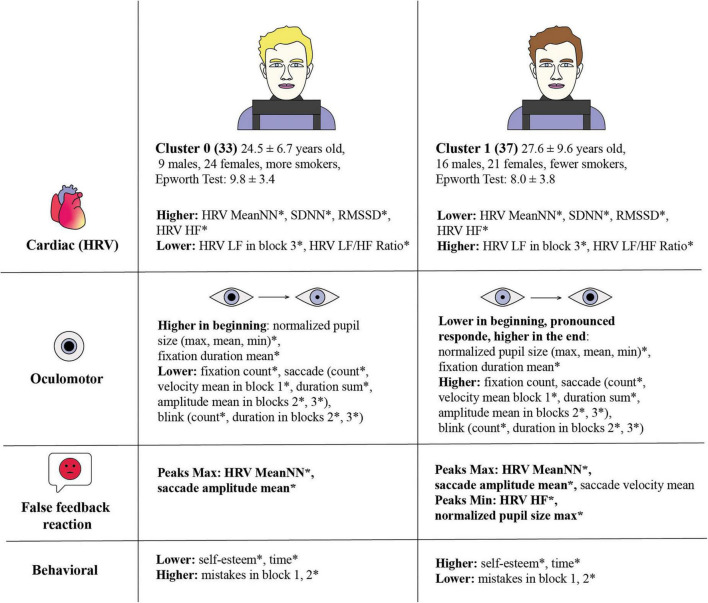
Comparative analysis of cardiac, oculomotor, and behavioral responses across two clusters, revealing distinct physiological and psychological patterns related to stress responses. Statistical significance between clusters is marked by an asterisk (*). Cluster 0 consists of 33 individuals, average age 24.5 ± 6.7 years, with 9 males and 24 females, and more smokers. Cluster 0 exhibits higher HRV MeanNN*, SDNN*, RMSSD*, HRV HF*, and HRV LF in block 3*, HRV LF/HF Ratio*. The Epworth Test score is 9.78 ± 3.38. Cluster 0 has higher normalized pupil size (max, mean, min)* and fixation duration mean* at the beginning, and lower fixation count*, saccade count*, velocity mean in block 1*, duration sum*, amplitude mean in blocks 2*, 3* and blink count*, duration in blocks 2*, 3*. Cluster 0 False Feedback Reaction with pronounced extremes throughout all the blocks with Peaks Max: HRV MeanNN*, saccade amplitude mean. Cluster 0 has lower self-esteem* and time*, but higher mistakes in block 1, 2*. Cluster 1 consists of 37 individuals, average age 27.6 ± 9.6 years, with 16 males and 21 females, and fewer smokers. The Epworth Test score is 8.03 ± 3.81. Cluster 1 shows lower mean of HRV MeanNN*, SDNN*, RMSSD*, HRV HF*, HRV LF in block 3*, HRV LF/HF Ratio*. Cluster 1 has lower normalized pupil size (max, mean, min)* and fixation duration mean* at the beginning with a pronounced response and higher values at the end. They have higher fixation count*, saccade count*, velocity mean in block 1*, duration sum*, amplitude mean in blocks 2*, 3*, and blink count*, duration in blocks 2*, 3*. Cluster 1 False Feedback Reaction with pronounced extremes throughout all the blocks with Peaks Max: HRV MeanNN*, saccade amplitude mean*, saccade velocity mean; Peaks Min: HRV HF*, normalized pupil size max*. Cluster 1 has higher self-esteem* and time*, but lower mistakes in block 1, 2*.

Our study investigated the relationship between pupil dilation dynamics under cognitive load and various physiological and behavioral responses, grouped into distinct clusters and analyzed by levels. The findings reveal significant differences between the clusters, particularly in HRV indices, oculomotor behavior, and reactions to false feedback (Figure 9). The study links oculomotor metrics with heart rate, aligned with previous research (Hoogerbrugge et al., 2022). We assume the data analysis by levels provides more detailed information on statistical differences and the distinctions between Cluster 0 and Cluster 1. When considering more general indicators by block rather than by level, some statistically significant differences are not as well captured. However, the key differences remain statistically significant when analyzing the data by block.

Individuals in Cluster 0, characterized by their pupil dilation dynamics under cognitive load, exhibited higher HRV indices. Specifically, Cluster 0 demonstrated higher values in MeanNN, SDNN, RMSSD, and HRV HF, and lower values in HRV LF and the HRV LF/HF ratio in Block 3. These results suggest a predominance of parasympathetic activity in Cluster 0, indicative of a more relaxed autonomic state (Pham et al., 2021). Elevated parasympathetic tone and HRV indices imply greater cardiovascular stability and a superior ability to adapt to stress, reflecting efficient autonomic regulation.

Conversely, Cluster 1 showed lower values in HRV MeanNN, SDNN, RMSSD, and HRV HF, with higher values in HRV LF and the HRV LF/HF ratio in Block 3. This pattern is indicative of sympathetic dominance, reflecting higher stress levels (Shaffer and Ginsberg, 2017). The elevated sympathetic activity in Cluster 1 could correspond to a heightened state of alertness or arousal, potentially linked to sustained cognitive effort or stress. The differential autonomic responses between the clusters highlight the variability in individual physiological adaptation to cognitive load, which might be aligned with lower rates of Epworth Sleepiness Scale scores.

Cluster 0’s higher normalized pupil sizes (max, mean, min) and fixation duration mean at the beginning of the task also support the interpretation of initial heightened arousal and cognitive engagement. The following decrease in these parameters may be explained by losing engagement in cognitive load or be consistent with the physiological autonomic shift (Ferencová et al., 2021).

Differences in oculomotor behavior further elucidate the distinct autonomic profiles of the clusters. Cluster 0 exhibited a lower fixation count, saccade (count and velocity, amplitude, and duration), and blink parameters. These metrics suggest a shift towards parasympathetic dominance and reduced autonomic support, indicating a transition to a more relaxed state as the task progresses, which may be caused by physiological specificity or disengagement over time, aligning with a shift to a more energy-efficient autonomic state (Skaramagkas et al., 2023).

Cluster 1, on the other hand, exhibited lower normalized pupil size and fixation duration mean initially but showed increased responses later. Their higher fixation count, saccade count, and other metrics throughout the task suggest sustained or increased cognitive engagement and SNS activation (Steinhauer et al., 2004). This could reflect a persistent or escalating cognitive load, possibly driven by different motivational or regulatory mechanisms. The delayed but sustained oculomotor activity aligns with their sympathetic dominance and lower HRV indices, indicating prolonged cognitive effort and engagement.

Both clusters showed peaks in HRV MeanNN and saccade amplitude mean in response to false feedback, with significant differences observed. An increased MeanNN (R-R interval) is indicative of a slower heart rate within physiological norms, whereas a decreased interval corresponds to a faster heart rate or lower HRV. Furthermore, increased cognitive or stress load results in the decreased HRV (Forte et al., 2019; Arakaki et al., 2023). Cluster 1 also exhibited statistically significant lower peaks in HRV HF and normalized pupil size max during deception. This suggests a more pronounced physiological response to false feedback in Cluster 1, characterized by increased sympathetic activity and acute parasympathetic modulation. The lower HRV HF indicates a diminished capacity for vagal regulation, while the decreased pupil size max reflects heightened cognitive and emotional arousal, consistent with a greater stress response to deception. These results add an additional contribution to already existing data (Celniak et al., 2023; Webb et al., 2009).

Behaviorally, individuals in Cluster 0 reported lower self-esteem, faster reaction times, and a higher number of mistakes, particularly with statistical significance in Block 2. These behavioral traits may be linked to their physiological profile, indicating a higher level of impulsivity or reduced cognitive control (Marshall, 2007), as well as lack of motivation. The initial high arousal followed by a decline in engagement could lead to increased errors and lower performance consistency, reflecting a potential drop in sustained attention and cognitive resources. Cluster 0 participants, characterized by elevated HRV indices and heightened oculomotor arousal at the beginning of tasks, exhibited a decline in engagement over time. This decline was evident in reduced time spent on task execution, less pronounced reactions to false feedback, increased errors, and reduced performance consistency. This pattern suggests a reduction in sustained attention and cognitive resources, potentially linked to diminished cognitive control due to a more relaxed condition and lower engagement. Consequently, these participants demonstrated lower performance and self-esteem.

Cluster 1, in contrast, reported higher self-esteem, longer reaction times, and fewer mistakes, suggesting better cognitive control and sustained engagement. The link of lower HRV and longer time of the task aligned with previous research (Tinello et al., 2022). Their physiological responses, characterized by sympathetic dominance and sustained oculomotor activity, support the interpretation of persistent cognitive effort and higher engagement levels. Cluster 1 participants, with lower HRV indices and sustained oculomotor activity, maintained prolonged cognitive engagement and spent more time completing tasks. They exhibited higher stress levels (lower HRV), which were reflected in better task performance, fewer mistakes, and more stable self-esteem. The observed discrepancies between clusters might be attributed to the optimal level of stress required for effective task solving, as posited by the Yerkes-Dodson law (Elbćk et al., 2022; Calabrese, 2008). Performance increases with physiological or mental arousal but only up to a point. When arousal becomes excessive, performance diminishes (Portnova et al., 2023; Awada et al., 2024). Cluster 1’s lower HRV and higher arousal levels may indicate that their autonomic nervous system (ANS) was in a more optimal state for cognitive tasks, facilitating better cognitive control and sustained effort. The relationship between self-esteem and task execution might be more closely associated with cognitive-behavioral factors than purely autonomic parameters. Effective performance may depend more on the ANS’s ability to switch efficiently between states rather than simply maintaining high HRV parameters. Future research should explore these dynamics, considering both physiological and cognitive-behavioral aspects to understand the complex interplay in cognitive task performance with a personalized approach.

K-means clustering based on pupil size changes under cognitive load revealed distinct behavioral traits and significant differences in ANS reactions and eye-movement patterns among individuals. Cluster 0 demonstrated higher HRV related to parasympathetic activity and an initial sympathetic response, suggesting an efficient but transient engagement. In contrast, Cluster 1 showed lower HRV and sustained oculomotor activity, indicating prolonged engagement and higher cognitive load. Cluster 0 exhibited higher HRV indices and a transition to a relaxed state, indicating parasympathetic dominance, while Cluster 1 showed lower HRV indices and sustained cognitive engagement, reflecting sympathetic dominance and higher stress or engagement levels. Both clusters demonstrated distinct responses to false feedback, with Cluster 1 displaying a more pronounced physiological stress response.

In conclusion, this study enhances our understanding of the relationship between ANS responses and cognitive load, particularly regarding stress and deception detection. The distinct modulation of sympathetic and parasympathetic activities provides valuable physiological markers, contributing to more precise diagnostic and therapeutic approaches. Integrating these findings into clinical practices and wearable technology can improve real-time monitoring and personalized mental health support, advancing precision therapies and continuous patient well-being monitoring.

### 4.1 Limitations

The present study, while offering valuable insights into the modulation of ANS responses during cognitive load and deception detection using K-means clustering for precision and personalized assessment, is constrained by several limitations. The variability in individual physiological and psychological responses underscores the need for larger and more diverse participant groups in future research. The reliance on self-reported data introduces potential biases, necessitating more detailed research and refinement to ensure data accuracy and interpretation. Despite instructing participants to abstain from smoking and caffeine-containing substances for 12 h before the procedure and confirming their compliance before the task, future research should include non-invasive pre-experiment baseline assessments to objectively verify abstinence and enhance the reliability of the results. Methodological challenges, particularly in handling signal noise, also present limitations. While preprocessing these signals is crucial for data clarity, it may inadvertently lead to the loss of nuanced physiological information. Despite these limitations, the study offers valuable contributions to the understanding of autonomic and oculomotor responses under cognitive load and deception detection, as well as the coherence of autonomic systems with behavioral outcomes. The distinct physiological and behavioral responses provide an additional instrument for non-invasive signals in distance assessment with a personalized approach. Future research should aim to integrate advanced technologies such as artificial intelligence and machine learning to enhance data accuracy and interpretation. It is essential to acknowledge the ethical considerations associated with the use of physiological data and real-time analysis.

Our findings on pupillary dynamics and ANS patterns, including HRV, can be translated into clinical practice and various applications in industry, education, and the workplace. This can be achieved through the development of remote personalized diagnostic tools for real-time monitoring of cognitive stress and performance. These tools would enable clinicians and therapists in psychology, psychiatry, and neurology to understand personalized ANS dynamics and adjust therapeutic interventions promptly based on physiological and cognitive feedback. Additionally, integrating these diagnostic tools into wearable technology or ethical remote online webcam analysis would allow continuous monitoring of patients or participants. This supports the development of precision therapies, improves care, and enhances self-care. This approach can be applied to online education or analyzing and predicting personalized cognitive performance at work, thereby managing cognitive stress and optimizing cognitive performance. These applications have the potential to enhance mental health support and patient well-being.

The potential for misuse of these technologies in web or wearable device surveillance or unauthorized monitoring poses significant ethical risks. Ensuring robust guidelines for the ethical application of such technologies is paramount, emphasizing the need for informed consent, data privacy, and the ability to immediately recall access to personal data, thereby preventing coercive practices. Future research should focus on developing ethical frameworks that safeguard individual autonomy and privacy while promoting the responsible use of physiological monitoring tools.

## Data Availability

The datasets presented in this study can be found in online repositories. Data publicly available on Mendeley Data: Liaukovich, Krystsina; Portnova, Galina; Alshanskaia, Evgenia; Martynova, Olga (2024), “Eyetracking and vegetatics data in a cognitive load task”, Mendeley Data, V1, doi: 10.17632/dnk4s6dtcf.
