# Supplementary material for: Pupillometry and autonomic nervous system responses to cognitive load and false feedback: an unsupervised machine learning approach
Source: Front Neurosci. 2024 Aug 30;18:1445697. doi: 10.3389/fnins.2024.1445697 (PMC11405740; doi:10.3389/fnins.2024.1445697)

Supplementary Material

Pupillometry and Autonomic Responses to Cognitive Load and False Feedback: An Unsupervised Machine Learning Approach

Evgeniia I. Alshanskaia[1], **Galina V. Portnova**[2], **Krystsina Liaukovich**[2], **Olga V. Martynova**[3]

[1]School of Psychology, Faculty of Social Sciences, National Research University Higher School of Economics, Moscow, Russia
[2] Institute of Higher Nervous Activity and Neurophysiology of the Russian Academy of Sciences
[3] Centre for Cognition and Decision Making, Institute for Cognitive Neuroscience, National Research University Higher School of Economics, Moscow, Russia

*** Correspondence:**
Evgeniia I. Alshanskaia
eisokolova@hse.ru

1 Supplementary Tables

Table 1. Comparative analysis statistics of physiological and psychological variables between Cluster 0 and Cluster 1 by levels.

Variable	Cluster 0 mean (SD)	Cluster 1 mean (SD)	Statistics	Effect Size (CI)	Additional statistics
Body mass index	21.7 (2.9)	22.3 (2.8)	p-value = 0.39 Student's t=-0.87	d=-0.208 [-0.687, 0.271]	r=-0.122, CI r=[-0.356, 0.113]
Time of sleep duration (min)	469.8 (57.5)	466.4 (83.0)	p-value = 0.704 U=624		RBC=-0.054, CLES=0.527
Epworth's test	9.8 (3.4)	8.0 (3.8)	p-value = **0.046** U=738		RBC=-0.281, CLES=0.641
Taylor anxiety	20.2 (8.4)	20.1 (9.2)	p-value = 0.916 U=620		RBC=-0.016, CLES=0.508

Beck Depression	9.6 (7.3)	9.1 (6.5)	p-value = 0.76 Student's t=0.31	d=0.075 [-0.406, 0.557]	r=0.005, CI r=[-0.231, 0.241]
Spielberger State Anxiety	43.7 (14.3)	41.8 (11.7)	p-value = 0.554 U=605.5		RBC=-0.085, CLES=0.543
Spielberger Trait Anxiety	44.2 (9.8)	45.3 (9.7)	p-value = 0.655 U=522		RBC=0.065, CLES=0.468

Table 2. Chi-square analysis of self-reported behaviors and conditions.

**Self-Reported Information**	χ^2	**p-value**
Sleep start time before task (1 - Early, 2 - Late)	0.002	0.962
Wake up time before task (1 - Early, 2 - Late)	0.421	0.516
Smoking (0 - No, 1 - Yes)	**3.854**	**0.049**
History of head injury (0 - No, 1 - Yes)	3.674	0.055
Presence of sleep disorders (0 - No, 1 - Yes)	0.085	0.771
Deceptive feedback noticed (0 - No, 1 - Yes)	0.017	0.897

2 Supplementary Statistical Data

HRV time domains

MeanNN

Table S1. HRV Time Domain MeanNN by levels.

Variable	Cluster 0 mean (SD)	Cluster 1 mean (SD)	Statistics	Metrics
**Block 1 Level 1**	823 (124)	754 (152)	**U-test = 775, p-value = 0.03**	RBC = -0.305, CLES = 0.652
**Block 1 Level 2**	834 (134)	768 (161)	**U-test = 767, p-value = 0.038**	RBC = -0.291, CLES = 0.646
**Block 1 Level 3**	828 (139)	753 (159)	**U-test = 744, p-value = 0.04**	RBC = -0.292, CLES = 0.646
**Block 1 Level 4**	834 (125)	758 (162)	**U-test = 800, p-value = 0.014**	RBC = -0.347, CLES = 0.673
Block 1 Level 5	831 (132)	773 (168)	U-test = 728, p-value = 0.063	RBC = -0.264, CLES = 0.632
**Block 1 Level 6**	839 (134)	774 (169)	**U-test = 741, p-value = 0.043**	RBC = -0.286, CLES = 0.643
**Block 2 Level 1**	856 (130)	797 (158)	**U-test = 756, p-value = 0.049**	RBC = -0.277, CLES = 0.639
Block 2 Level 2	847 (142)	789 (157)	U-test = 753, p-value = 0.053	RBC = -0.272, CLES = 0.636
**Block 2 Level 3**	870 (122)	801 (161)	**U-test = 777, p-value = 0.026**	RBC = -0.313, CLES = 0.656
**Block 2 Level 4**	847 (115)	775 (170)	**U-test = 800, p-value = 0.013**	RBC = -0.351, CLES = 0.676
**Block 2 Level 5**	852 (127)	777 (170)	**t-test = 2.10, p-value = 0.04**	d = 0.496, CI d [0.010, 0.981]; r = 0.292, CI r [0.058, 0.526]
**Block 2 Level 6**	843 (116)	780 (166)	**U-test = 801, p-value = 0.025**	RBC = -0.312, CLES = 0.656
**Block 3 Level 1**	855 (106)	786 (145)	**U-test = 804, p-value = 0.012**	RBC = -0.354, CLES = 0.677
**Block 3 Level 2**	850 (119)	786 (157)	**U-test = 796.5, p-value = 0.029**	RBC = -0.305, CLES = 0.652
**Block 3 Level 3**	833 (120)	750 (131)	**U-test = 812, p-value = 0.009**	RBC = -0.367, CLES = 0.684
**Block 3 Level 4**	827 (123)	753 (156)	**t-test = 2.21, p-value = 0.03**	d = 0.522, CI d [0.036, 1.007]; r = 0.288, CI r [0.053, 0.522]
**Block 3 Level 5**	840 (120)	762 (158)	**U-test = 826, p-value = 0.011**	RBC = -0.353, CLES = 0.676

**Block 3 Level 6**	835 (122)	765 (158)	**t-test = 2.09, p-value = 0.04**	d = 0.493, CI d [0.008, 0.978]; r = 0.303, CI r [0.069, 0.537]

SDNN

Table S2. HRV Time Domain SDNN by levels.

Variable	Cluster 0 mean (SD)	Cluster 1 mean (SD)	Statistics	Metrics
Block 1 Level 1	47.7 (15.2)	43.7 (15.2)	U-test = 663, p-value = 0.288	RBC = -0.151, CLES = 0.576
Block 1 Level 2	44.8 (14.9)	38.6 (14.2)	U-test = 720, p-value = 0.078	RBC = -0.250, CLES = 0.625
Block 1 Level 3	42.9 (15.4)	36.0 (14.5)	U-test = 688, p-value = 0.103	RBC = -0.233, CLES = 0.616
Block 1 Level 4	44.6 (16.7)	37.9 (15.3)	U-test = 725, p-value = 0.068	RBC = -0.259, CLES = 0.629
**Block 1 Level 5**	47.1 (13.6)	38.9 (15.1)	**U-test = 745, p-value = 0.019**	RBC = -0.335, CLES = 0.668
Block 1 Level 6	43.4 (13.5)	39.4 (14.6)	U-test = 635, p-value = 0.336	RBC = -0.138, CLES = 0.569
Block 2 Level 1	49.1 (17.5)	40.8 (14.9)	U-test = 721, p-value = 0.070	RBC = -0.257, CLES = 0.629
**Block 2 Level 2**	50.5 (22.4)	35.6 (14.3)	**U-test = 824, p-value = 0.002**	RBC = -0.437, CLES = 0.718
Block 2 Level 3	47.8 (18.7)	42.1 (17.8)	U-test = 643, p-value = 0.270	RBC = -0.159, CLES = 0.579
**Block 2 Level 4**	48.1 (18.7)	37.7 (14.1)	**U-test = 741, p-value = 0.019**	RBC = -0.335, CLES = 0.668
Block 2 Level 5	44.7 (17.7)	37.2 (15.9)	t-test = 1.83, p-value = 0.07	d = 0.447, CI d [-0.044, 0.938]; r = 0.206, CI r [-0.032, 0.443]
**Block 2 Level 6**	48.1 (19.7)	38.0 (17.8)	**t-test = 2.23, p-value = 0.03**	d = 0.542, CI d [0.052, 1.033]; r = 0.280, CI r [0.044, 0.516]
**Block 3 Level 1**	52.9 (21.8)	39.1 (14.9)	**t-test = 3.01, p-value = 0.001**	d = 0.747, CI d [0.249, 1.245]; r = 0.349, CI r [0.113, 0.585]
**Block 3 Level 2**	47.3 (22.4)	36.7 (15.1)	**t-test = 2.27, p-value = 0.03**	d = 0.564, CI d [0.073, 1.055]; r = 0.256, CI r [0.020, 0.492]
Block 3 Level 3	42.8 (17.3)	39.4 (18.7)	t-test = 0.80, p-value = 0.43	d = 0.192, CI d [-0.291, 0.675]; r = 0.110, CI r [-0.126, 0.346]
**Block 3 Level 4**	45.4 (13.5)	36.1 (14.6)	**t-test = 2.76, p-value = 0.01**	d = 0.663, CI d [0.168, 1.158]; r = 0.324, CI r [0.089, 0.560]
**Block 3 Level 5**	48.2 (17.4)	38.2 (15.5)	**t-test = 2.51, p-value = 0.01**	d = 0.611, CI d [0.118, 1.104]; r = 0.274, CI r [0.038, 0.510]

Block 3 Level 6	46.2 (20.1)	38.0 (15.2)	t-test = 1.89, p-value = 0.06	d = 0.465, CI d [-0.023, 0.953]; r = 0.191, CI r [-0.045, 0.427]

RMSSD

Table S3. HRV Time Domain RMSSD by levels.

**Variable**	**Cluster 0 mean (SD)**	**Cluster 1 mean (SD)**	**Statistics**	**Metrics**
Block 1 Level 1	41.0 (15.8)	31.1 (15.7)	**U-test = 773, p-value = 0.016**	RBC = -0.342, CLES = 0.671
Block 1 Level 2	42.1 (18.5)	31.5 (15.4)	**U-test = 779, p-value = 0.013**	RBC = -0.352, CLES = 0.676
Block 1 Level 3	41.7 (15.5)	29.1 (16.5)	**U-test = 823, p-value = 0.001**	RBC = -0.475, CLES = 0.737
Block 1 Level 4	41.2 (16.5)	29.8 (16.8)	**U-test = 817, p-value = 0.003**	RBC = -0.418, CLES = 0.709
Block 1 Level 5	41.8 (16.0)	31.8 (18.2)	**U-test = 781, p-value = 0.005**	RBC = -0.400, CLES = 0.700
Block 1 Level 6	38.2 (12.4)	31.5 (18.3)	**U-test = 699, p-value = 0.041**	RBC = -0.294, CLES = 0.647
Block 2 Level 1	47.7 (22.8)	33.6 (17.1)	**U-test = 815, p-value = 0.003**	RBC = -0.421, CLES = 0.711
Block 2 Level 2	49.1 (30.9)	31.8 (17.0)	**U-test = 829, p-value = 0.002**	RBC = -0.446, CLES = 0.723
Block 2 Level 3	44.2 (24.5)	32.9 (20.7)	**U-test = 744, p-value = 0.017**	RBC = -0.341, CLES = 0.670
Block 2 Level 4	43.1 (22.1)	30.9 (17.9)	**U-test = 751, p-value = 0.014**	RBC = -0.353, CLES = 0.677
Block 2 Level 5	40.5 (18.8)	31.8 (21.4)	**t-test = 1.79, p-value = 0.08**	d = 0.430, CI d [-0.062, 0.922]; r = 0.301, CI r [0.063, 0.539]
Block 2 Level 6	45.1 (24.4)	31.8 (20.4)	**t-test = 2.43, p-value = 0.02**	d = 0.594, CI d [0.102, 1.086]; r = 0.339, CI r [0.103, 0.575]
Block 3 Level 1	49.6 (27.2)	34.9 (18.1)	**t-test = 2.61, p-value = 0.01**	d = 0.649, CI d [0.154, 1.143]; r = 0.326, CI r [0.090, 0.562]
Block 3 Level 2	43.7 (19.8)	31.9 (16.9)	**t-test = 2.63, p-value = 0.01**	d = 0.648, CI d [0.150, 1.147]; r = 0.311, CI r [0.074, 0.549]
Block 3 Level 3	41.8 (21.4)	31.1 (18.6)	**t-test = 2.20, p-value = 0.03**	d = 0.535, CI d [0.045, 1.026]; r = 0.282, CI r [0.047, 0.518]
Block 3 Level 4	42.6 (21.5)	29.6 (17.3)	**t-test = 2.72, p-value = 0.01**	d = 0.666, CI d [0.171, 1.161]; r = 0.330, CI r [0.094, 0.566]
Block 3 Level 5	42.7 (18.9)	30.9 (18.5)	**t-test = 2.60, p-value = 0.01**	d = 0.628, CI d [0.134, 1.121]; r = 0.338, CI r [0.102, 0.573]

Block 3 Level 6	41.1 (20.8)	30.9 (17.2)	**t-test = 2.20, p-value = 0.03**	d = 0.543, CI d [0.049, 1.038]; r = 0.283, CI r [0.045, 0.521]

HRV frequency domains

HRV HF

Table S4. HRV frequency domain HRV HF by levels.

**Variable**	**Cluster 0 mean (SD)**	**Cluster 1 mean (SD)**	**Statistics**	**Metrics**
**Block 1 Level 1**	0.042 (0.029)	0.029 (0.028)	**U-test = 766, p-value = 0.039**	RBC = -0.290, CLES = 0.645
Block 1 Level 2	0.044 (0.025)	0.037 (0.025)	U-test = 715, p-value = 0.148	RBC = -0.204, CLES = 0.602
**Block 1 Level 3**	0.047 (0.029)	0.032 (0.026)	**U-test = 762, p-value = 0.023**	RBC = -0.323, CLES = 0.661
**Block 1 Level 4**	0.046 (0.025)	0.029 (0.024)	**U-test = 821, p-value = 0.007**	RBC = -0.382, CLES = 0.691
Block 1 Level 5	0.044 (0.027)	0.034 (0.024)	U-test = 705, p-value = 0.114	RBC = -0.224, CLES = 0.612
Block 1 Level 6	0.042 (0.027)	0.034 (0.025)	U-test = 670, p-value = 0.161	RBC = -0.201, CLES = 0.600
**Block 2 Level 1**	0.051 (0.022)	0.034 (0.023)	**U-test = 838, p-value = 0.003**	RBC = -0.416, CLES = 0.708
Block 2 Level 2	0.043 (0.025)	0.044 (0.030)	U-test = 585, p-value = 0.938	RBC = 0.012, CLES = 0.494
**Block 2 Level 3**	0.042 (0.027)	0.026 (0.019)	**U-test = 754, p-value = 0.012**	RBC = -0.359, CLES = 0.679
Block 2 Level 4	0.043 (0.027)	0.036 (0.029)	U-test = 670, p-value = 0.351	RBC = -0.132, CLES = 0.566
**Block 2 Level 5**	0.049 (0.027)	0.035 (0.029)	**t-test = 1.99, p-value = 0.05**	d = 0.474, CI d [-0.011, 0.958]; r = 0.297, CI r [0.063, 0.532]
Block 2 Level 6	0.041 (0.025)	0.031 (0.022)	t-test = 1.72, p-value = 0.09	d = 0.416, CI d [-0.070, 0.903]; r = 0.213, CI r [-0.023, 0.449]
Block 3 Level 1	0.047 (0.028)	0.044 (0.032)	t-test = 0.33, p-value = 0.74	d = 0.078, CI d [-0.400, 0.556]; r = 0.063, CI r [-0.172, 0.297]
Block 3 Level 2	0.050 (0.033)	0.039 (0.030)	t-test = 1.40, p-value = 0.17	d = 0.336, CI d [-0.145, 0.817]; r = 0.174, CI r [-0.061, 0.408]
**Block 3 Level 3**	0.050 (0.032)	0.036 (0.029)	**t-test = 2.02, p-value = 0.05**	d = 0.487, CI d [0.002, 0.972]; r = 0.244, CI r [0.010, 0.478]

**Block 3 Level 4**	0.045 (0.028)	0.033 (0.024)	**t-test = 1.98, p-value = 0.05**	d = 0.479, CI d [-0.005, 0.964]; r = 0.233, CI r [-0.002, 0.467]
Block 3 Level 5	0.041 (0.025)	0.032 (0.025)	t-test = 1.64, p-value = 0.11	d = 0.391, CI d [-0.091, 0.874]; r = 0.221, CI r [-0.013, 0.456]
Block 3 Level 6	0.045 (0.032)	0.036 (0.030)	t-test = 1.12, p-value = 0.27	d = 0.272, CI d [-0.212, 0.756]; r = 0.159, CI r [-0.077, 0.395]

HRV LF

Table S5. HRV frequency domain HRV LF by levels.

Variable	Cluster 0 mean (SD)	Cluster 1 mean (SD)	Statistics	Metrics
Block 1 Level 1	0.025 (0.020)	0.023 (0.019)	U-test = 640, p-value = 0.585	RBC = -0.077, CLES = 0.539
Block 1 Level 2	0.028 (0.020)	0.032 (0.021)	U-test = 511, p-value = 0.428	RBC = 0.113, CLES = 0.444
Block 1 Level 3	0.031 (0.021)	0.031 (0.021)	U-test = 577, p-value = 0.995	RBC = -0.002, CLES = 0.501
Block 1 Level 4	0.029 (0.018)	0.033 (0.019)	U-test = 520, p-value = 0.377	RBC = 0.125, CLES = 0.438
Block 1 Level 5	0.030 (0.019)	0.032 (0.020)	U-test = 539, p-value = 0.654	RBC = 0.064, CLES = 0.468
Block 1 Level 6	0.030 (0.018)	0.036 (0.019)	U-test = 479, p-value = 0.236	RBC = 0.168, CLES = 0.416
Block 2 Level 1	0.028 (0.019)	0.026 (0.018)	U-test = 655, p-value = 0.452	RBC = -0.106, CLES = 0.553
Block 2 Level 2	0.027 (0.018)	0.034 (0.017)	U-test = 450, p-value = 0.089	RBC = 0.240, CLES = 0.380
Block 2 Level 3	0.029 (0.017)	0.031 (0.020)	U-test = 570, p-value = 0.796	RBC = 0.037, CLES = 0.481
Block 2 Level 4	0.034 (0.019)	0.036 (0.020)	U-test = 567, p-value = 0.768	RBC = 0.042, CLES = 0.479
Block 2 Level 5	0.036 (0.020)	0.039 (0.020)	t-test = -0.53, p-value = 0.60	d = -0.127, CI d = [-0.605, 0.351]; r = -0.036, CI r = [-0.270, 0.198]
Block 2 Level 6	0.035 (0.020)	0.037 (0.020)	t-test = -0.51, p-value = 0.61	d = -0.123, CI d = [-0.601, 0.355]; r = -0.063, CI r = [-0.297, 0.172]
Block 3 Level 1	**0.026 (0.016)**	**0.035 (0.019)**	**t-test = -2.08, p-value = 0.04**	**d = -0.492, CI d = [-0.977, -0.007]; r = -0.220, CI r = [-0.454, 0.014]**
Block 3 Level 2	0.033 (0.020)	0.036 (0.017)	t-test = -0.52, p-value = 0.60	d = -0.127, CI d = [-0.605, 0.352]; r = -0.063, CI r = [-0.297, 0.172]
Block 3 Level 3	0.031 (0.020)	0.034 (0.017)	t-test = -0.57, p-value = 0.57	d = -0.138, CI d = [-0.616, 0.341]; r = -0.084, CI r = [-0.318, 0.151]

Block 3 Level 4	0.034 (0.018)	0.037 (0.017)	t-test = -0.74, p-value = 0.46	d = -0.178, CI d = [-0.656, 0.301]; r = -0.065, CI r = [-0.300, 0.169]
Block 3 Level 5	0.032 (0.018)	0.036 (0.019)	t-test = -0.96, p-value = 0.34	d = -0.229, CI d = [-0.708, 0.251]; r = -0.098, CI r = [-0.332, 0.137]
Block 3 Level 6	0.032 (0.019)	0.036 (0.017)	t-test = -0.85, p-value = 0.40	d = -0.204, CI d = [-0.684, 0.275]; r = -0.099, CI r = [-0.333, 0.135]

HRV LF/HF ratio

Table S6. HRV frequency domain HRV HRV LF/HF ratio by levels.

**Variable**	**Cluster 0 mean (SD)**	**Cluster 1 mean (SD)**	**Statistics**	**Metrics**
Block 1 Level 1	0.75 (0.54)	1.05 (0.68)	U-test = 421, p-value = 0.080	RBC = 0.250, CLES = 0.375
Block 1 Level 2	0.86 (0.91)	1.22 (1.10)	U-test = 421, p-value = 0.082	RBC = 0.248, CLES = 0.376
**Block 1 Level 3**	0.88 (0.82)	1.44 (1.15)	**U-test = 383, p-value = 0.027**	RBC = 0.316, CLES = 0.342
**Block 1 Level 4**	0.76 (0.53)	1.66 (1.31)	**U-test = 325, p-value = 0.003**	RBC = 0.421, CLES = 0.290
Block 1 Level 5	0.76 (0.48)	1.14 (0.84)	U-test = 386, p-value = 0.065	RBC = 0.268, CLES = 0.366
Block 1 Level 6	0.97 (0.79)	1.76 (1.78)	U-test = 404, p-value = 0.051	RBC = 0.279, CLES = 0.361
**Block 2 Level 1**	0.64 (0.52)	0.97 (0.70)	**U-test = 400, p-value = 0.031**	RBC = 0.306, CLES = 0.347
Block 2 Level 2	0.85 (0.82)	1.10 (0.84)	U-test = 431, p-value = 0.076	RBC = 0.252, CLES = 0.374
**Block 2 Level 3**	0.88 (0.74)	1.62 (1.38)	**U-test = 365, p-value = 0.010**	RBC = 0.366, CLES = 0.317
Block 2 Level 4	1.38 (1.99)	1.74 (1.60)	U-test = 476, p-value = 0.222	RBC = 0.174, CLES = 0.413
**Block 2 Level 5**	0.96 (0.79)	1.63 (1.32)	**t-test = -2.56, p-value = 0.01**	d = -0.612, CI d [-1.108, -0.116]; r = -0.239, CI r [-0.477, -0.001]
**Block 2 Level 6**	1.00 (0.56)	1.76 (1.56)	**t-test = -2.70, p-value = 0.01**	d = -0.641, CI d [-1.137, -0.144]; r = -0.176, CI r [-0.414, 0.061]
Block 3 Level 1	0.76 (0.55)	1.06 (0.83)	t-test = -1.76, p-value = 0.08	d = -0.421, CI d [-0.911, 0.069]; r = -0.170, CI r [-0.408, 0.067]
**Block 3 Level 2**	1.04 (1.19)	1.72 (1.66)	**t-test = -1.97, p-value = 0.05**	d = -0.463, CI d [-0.952, 0.025]; r = -0.236, CI r [-0.472, -0.000]

**Block 3 Level 3**	0.96 (0.87)	1.92 (2.23)	**t-test = -2.40, p-value = 0.02**	d = -0.560, CI d [-1.050, -0.069]; r = -0.246, CI r [-0.482, -0.010]
**Block 3 Level 4**	1.17 (1.05)	1.86 (1.47)	**t-test = -2.26, p-value = 0.03**	d = -0.531, CI d [-1.017, -0.045]; r = -0.247, CI r [-0.481, -0.013]
**Block 3 Level 5**	1.14 (1.06)	1.76 (1.31)	**t-test = -2.17, p-value = 0.03**	d = -0.518, CI d [-1.007, -0.029]; r = -0.246, CI r [-0.482, -0.010]
**Block 3 Level 6**	0.76 (0.61)	1.57 (1.16)	**t-test = -3.65, p-value = 0.0006**	d = -0.844, CI d [-1.356, -0.333]; r = -0.364, CI r [-0.603, -0.124]

Oculomotor Metrics

Normalized pupil size max

Table S7. Oculomotor metrics, normalized pupil size max by levels.

**Variable**	**Cluster 0 mean (SD)**	**Cluster 1 mean (SD)**	**Statistics**	**Metrics**
**Block 1 Level 1**	1.30 (1.05)	-0.02 (1.02)	**U-test = 976, p-value = 0.000**	RBC = -0.599, CLES = 0.799
**Block 1 Level 2**	0.13 (0.88)	-0.43 (0.97)	**U-test = 876, p-value = 0.002**	RBC = -0.435, CLES = 0.717
Block 1 Level 3	0.23 (0.79)	0.09 (0.76)	U-test = 688, p-value = 0.365	RBC = -0.127, CLES = 0.563
Block 1 Level 4	0.38 (0.97)	0.57 (0.79)	U-test = 523, p-value = 0.306	RBC = 0.143, CLES = 0.428
Block 1 Level 5	0.33 (0.85)	0.52 (0.69)	U-test = 480, p-value = 0.173	RBC = 0.192, CLES = 0.404
Block 1 Level 6	0.09 (0.70)	0.47 (0.80)	U-test = 456, p-value = 0.070	RBC = 0.253, CLES = 0.373
**Block 2 Level 1**	0.03 (1.09)	-0.79 (0.93)	**t-test = 3.35, p-value = 0.001**	d = 0.809, CI d [0.312, 1.306]; r = 0.406, CI r [0.171, 0.640]
Block 2 Level 2	-0.28 (1.00)	-0.69 (0.80)	U-test = 767, p-value = 0.066	RBC = -0.256, CLES = 0.628
**Block 2 Level 3**	0.09 (0.87)	0.71 (0.82)	**U-test = 350, p-value = 0.002**	RBC = 0.427, CLES = 0.287
**Block 2 Level 4**	-0.40 (0.90)	0.46 (0.92)	**U-test = 296, p-value = 0.000**	RBC = 0.515, CLES = 0.242
**Block 2 Level 5**	-0.16 (0.87)	0.29 (0.92)	**U-test = 395, p-value = 0.011**	RBC = 0.353, CLES = 0.324
**Block 2 Level 6**	-0.21 (0.74)	0.39 (0.92)	**U-test = 364, p-value = 0.004**	RBC = 0.404, CLES = 0.298

**Block 3 Level 1**	0.06 (1.23)	-0.93 (0.83)	**t-test = 3.87, p-value = 0.000**	d = 0.947, CI d [0.443, 1.451]; r = 0.411, CI r [0.177, 0.646]
Block 3 Level 2	-0.93 (0.82)	-1.08 (0.73)	t-test = 0.81, p-value = 0.42	d = 0.198, CI d [-0.285, 0.681]; r = 0.107, CI r [-0.129, 0.343]
Block 3 Level 3	-0.51 (0.64)	-0.32 (0.76)	t-test = -1.09, p-value = 0.28	d = -0.258, CI d [-0.738, 0.221]; r = -0.115, CI r [-0.349, 0.120]
Block 3 Level 4	-0.23 (0.92)	0.09 (0.76)	U-test = 454, p-value = 0.066	RBC = 0.256, CLES = 0.372
Block 3 Level 5	-0.10 (0.96)	0.28 (0.93)	t-test = -1.72, p-value = 0.09	d = -0.412, CI d [-0.895, 0.071]; r = -0.217, CI r [-0.452, 0.017]
Block 3 Level 6	0.11 (0.90)	0.29 (0.67)	U-test = 528, p-value = 0.335	RBC = 0.135, CLES = 0.432

Normalized pupil size mean

Table S8. Oculomotor metrics, normalized pupil size mean by levels.

**Variable**	**Cluster 0 mean (SD)**	**Cluster 1 mean (SD)**	**Statistics**	**Metrics**
**Block 1 Level 1**	1.45 (0.87)	-0.46 (0.81)	**U-test = 1141, p-value = p = 5.0e-10**	r = -0.869, CI [0.934]
**Block 1 Level 2**	0.22 (0.86)	-0.66 (0.62)	**U-test = 992, p-value = 7.0e-6**	r = -0.625, CI [0.812]
Block 1 Level 3	0.26 (0.87)	0.19 (0.73)	U-test = 675, p-value = 0.45	r = -0.106, CI [0.553]
Block 1 Level 4	0.48 (0.81)	0.73 (0.61)	t-test = -1.46, p-value = 0.15	d = -0.359, CI d [-0.845, 0.127]; r = -0.129, CI r [-0.365, 0.107]
**Block 1 Level 5**	0.49 (0.91)	0.96 (0.60)	**t-test = -2.51, p-value = 0.01**	d = -0.615, CI d [-1.104, -0.127]; r = -0.264, CI r [-0.498, -0.029]
**Block 1 Level 6**	0.47 (0.89)	1.01 (0.69)	**U-test = 360, p-value = 0.003**	RBC = 0.410, CLES = 0.295
**Block 2 Level 1**	-0.08 (0.83)	-1.00 (0.66)	**t-test = 5.10, p-value = 4.0e-6**	d = 1.237, CI d [0.716, 1.759]; r = 0.538, CI r [0.304, 0.772]
Block 2 Level 2	-0.86 (0.74)	-1.04 (0.49)	U-test = 688, p-value = 0.36	r = -0.127, CI [0.563]
**Block 2 Level 3**	0.06 (0.71)	0.70 (0.60)	**U-test = 277, p-value = 9.0e-5**	r = 0.546, CI [0.227]
**Block 2 Level 4**	-0.53 (0.79)	0.47 (0.57)	**U-test = 176, p-value = 3.0e-7**	r = 0.712, CI [0.144]
**Block 2 Level 5**	-0.25 (0.79)	0.61 (0.63)	**U-test = 235, p-value = 1.0e-5**	r = 0.615, CI [0.192]
**Block 2 Level 6**	-0.05 (0.84)	0.52 (0.55)	**U-test = 312, p-value = 5.0e-4**	r = 0.489, CI [0.256]

**Block 3 Level 1**	-0.25 (0.91)	-1.33 (0.63)	**t-test = 5.66, p-value = 6.0e-7**	d = 1.401, CI d [0.864, 1.938]; r = 0.574, CI r [0.338, 0.810]
Block 3 Level 2	-1.09 (0.63)	-1.37 (0.50)	t-test = 1.98, p-value = 0.05	d = 0.485, CI d [-0.004, 0.974]; r = 0.217, CI r [-0.019, 0.453]
Block 3 Level 3	-0.54 (0.66)	-0.47 (0.65)	U-test = 586, p-value = 0.77	r = 0.040, CI [0.480]
**Block 3 Level 4**	-0.20 (0.82)	0.30 (0.61)	**U-test = 323, p-value = 7.0e-4**	r = 0.471, CI [0.265]
Block 3 Level 5	0.02 (0.95)	0.35 (0.67)	U-test = 458, p-value = 0.071	r = 0.250, CI [0.375]
Block 3 Level 6	0.27 (0.96)	0.48 (0.73)	U-test = 497, p-value = 0.18	r = 0.186, CI [0.407]

Normalized pupil size min

Table S9. Oculomotor metrics, normalized pupil size minimum by levels.

**Variable**	**Cluster 0 mean (SD)**	**Cluster 1 mean (SD)**	**Statistics**	**Metrics**
**Block 1 Level 1**	1.02 (0.87)	-0.25 (0.82)	**U-test = 1047, p-value = 3.0e-7**	RBC = 0.857, CLES = 0.715
Block 1 Level 2	0.10 (1.13)	-0.33 (0.78)	t-test = 1.86, p-value = 0.068	d = 0.455, CI d [-0.029, 0.939]; r = 0.309, CI r [0.074, 0.543]
Block 1 Level 3	0.40 (0.66)	0.16 (0.81)	U-test = 691, p-value = 0.236	RBC = 0.584, CLES = 0.167
**Block 1 Level 4**	0.00 (0.91)	0.58 (0.71)	**t-test = -2.93, p-value = 0.005**	d = -0.720, CI d [-1.217, -0.223]; r = -0.343, CI r [-0.579, -0.107]
Block 1 Level 5	0.47 (0.93)	0.64 (0.90)	t-test = -0.78, p-value = 0.436	d = -0.188, CI d [-0.667, 0.291]; r = -0.131, CI r [-0.366, 0.103]
**Block 1 Level 6**	0.14 (1.00)	0.77 (0.89)	**t-test = -2.79, p-value = 0.007**	d = -0.672, CI d [-1.163, -0.181]; r = -0.330, CI r [-0.564, -0.095]
**Block 2 Level 1**	0.09 (0.79)	-0.79 (0.71)	**U-test = 949, p-value = 7.0e-5**	RBC = 0.777, CLES = 0.554
Block 2 Level 2	-0.53 (0.74)	-0.61 (0.74)	t-test = 0.44, p-value = 0.661	d = 0.106, CI d [-0.376, 0.588]; r = 0.007, CI r [-0.229, 0.243]
Block 2 Level 3	0.18 (0.64)	0.43 (0.80)	t-test = -1.44, p-value = 0.155	d = -0.345, CI d [-0.833, 0.144]; r = -0.195, CI r [-0.433, 0.042]
**Block 2 Level 4**	-0.46 (0.81)	0.31 (1.00)	**U-test = 281, p-value = 1.1e-4**	RBC = 0.230, CLES = 0.540
**Block 2 Level 5**	-0.21 (0.82)	0.59 (0.91)	**U-test = 291, p-value = 3.0e-4**	RBC = 0.246, CLES = 0.508
Block 2 Level 6	0.03 (0.98)	0.25 (0.87)	U-test = 497, p-value = 0.184	RBC = 0.407, CLES = 0.186

**Block 3 Level 1**	0.20 (0.95)	-0.91 (0.76)	**U-test = 996, p-value = 5.9e-6**	RBC = 0.816, CLES = 0.631
**Block 3 Level 2**	-0.50 (0.96)	-0.97 (0.97)	**U-test = 784, p-value = 0.042**	RBC = 0.642, CLES = 0.284
Block 3 Level 3	-0.13 (0.78)	-0.28 (0.82)	U-test = 662, p-value = 0.548	RBC = 0.542, CLES = 0.084
**Block 3 Level 4**	-0.24 (0.89)	0.16 (0.95)	**U-test = 400, p-value = 0.021**	RBC = 0.338, CLES = 0.324
Block 3 Level 5	0.04 (1.02)	0.30 (0.87)	U-test = 516, p-value = 0.269	RBC = 0.423, CLES = 0.155
Block 3 Level 6	0.05 (0.94)	0.04 (0.91)	U-test = 601, p-value = 0.919	RBC = 0.508, CLES = -0.015

Saccade count

Table S10. Oculomotor metrics, saccade count by levels.

Variable	Cluster 0 mean (SD)	Cluster 1 mean (SD)	Statistics	Metrics
Block 1 Level 1	90 (34)	103 (27)	t-test = -1.63, p-value = 0.108	d = -0.397, CI d [-0.882, 0.089]; r = -0.212, CI r [-0.448, 0.024]
**Block 1 Level 2**	93 (40)	118 (29)	**U-test = 371.5, p-value = 0.008**	RBC = 0.375, CLES = 0.313
**Block 1 Level 3**	112 (43)	147 (40)	**U-test = 303.5, p-value = 0.0003**	RBC = 0.503, CLES = 0.249
**Block 1 Level 4**	123 (46)	161 (41)	**U-test = 314, p-value = 0.0005**	RBC = 0.486, CLES = 0.257
**Block 1 Level 5**	129 (43)	168 (41)	**U-test = 303, p-value = 0.0005**	RBC = 0.490, CLES = 0.255
**Block 1 Level 6**	131 (40)	163 (38)	**t-test = -3.41, p-value = 0.001**	d = -0.825, CI d [-1.326, -0.324]; r = -0.390, CI r [-0.626, -0.154]
**Block 2 Level 1**	80 (32)	99 (24)	**U-test = 390.5, p-value = 0.015**	RBC = 0.343, CLES = 0.329
**Block 2 Level 2**	80 (36)	112 (27)	**U-test = 298, p-value = 0.0002**	RBC = 0.512, CLES = 0.244
**Block 2 Level 3**	115 (35)	147 (26)	**t-test = -4.38, p-value = 0.00005**	d = -1.072, CI d [-1.590, -0.554]; r = -0.476, CI r [-0.714, -0.238]
**Block 2 Level 4**	121 (37)	158 (36)	**U-test = 258.5, p-value = 0.00004**	RBC = 0.577, CLES = 0.212
**Block 2 Level 5**	130 (35)	169 (41)	**t-test = -4.36, p-value = 0.000005**	d = -1.033, CI d [-1.542, -0.524]; r = -0.456, CI r [-0.690, -0.221]
**Block 2 Level 6**	136 (34)	168 (40)	**t-test = -3.56, p-value = 0.001**	d = -0.844, CI d [-1.343, -0.346]; r = -0.395, CI r [-0.629, -0.161]

**Block 3 Level 1**	77 (32)	97 (22)	**U-test = 401.5, p-value = 0.014**	RBC = 0.342, CLES = 0.329
**Block 3 Level 2**	85 (35)	113 (24)	**U-test = 315.5, p-value = 0.001**	RBC = 0.483, CLES = 0.258
**Block 3 Level 3**	105 (38)	137 (30)	**U-test = 274.5, p-value = 0.0001**	RBC = 0.550, CLES = 0.225
**Block 3 Level 4**	121 (42)	153 (40)	**t-test = -3.20, p-value = 0.002**	d = -0.768, CI d [-1.264, -0.273]; r = -0.393, CI r [-0.627, -0.159]
**Block 3 Level 5**	131 (36)	161 (36)	**U-test = 306.5, p-value = 0.001**	RBC = 0.484, CLES = 0.258
Block 3 Level 6	126 (36)	142 (29)	U-test = 433.5, p-value = 0.055	RBC = 0.270, CLES = 0.365

Saccade velocity mean

Table S11. Oculomotor metrics, saccade velocity mean by levels.

Variable	Cluster 0 mean (SD)	Cluster 1 mean (SD)	Statistics	Metrics
Block 1 Level 1	**102.3 (29.3)**	103.1 (24.6)	t-test = -0.13, p-value = 0.89	d = -0.032, CI d [-0.510, 0.446]; r = -0.089, CI r [-0.323, 0.146]
Block 1 Level 2	99.3 (25.0)	103.7 (25.8)	U-test = 519, p-value = 0.284	RBC = 0.150, CLES = 0.425
Block 1 Level 3	93.6 (21.6)	100.9 (24.3)	U-test = 467, p-value = 0.092	RBC = 0.235, CLES = 0.382
**Block 1 Level 4**	91.6 (18.2)	100.2 (23.6)	**U-test = 443, p-value = 0.049**	RBC = 0.274, CLES = 0.363
Block 1 Level 5	91.5 (20.1)	98.3 (23.1)	U-test = 483.5, p-value = 0.137	RBC = 0.208, CLES = 0.396
Block 1 Level 6	91.5 (19.2)	96.2 (23.0)	t-test = -0.94, p-value = 0.35	d = -0.224, CI d [-0.707, 0.258]; r = -0.159, CI r [-0.395, 0.077]
Block 2 Level 1	94.0 (22.3)	102.0 (24.6)	t-test = -1.42, p-value = 0.16	d = -0.340, CI d [-0.825, 0.145]; r = -0.181, CI r [-0.417, 0.055]
Block 2 Level 2	95.2 (25.3)	102.6 (22.7)	t-test = -1.29, p-value = 0.20	d = -0.311, CI d [-0.792, 0.169]; r = -0.186, CI r [-0.421, 0.048]
Block 2 Level 3	**99.5 (22.6)**	**107.7 (25.3)**	t-test = -1.43, p-value = 0.16	d = -0.340, CI d [-0.821, 0.141]; r = -0.184, CI r [-0.418, 0.051]
Block 2 Level 4	94.1 (22.3)	101.3 (22.7)	t-test = -1.35, p-value = 0.18	d = -0.323, CI d [-0.804, 0.158]; r = -0.214, CI r [-0.449, 0.020]
Block 2 Level 5	90.4 (20.9)	100.5 (24.0)	t-test = -1.88, p-value = 0.06	d = -0.446, CI d [-0.929, 0.038]; r = -0.250, CI r [-0.484, -0.015]
Block 2 Level 6	91.5 (20.2)	100.3 (24.1)	U-test = 449, p-value = 0.058	RBC = 0.265, CLES = 0.368

Block 3 Level 1	93.3 (24.5)	102.6 (23.5)	t-test = -1.60, p-value = 0.11	d = -0.388, CI d [-0.874, 0.098]; r = -0.190, CI r [-0.426, 0.046]
Block 3 Level 2	94.3 (23.8)	102.6 (20.1)	U-test = 453.5, p-value = 0.06	RBC = 0.257, CLES = 0.371
Block 3 Level 3	93.1 (24.5)	101.7 (20.3)	t-test = -1.60, p-value = 0.11	d = -0.388, CI d [-0.870, 0.094]; r = -0.233, CI r [-0.467, 0.002]
Block 3 Level 4	92.5 (21.4)	101.3 (21.1)	t-test = -1.74, p-value = 0.09	d = -0.416, CI d [-0.899, 0.067]; r = -0.251, CI r [-0.485, -0.017]
Block 3 Level 5	92.9 (18.1)	97.3 (20.5)	U-test = 503, p-value = 0.208	RBC = 0.176, CLES = 0.412
Block 3 Level 6	91.7 (19.0)	98.1 (21.8)	U-test = 482, p-value = 0.132	RBC = 0.210, CLES = 0.395

Saccade duration sum

Table S12. Oculomotor metrics, saccade duration sum by levels.

**Variable**	**Cluster 0 mean (SD)**	**Cluster 1 mean (SD)**	**Statistics**	**Metrics**
Block 1 Level 1	6550 (3992)	8093 (3477)	t-test = -1.71, p-value = 0.093	d = -0.414, CI d [-0.900, 0.073]; r = -0.227, CI r [-0.463, 0.009]
Block 1 Level 2	7531 (4406)	9497 (4246)	t-test = -1.88, p-value = 0.064	d = -0.455, CI d [-0.942, 0.032]; r = -0.226, CI r [-0.462, 0.010]
Block 1 Level 3	8507 (4607)	10243 (3983)	t-test = -1.67, p-value = 0.100	d = -0.405, CI d [-0.890, 0.081]; r = -0.204, CI r [-0.440, 0.032]
Block 1 Level 4	9182 (5108)	10892 (4139)	t-test = -1.52, p-value = 0.134	d = -0.370, CI d [-0.855, 0.116]; r = -0.214, CI r [-0.450, 0.022]
**Block 1 Level 5**	8907 (4662)	11570 (4810)	**t-test = -2.33, p-value = 0.023**	d = -0.562, CI d [-1.052, -0.072]; r = -0.272, CI r [-0.508, -0.036]
**Block 1 Level 6**	8939 (4148)	11518 (4807)	**t-test = -2.39, p-value = 0.020**	d = -0.572, CI d [-1.063, -0.082]; r = -0.262, CI r [-0.498, -0.026]
**Block 2 Level 1**	6157 (4003)	8936 (4170)	**t-test = -2.82, p-value = 0.006**	d = -0.679, CI d [-1.174, -0.185]; r = -0.334, CI r [-0.570, -0.098]
**Block 2 Level 2**	7124 (4586)	9787 (3935)	**t-test = -2.59, p-value = 0.012**	d = -0.626, CI d [-1.115, -0.137]; r = -0.321, CI r [-0.556, -0.087]
**Block 2 Level 3**	8859 (4066)	12542 (4941)	**t-test = -3.42, p-value = 0.001**	d = -0.809, CI [-1.306, -0.313]; r = -0.373, CI r [-0.608, -0.139]
**Block 2 Level 4**	9705 (4801)	12575 (4895)	**U-test = 439.5, p-value = 0.045**	RBC = 0.280, CLES = 0.360
**Block 2 Level 5**	9285 (4524)	12583 (4513)	**U-test = 358, p-value = 0.005**	RBC = 0.397, CLES = 0.301
**Block 2 Level 6**	9291 (4315)	12501 (4598)	**t-test = -3.01, p-value = 0.004**	d = -0.719, CI [-1.212, -0.226]; r = -0.352, CI r [-0.587, -0.118]

**Block 3 Level 1**	6300 (3671)	9073 (3614)	**U-test = 353, p-value = 0.002**	RBC = 0.422, CLES = 0.289
**Block 3 Level 2**	6890 (4078)	10226 (3588)	**t-test = -3.61, p-value = 0.001**	d = -0.872, CI [-1.372, -0.372]; r = -0.416, CI r [-0.650, -0.181]
**Block 3 Level 3**	7890 (4129)	11119 (4343)	**t-test = -3.19, p-value = 0.002**	d = -0.761, CI [-1.256, -0.266]; r = -0.344, CI r [-0.578, -0.110]
**Block 3 Level 4**	8706 (4339)	11796 (4476)	**t-test = -2.93, p-value = 0.005**	d = -0.700, CI [-1.193, -0.208]; r = -0.326, CI r [-0.560, -0.091]
**Block 3 Level 5**	9793 (4144)	12540 (5101)	**U-test = 414, p-value = 0.021**	RBC = 0.322, CLES = 0.339
Block 3 Level 6	8864 (4355)	10329 (4039)	U-test = 486, p-value = 0.145	RBC = 0.204, CLES = 0.398

Saccade amplitude mean

Table S13. Oculomotor metrics, saccade amplitude mean by levels.

Variable	Cluster 0 mean (SD)	Cluster 1 mean (SD)	Statistics	Metrics
Block 1 Level 1	1.64 (0.33)	1.78 (0.36)	t-test = -1.72, p-value = 0.09	d = -0.416, CI [-0.906, 0.074]; r = -0.218, CI r [-0.455, 0.020]
Block 1 Level 2	1.79 (0.59)	1.90 (0.44)	t-test = -0.82, p-value = 0.41	d = -0.202, CI [-0.686, 0.281]; r = -0.120, CI r [-0.355, 0.116]
Block 1 Level 3	1.76 (0.58)	1.91 (0.51)	t-test = -1.12, p-value = 0.27	d = -0.271, CI [-0.754, 0.213]; r = -0.161, CI r [-0.396, 0.075]
Block 1 Level 4	1.77 (0.67)	1.90 (0.54)	t-test = -0.90, p-value = 0.37	d = -0.218, CI [-0.697, 0.261]; r = -0.188, CI r [-0.422, 0.047]
Block 1 Level 5	1.65 (0.47)	1.85 (0.56)	t-test = -1.58, p-value = 0.12	d = -0.377, CI [-0.863, 0.109]; r = -0.164, CI r = [-0.400, 0.072]
Block 1 Level 6	1.69 (0.55)	1.80 (0.57)	t-test = -0.80, p-value = 0.43	d = -0.193, CI [-0.676, 0.290]; r = -0.109, CI r [-0.345, 0.127]
Block 2 Level 1	1.64 (0.51)	1.80 (0.60)	t-test = -1.20, p-value = 0.23	d = -0.288, CI [-0.772, 0.195]; r = -0.112, CI r = [-0.348, 0.124]
Block 2 Level 2	1.76 (0.60)	1.81 (0.38)	t-test = -0.41, p-value = 0.68	d = -0.101, CI [-0.585, 0.384]; r = -0.150, CI r [-0.387, 0.088]
Block 2 Level 3	1.88 (0.50)	2.03 (0.47)	t-test = -1.31, p-value = 0.20	d = -0.319, CI [-0.807, 0.170]; r = -0.170, CI r [-0.408, 0.068]
Block 2 Level 4	1.73 (0.58)	1.94 (0.61)	t-test = -1.50, p-value = 0.14	d = -0.360, CI [-0.845, 0.126]; r = -0.220, CI r [-0.456, 0.016]
Block 2 Level 5	1.65 (0.43)	1.97 (0.57)	**t-test = -2.64, p-value = 0.01**	d = -0.623, CI [-1.116, -0.130]; r = -0.271, CI r [-0.507, -0.035]
Block 2 Level 6	1.79 (0.51)	1.85 (0.59)	t-test = -0.50, p-value = 0.62	d = -0.119, CI [-0.601, 0.363]; r = -0.047, CI r [-0.283, 0.189]

Block 3 Level 1	1.63 (0.57)	1.76 (0.52)	t-test = -0.97, p-value = 0.34	d = -0.234, CI [-0.716, 0.249]; r = -0.150, CI r [-0.386, 0.086]
Block 3 Level 2	1.69 (0.61)	2.02 (0.61)	**t-test = -2.23, p-value = 0.03**	d = -0.539, CI [-1.030, -0.049]; r = -0.365, CI r = [-0.601, -0.129]
Block 3 Level 3	1.66 (0.51)	1.86 (0.48)	t-test = -1.68, p-value = 0.10	d = -0.405, CI [-0.891, 0.081]; r = -0.246, CI r [-0.482, -0.010]
Block 3 Level 4	1.63 (0.41)	1.85 (0.43)	**t-test = -2.15, p-value = 0.04**	d = -0.521, CI [-1.014, -0.027]; r = -0.270, CI r [-0.507, -0.032]
Block 3 Level 5	1.67 (0.44)	1.77 (0.37)	t-test = -1.02, p-value = 0.31	d = -0.250, CI [-0.737, 0.237]; r = -0.126, CI r [-0.364, 0.112]
Block 3 Level 6	1.61 (0.43)	1.69 (0.51)	t-test = -0.70, p-value = 0.49	d = -0.169, CI [-0.655, 0.317]; r = -0.037, CI r [-0.274, 0.201]

Fixation duration mean

Table S14. Oculomotor metrics, fixation duration mean by levels.

**Variable**	**Cluster 0 mean (SD)**	**Cluster 1 mean (SD)**	**Statistics**	**Metrics**
**Block 1 Level 1**	520 (168)	439 (113)	**t-test = 2.27, p-value = 0.03**	d = 0.571, CI d [0.075, 1.066]; r = 0.216, CI r [-0.022, 0.453]
**Block 1 Level 2**	532 (222)	392 (92)	**t-test = 3.34, p-value = 0.002**	d = 0.850, CI d [0.347, 1.353]; r = 0.306, CI r [0.070, 0.542]
**Block 1 Level 3**	439 (133)	355 (95)	**t-test = 2.93, p-value = 0.005**	d = 0.734, CI d [0.232, 1.237]; r = 0.287, CI r [0.050, 0.525]
**Block 1 Level 4**	411 (130)	337 (88)	**t-test = 2.69, p-value = 0.01**	d = 0.678, CI d [0.178, 1.177]; r = 0.256, CI r [0.018, 0.494]
**Block 1 Level 5**	418 (132)	322 (80)	**t-test = 3.60, p-value = 0.0007**	d = 0.885, CI d [0.385, 1.386]; r = 0.334, CI r [0.100, 0.568]
**Block 1 Level 6**	412 (114)	333 (85)	**t-test = 3.25, p-value = 0.002**	d = 0.791, CI d [0.295, 1.287]; r = 0.320, CI r [0.086, 0.554]
**Block 2 Level 1**	584 (226)	442 (119)	**t-test = 3.21, p-value = 0.002**	d = 0.807, CI d [0.306, 1.308]; r = 0.323, CI r [0.087, 0.559]
**Block 2 Level 2**	569 (210)	407 (104)	**t-test = 3.91, p-value = 0.0003**	d = 1.004, CI d [0.489, 1.520]; r = 0.404, CI r [0.166, 0.642]
**Block 2 Level 3**	445 (129)	335 (73)	**t-test = 4.26, p-value = 0.0001**	**d = 1.069, CI d [0.554, 1.584]; r = 0.433, CI r [0.197, 0.669]**
**Block 2 Level 4**	426 (116)	332 (75)	**t-test = 3.91, p-value = 0.0003**	d = 0.973, CI d [0.464, 1.483]; r = 0.378, CI r [0.142, 0.614]
**Block 2 Level 5**	404 (107)	317 (70)	**t-test = 3.91, p-value = 0.0003**	d = 0.972, CI d [0.463, 1.481]; r = 0.365, CI r [0.129, 0.601]

**Block 2 Level 6**	381 (84)	321 (70)	**t-test = 3.15, p-value = 0.0003**	d = 0.781, CI d [0.277, 1.285]; r = 0.314, CI r [0.077, 0.552]
**Block 3 Level 1**	551 (174)	443 (109)	**t-test = 2.96, p-value = 0.005**	d = 0.762, CI d [0.254, 1.270]; r = 0.317, CI r [0.078, 0.557]
**Block 3 Level 2**	528 (167)	397 (83)	**t-test = 3.95, p-value = 0.0003**	d = 1.016, CI d [0.500, 1.533]; r = 0.390, CI r [0.153, 0.628]
**Block 3 Level 3**	455 (123)	357 (83)	**t-test = 3.79, p-value = 0.0004**	d = 0.940, CI d [0.433, 1.448]; r = 0.413, CI r [0.177, 0.649]
**Block 3 Level 4**	426 (117)	338 (70)	**t-test = 3.77, p-value = 0.0004**	d = 0.928, CI d [0.426, 1.431]; r = 0.385, CI r [0.150, 0.619]
**Block 3 Level 5**	389 (98)	323 (70)	**t-test = 3.18, p-value = 0.002**	d = 0.787, CI d [0.287, 1.287]; r = 0.323, CI r [0.087, 0.559]
**Block 3 Level 6**	375 (96)	329 (71)	**t-test = 2.23, p-value = 0.03**	d = 0.550, CI d [0.059, 1.041]; r = 0.193, CI r [-0.043, 0.429]

Fixation count

Table S15. Oculomotor metrics, fixation count by levels.

Variable	Cluster 0 mean (SD)	Cluster 1 mean (SD)	Statistics	Metrics
Block 1 Level 1	104 (33)	117 (26)	t-test = -1.76, p-value = 0.08	d = -0.428, CI d [-0.915, 0.058]; r = -0.223, CI r [-0.459, 0.013]
**Block 1 Level 2**	107 (39)	131 (28)	**U-test = 378.5, p-value = 0.01**	RBC = 0.363, CLES = 0.319
**Block 1 Level 3**	125 (42)	160 (38)	**U-test = 292, p-value = 0.0002**	RBC = 0.522, CLES = 0.239
**Block 1 Level 4**	135 (44)	173 (38)	**U-test = 306, p-value = 0.0003**	RBC = 0.499, CLES = 0.251
**Block 1 Level 5**	141 (42)	180 (39)	**U-test = 285, p-value = 0.0002**	RBC = 0.520, CLES = 0.240
**Block 1 Level 6**	142 (39)	174 (36)	**t-test = -3.50, p-value = 0.001**	d = -0.845, CI d [-1.347, -0.343]; r = -0.398, CI r [-0.634, -0.162]
**Block 2 Level 1**	94 (31)	112 (22)	**U-test = 388.5, p-value = 0.014**	RBC = 0.346, CLES = 0.327
**Block 2 Level 2**	94 (35)	125 (26)	**U-test = 288.5, p-value = 0.0001**	RBC = 0.527, CLES = 0.236
**Block 2 Level 3**	126 (34)	159 (24)	**t-test = -4.58, p-value = 0.00002**	d = -1.123, CI d [-1.644, -0.602]; r = -0.496, CI r [-0.733, -0.258]
**Block 2 Level 4**	131 (36)	169 (34)	**U-test = 248.5, p-value = 0.00002**	RBC = 0.593, CLES = 0.204
**Block 2 Level 5**	140 (35)	180 (40)	**U-test = 275.5, p-value = 0.00008**	RBC = 0.549, CLES = 0.226

**Block 2 Level 6**	147 (34)	179 (40)	**t-test = -3.56, p-value = 0.001**	d = -0.846, CI d [-1.345, -0.347]; r = -0.394, CI r [-0.629, -0.160]
**Block 3 Level 1**	92 (31)	110 (21)	**U-test = 416, p-value = 0.02**	RBC = 0.319, CLES = 0.341
**Block 3 Level 2**	99 (34)	125 (23)	**U-test = 324, p-value = 0.001**	RBC = 0.469, CLES = 0.265
**Block 3 Level 3**	118 (35)	149 (29)	**U-test = 282.5, p-value = 0.0001**	RBC = 0.537, CLES = 0.231
**Block 3 Level 4**	134 (42)	165 (39)	**t-test = -3.20, p-value = 0.002**	d = -0.771, CI d [-1.266, -0.276]; r = -0.421, CI r [-0.655, -0.187]
**Block 3 Level 5**	142 (36)	173 (34)	**U-test = 306.5, p-value = 0.001**	RBC = 0.484, CLES = 0.258
Block 3 Level 6	136 (36)	152 (28)	U-test = 429, p-value = 0.048	RBC = 0.278, CLES = 0.361

Blink count

Table S16. Oculomotor metrics, blink count by levels.

**Variable**	**Cluster 0 mean (SD)**	**Cluster 1 mean (SD)**	**Statistics**	**Metrics**
Block 1 Level 1	90 (34)	102(27)	t-test = -1.63, p-value = 0.11	d = -0.397, CI [-0.882, 0.089]; r = -0.212, CI r [-0.448, 0.024]
**Block 1 Level 2**	93 (40)	117 (28)	**U-test = 371.5, p-value = 0.008**	RBC = 0.375, CLES = 0.313
**Block 1 Level 3**	112 (43)	147 (39)	**U-test = 303.5, p-value = 0.0003**	RBC = 0.503, CLES = 0.249
**Block 1 Level 4**	123 (45)	161 (40)	**U-test = 314, p-value = 0.0005**	RBC = 0.486, CLES = 0.257
**Block 1 Level 5**	129 (43)	167 (40)	**U-test = 303, p-value = 0.0005**	RBC = 0.490, CLES = 0.255
**Block 1 Level 6**	130 (40)	162 (38)	**t-test = -3.41, p-value = 0.001**	d = -0.825, CI [-1.326, -0.324]; r = -0.390, CI к = [-0.626, -0.154]
**Block 2 Level 1**	80 (32)	98 (23)	**U-test = 390.5, p-value = 0.015**	RBC = 0.343, CLES = 0.329
**Block 2 Level 2**	80 (36)	112 (27)	**U-test = 298, p-value = 0.0002**	RBC = 0.512, CLES = 0.244
**Block 2 Level 3**	114 (34)	147 (25)	**t-test = -4.38, p-value = 0.00005**	d = -1.072, CI [-1.590, -0.554]; r = -0.476, CI к = [-0.714, -0.238]
**Block 2 Level 4**	120 (36)	158 (35)	**U-test = 258.5, p-value = 0.00004**	RBC = 0.577, CLES = 0.212
**Block 2 Level 5**	130 (35)	169 (41)	**t-test = -4.36, p-value = 0.00005**	d = -1.033, CI [-1.542, -0.524]; r = -0.456, CI к = [-0.690, -0.221]

**Block 2 Level 6**	136 (34)	168 (40)	**t-test = -3.56, p-value = 0.001**	d = -0.844, CI [-1.343, -0.346]; r = -0.395, CI r [-0.629, -0.161]
**Block 3 Level 1**	77 (32)	96 (22)	**U-test = 401.5, p-value = 0.014**	RBC = 0.342, CLES = 0.329
**Block 3 Level 2**	85 (35)	113 (24)	**U-test = 315.5, p-value = 0.001**	RBC = 0.483, CLES = 0.258
**Block 3 Level 3**	105 (37)	137 (30)	**U-test = 274.5, p-value = 0.0001**	RBC = 0.550, CLES = 0.225
**Block 3 Level 4**	121 (41)	153 (40)	**t-test = -3.20, p-value = 0.002**	d = -0.768, CI [-1.264, -0.273]; r = -0.393, CI к = [-0.627, -0.159]
**Block 3 Level 5**	131 (36)	161 (36)	**U-test = 306.5, p-value = 0.001**	RBC = 0.484, CLES = 0.258
Block 3 Level 6	125 (36)	141 (28)	U-test = 433.5, p-value = 0.055	RBC = 0.270, CLES = 0.365

Blink duration

Table S17. Oculomotor metrics, blink duration by levels.

**Variable**	**Cluster 0 mean (SD)**	**Cluster 1 mean (SD)**	**Statistics**	**Metrics**
Block 1 Level 1	3674 (2869)	4457 (2411)	t-test = -1.22, p-value = 0.23	Cohen's d = -0.297, CI = [-0.780, 0.187], r = -0.187, CI = [-0.423, 0.049]
Block 1 Level 2	4496 (3253)	5306 (3163)	t-test = -1.05, p-value = 0.30	Cohen's d = -0.253, CI = [-0.736, 0.230], r = -0.158, CI = [-0.394, 0.078]
Block 1 Level 3	4966 (3482)	5361 (2873)	t-test = -0.51, p-value = 0.61	Cohen's d = -0.124, CI = [-0.606, 0.357], r = -0.078, CI = [-0.314, 0.158]
Block 1 Level 4	5387 (3892)	5561 (2962)	t-test = -0.21, p-value = 0.84	Cohen's d = -0.051, CI = [-0.532, 0.430], r = -0.073, CI = [-0.309, 0.163]
Block 1 Level 5	4969 (3646)	5954 (3432)	t-test = -1.15, p-value = 0.25	Cohen's d = -0.279, CI = [-0.762, 0.205], r = -0.184, CI = [-0.420, 0.052]
Block 1 Level 6	5050 (3389)	6077 (3485)	t-test = -1.24, p-value = 0.22	Cohen's d = -0.299, CI = [-0.782, 0.185], r = -0.164, CI = [-0.400, 0.072]
**Block 2 Level 1**	**3751 (3144)**	**5407 (3077)**	**t-test = -2.21, p-value = 0.03**	**Cohen's d = -0.532, CI = [-1.022, -0.043], r = -0.283, CI = [-0.519, -0.047]**
Block 2 Level 2	4617 (3710)	5776 (2871)	t-test = -1.45, p-value = 0.15	Cohen's d = -0.352, CI = [-0.834, 0.129], r = -0.237, CI = [-0.471, -0.003]
Block 2 Level 3	5286 (3274)	6678 (3257)	t-test = -1.77, p-value = 0.08	Cohen's d = -0.426, CI = [-0.913, 0.060], r = -0.220, CI = [-0.456, 0.016]
Block 2 Level 4	6112 (4012)	6848 (3098)	t-test = -0.85, p-value = 0.40	Cohen's d = -0.207, CI = [-0.689, 0.276], r = -0.098, CI = [-0.334, 0.138]
Block 2 Level 5	5549 (3852)	6839 (3150)	t-test = -1.51, p-value = 0.13	Cohen's d = -0.368, CI = [-0.853, 0.117], r = -0.199, CI = [-0.435, 0.037]

Block 2 Level 6	5357 (3820)	6822 (3472)	t-test = -1.67, p-value = 0.10	Cohen's d = -0.403, CI = [-0.885, 0.080], r = -0.244, CI = [-0.478, -0.010]
**Block 3 Level 1**	**3884 (2727)**	**5705 (2706)**	**t-test = -2.80, p-value = 0.01**	**Cohen's d = -0.670, CI = [-1.161, -0.179], r = -0.333, CI = [-0.567, -0.098]**
**Block 3 Level 2**	**4263 (3044)**	**6236 (2927)**	**t-test = -2.76, p-value = 0.01**	**Cohen's d = -0.662, CI = [-1.152, -0.171], r = -0.326, CI = [-0.560, -0.091]**
**Block 3 Level 3**	**4698 (3310)**	**6370 (3350)**	**t-test = -2.10, p-value = 0.04**	**Cohen's d = -0.502, CI = [-0.987, -0.017], r = -0.240, CI = [-0.474, -0.006]**
Block 3 Level 4	5075 (3544)	6568 (3330)	t-test = -1.81, p-value = 0.07	Cohen's d = -0.435, CI = [-0.918, 0.048], r = -0.220, CI = [-0.454, 0.014]
Block 3 Level 5	5832 (3492)	7131 (3942)	U-test = 493, p-value = 0.169	RBC = 0.192, CLES = 0.404
Block 3 Level 6	5149 (3618)	5632 (3170)	t-test = -0.59, p-value = 0.56	Cohen's d = -0.143, CI = [-0.621, 0.336], r = -0.095, CI = [-0.329, 0.139]

Cognitive metrics

Self-esteem

Table S18. Cognitive metrics, Self-esteem by levels.

Variable	Cluster 0 mean (SD)	Cluster 1 mean (SD)	Statistics	Metrics
Block 1 Level 1	4.3 (0.8)	4.6 (0.7)	t-test = -1.50, p-value = 0.14	d = -0.368, CI d = [-0.858, 0.122]; r = -0.189, CI r [-0.427, 0.049]
**Block 1 Level 2**	**4.1 (0.8)**	**4.5 (0.7)**	**t-test = -2.07, p-value = 0.04**	**d = -0.508, CI d = [-1.004, -0.011]; r = -0.239, CI r [-0.479, 0.000]**
Block 1 Level 3	3.8 (1.0)	4.2 (0.8)	t-test = -1.59, p-value = 0.12	d = -0.386, CI d = [-0.868, 0.096]; r = -0.162, CI r [-0.397, 0.072]
Block 1 Level 4	3.5 (0.8)	3.4 (1.1)	t-test = 0.48, p-value = 0.63	d = 0.112, CI d = [-0.366, 0.591]; r = 0.028, CI r [-0.206, 0.262]
Block 1 Level 5	2.7 (1.0)	2.9 (1.0)	t-test = -1.06, p-value = 0.29	d = -0.255, CI d = [-0.735, 0.225]; r = -0.110, CI r [-0.344, 0.125]
Block 1 Level 6	2.8 (1.0)	2.6 (1.0)	t-test = 0.56, p-value = 0.58	d = 0.133, CI d = [-0.345, 0.612]; r = 0.081, CI r [-0.153, 0.315]

Block 2 Level 1	4.6 (0.6)	4.8 (0.4)	t-test = -1.02, p-value = 0.31	d = -0.252, CI d = [-0.736, 0.232]; r = -0.072, CI r [-0.308, 0.164]
**Block 2 Level 2**	**4.3 (0.8)**	**4.8 (0.4)**	**t-test = -3.06, p-value = 0.004**	**d = -0.769, CI d = [-1.269, -0.270]; r = -0.278, CI r [-0.514, -0.042]**
**Block 2 Level 3**	**2.6 (0.9)**	**3.2 (0.9)**	**t-test = -2.57, p-value = 0.01**	**d = -0.613, CI d = [-1.102, -0.124]; r = -0.278, CI r [-0.513, -0.044]**
Block 2 Level 4	2.5 (1.0)	2.9 (0.9)	t-test = -1.90, p-value = 0.06	d = -0.459, CI d = [-0.943, 0.025]; r = -0.202, CI r [-0.436, 0.032]
Block 2 Level 5	2.4 (0.9)	2.5 (1.0)	t-test = -0.54, p-value = 0.59	d = -0.130, CI d = [-0.608, 0.348]; r = -0.071, CI r [-0.305, 0.163]
Block 2 Level 6	2.2 (0.9)	2.4 (1.0)	t-test = -0.90, p-value = 0.37	d = -0.214, CI d = [-0.693, 0.266]; r = -0.084, CI r [-0.319, 0.150]
Block 3 Level 1	4.8 (0.5)	4.6 (0.6)	t-test = 1.35, p-value = 0.18	d = 0.323, CI d = [-0.161, 0.807]; r = 0.130, CI r [-0.106, 0.366]
Block 3 Level 2	4.5 (0.6)	4.5 (0.7)	t-test = 0.36, p-value = 0.72	d = 0.085, CI d = [-0.393, 0.563]; r = 0.024, CI r [-0.210, 0.258]
Block 3 Level 3	4.4 (0.6)	4.5 (0.7)	t-test = -0.60, p-value = 0.55	d = -0.142, CI d = [-0.621, 0.336]; r = -0.101, CI r [-0.335, 0.134]
Block 3 Level 4	3.9 (0.8)	4.0 (0.8)	t-test = -0.31, p-value = 0.76	d = -0.074, CI d = [-0.552, 0.404]; r = -0.044, CI r [-0.279, 0.190]
Block 3 Level 5	3.4 (0.7)	3.5 (0.6)	t-test = -0.69, p-value = 0.49	d = -0.167, CI d = [-0.649, 0.314]; r = -0.073, CI r [-0.309, 0.163]
Block 3 Level 6	3.5 (0.8)	3.2 (0.8)	t-test = 1.74, p-value = 0.09	d = 0.415, CI d = [-0.068, 0.898]; r = 0.186, CI r [-0.048, 0.421]

Response Time

Table S19. Cognitive metrics, time of response by levels.

Variable	Cluster 0 mean (SD)	Cluster 1 mean (SD)	Statistics	Metrics
Block 1 Level 1	50854 (4077)	50377 (3328)	t-test = 0.53, p-value = 0.60	d = 0.129, CI d = [-0.353, 0.610]; r = 0.049, CI r [-0.187, 0.285]
Block 1 Level 2	51857 (4044)	52628 (4258)	t-test = -0.77, p-value = 0.44	d = -0.186, CI d = [-0.668, 0.296]; r = -0.065, CI r [-0.301, 0.171]
**Block 1 Level 3**	**54222 (5371)**	**57715 (4623)**	**U-test = 358, p-value = 0.003**	**RBC = 0.414, CLES = 0.293**
**Block 1 Level 4**	**55956 (5493)**	**60767 (5240)**	**U-test = 312, p-value = 0.0005**	**RBC = 0.489, CLES = 0.256**
**Block 1 Level 5**	**56706 (5251)**	**61502 (4917)**	**U-test = 309, p-value = 0.0004**	**RBC = 0.494, CLES = 0.253**
**Block 1 Level 6**	**57460 (5365)**	**61511 (4785)**	**U-test = 355, p-value = 0.003**	**RBC = 0.419, CLES = 0.291**
Block 2 Level 1	49408 (3048)	49361 (2403)	t-test = 0.07, p-value = 0.94	d = 0.017, CI d = [-0.464, 0.498]; r = -0.022, CI r [-0.258, 0.214]
Block 2 Level 2	50929 (3607)	51642 (3172)	t-test = -0.87, p-value = 0.39	d = -0.211, CI d = [-0.693, 0.272]; r = -0.126, CI r [-0.362, 0.110]
**Block 2 Level 3**	**55486 (4611)**	**59159 (3951)**	**U-test = 321, p-value = 0.001**	**RBC = 0.474, CLES = 0.263**
**Block 2 Level 4**	**56363 (4882)**	**59925 (4776)**	**U-test = 361, p-value = 0.003**	**RBC = 0.409, CLES = 0.296**
**Block 2 Level 5**	**57336 (4791)**	**61149 (5560)**	**U-test = 370, p-value = 0.005**	**RBC = 0.394, CLES = 0.303**
Block 2 Level 6	59364 (5201)	61227 (4823)	U-test = 476.5, p-value = 0.116	RBC = 0.219, CLES = 0.390

Block 3 Level 1	49071 (2827)	49117 (2158)	U-test = 580, p-value = 0.890	RBC = 0.020, CLES = 0.490
**Block 3 Level 2**	**50258 (2576)**	**51745 (3173)**	**t-test = -2.15, p-value = 0.04**	**d = -0.511, CI d = [-1.000, -0.021]; r = -0.230, CI r [-0.466, 0.006]**
**Block 3 Level 3**	**52792 (4000)**	**56146 (4348)**	**U-test = 340, p-value = 0.002**	**RBC = 0.426, CLES = 0.287**
**Block 3 Level 4**	**55855 (6409)**	**59366 (5923)**	**t-test = -2.37, p-value = 0.02**	**d = -0.570, CI d = [-1.058, -0.083]; r = -0.286, CI r [-0.520, -0.052]**
**Block 3 Level 5**	**57275 (5272)**	**60756 (5984)**	**U-test = 404, p-value = 0.015**	**RBC = 0.338, CLES = 0.331**
Block 3 Level 6	57915 (5851)	59440 (5174)	U-test = 517, p-value = 0.274	RBC = 0.153, CLES = 0.423
**Total_Time**	**1100646 (71544)**	**1148445 (60950)**	**U-test = 354, p-value = 0.003**	**RBC = 0.420, CLES = 0.290**

Mistakes

Table S20. Cognitive metrics, mistakes by levels.

Variable	Cluster 0 mean (SD)	Cluster 1 mean (SD)	Statistics	Metrics
Block 1 Level 1	0.70 (0.95)	0.41 (0.69)	U-test = 637.5, p-value = 0.228	RBC = -0.149, CLES = 0.574
Block 1 Level 2	0.81 (1.14)	0.46 (0.73)	U-test = 643.5, p-value = 0.314	RBC = -0.122, CLES = 0.561
Block 1 Level 3	1.13 (1.22)	0.81 (1.29)	U-test = 673, p-value = 0.110	RBC = -0.213, CLES = 0.606
Block 1 Level 4	2.03 (1.40)	1.89 (1.63)	U-test = 629.5, p-value = 0.482	RBC = -0.098, CLES = 0.549
Block 1 Level 5	3.66 (2.07)	3.41 (1.54)	U-test = 615, p-value = 0.783	RBC = -0.039, CLES = 0.519
Block 1 Level 6	5.03 (2.48)	4.92 (2.06)	U-test = 607.5, p-value = 0.855	RBC = -0.026, CLES = 0.513
Block 2 Level 1	0.25 (0.44)	0.19 (0.40)	U-test = 608, p-value = 0.590	RBC = -0.056, CLES = 0.528
**Block 2 Level 2**	0.61 (0.80)	0.24 (0.49)	**U-test = 707.5, p-value = 0.043**	RBC = -0.234, CLES = 0.617
Block 2 Level 3	2.34 (1.41)	1.86 (1.40)	U-test = 699.5, p-value = 0.187	RBC = -0.182, CLES = 0.591

Block 2 Level 4	2.16 (1.69)	1.97 (1.56)	U-test = 619.5, p-value = 0.588	RBC = -0.076, CLES = 0.538
Block 2 Level 5	3.34 (1.96)	3.51 (1.57)	U-test = 551.5, p-value = 0.625	RBC = 0.068, CLES = 0.466
Block 2 Level 6	5.13 (2.18)	4.46 (2.13)	U-test = 687.5, p-value = 0.247	RBC = -0.161, CLES = 0.581
Block 3 Level 1	0.23 (0.43)	0.30 (0.46)	U-test = 532.5, p-value = 0.514	RBC = 0.071, CLES = 0.464
Block 3 Level 2	0.37 (0.56)	0.32 (0.53)	U-test = 575.5, p-value = 0.755	RBC = -0.037, CLES = 0.518
Block 3 Level 3	0.55 (0.81)	0.62 (0.89)	U-test = 550.5, p-value = 0.749	RBC = 0.040, CLES = 0.480
Block 3 Level 4	1.63 (1.56)	1.19 (1.24)	U-test = 678.5, p-value = 0.284	RBC = -0.146, CLES = 0.573
Block 3 Level 5	3.22 (1.66)	3.00 (1.53)	U-test = 634.5, p-value = 0.605	RBC = -0.072, CLES = 0.536
Block 3 Level 6	3.75 (2.00)	3.68 (1.90)	U-test = 611, p-value = 0.822	RBC = -0.032, CLES = 0.516

Other ANS metrics

RSP Amplitude Mean

Table S21. Other ANS metrics, RSP Amplitude Mean by levels.

Variable	Cluster 0 mean (SD)	Cluster 1 mean (SD)	Statistics	Metrics
Block 1 Level 1	18.8 (9.2)	19.8 (8.7)	U-test = 524, p-value = 0.656	RBC = 0.064, CLES = 0.468
Block 1 Level 2	18.9 (9.9)	19.6 (7.3)	U-test = 506, p-value = 0.644	RBC = 0.067, CLES = 0.466
Block 1 Level 3	18.6 (9.6)	19.3 (7.4)	U-test = 515, p-value = 0.729	RBC = 0.051, CLES = 0.475
Block 1 Level 4	19.7 (11.1)	19.1 (6.7)	U-test = 562.5, p-value = 0.859	RBC = 0.026, CLES = 0.487
Block 1 Level 5	19.3 (10.9)	18.8 (7.1)	U-test = 538, p-value = 0.787	RBC = 0.039, CLES = 0.480
Block 1 Level 6	18.2 (10.0)	19.2 (6.8)	U-test = 464.5, p-value = 0.319	RBC = 0.144, CLES = 0.428
Block 2 Level 1	18.8 (10.8)	19.7 (8.8)	U-test = 562.5, p-value = 0.727	RBC = 0.050, CLES = 0.475

Block 2 Level 2	19.4 (11.0)	18.9 (7.7)	U-test = 586.5, p-value = 0.902	RBC = -0.018, CLES = 0.509
Block 2 Level 3	17.5 (8.9)	18.4 (8.4)	U-test = 534, p-value = 0.768	RBC = 0.043, CLES = 0.478
Block 2 Level 4	18.4 (10.2)	19.2 (8.7)	U-test = 537, p-value = 0.658	RBC = 0.064, CLES = 0.468
Block 2 Level 5	17.7 (10.2)	19.4 (9.1)	t-test = -0.71, p-value = 0.480	d = -0.172, CI d = [-0.655, 0.311]; r = -0.110, CI r [-0.346, 0.126]
Block 2 Level 6	18.6 (11.3)	19.3 (8.0)	t-test = -0.30, p-value = 0.760	d = -0.074, CI d = [-0.555, 0.407]; r = -0.095, CI r [-0.331, 0.141]
Block 3 Level 1	17.2 (9.2)	19.0 (8.1)	t-test = -0.83, p-value = 0.410	d = -0.203, CI d = [-0.689, 0.284]; r = -0.110, CI r [-0.348, 0.127]
Block 3 Level 2	18.0 (10.1)	19.1 (8.4)	t-test = -0.48, p-value = 0.630	d = -0.117, CI d = [-0.599, 0.364]; r = -0.084, CI r [-0.320, 0.152]
Block 3 Level 3	18.3 (9.9)	18.8 (7.3)	t-test = -0.24, p-value = 0.810	d = -0.059, CI d = [-0.540, 0.422]; r = -0.056, CI r [-0.292, 0.180]
Block 3 Level 4	17.2 (9.9)	17.5 (6.3)	U-test = 513, p-value = 0.576	RBC = 0.081, CLES = 0.460
Block 3 Level 5	17.3 (9.5)	18.9 (7.6)	t-test = -0.78, p-value = 0.440	d = -0.192, CI d = [-0.675, 0.291]; r = -0.144, CI r [-0.380, 0.092]
Block 3 Level 6	17.6 (10.3)	18.6 (7.2)	t-test = -0.46, p-value = 0.650	d = -0.114, CI d = [-0.596, 0.368]; r = -0.129, CI r [-0.365, 0.107]

RSP Phase Duration Expiration

Table S22. Other ANS metrics, RSP Phase Duration Expiration by levels.

**Variable**	**Cluster 0 mean (SD)**	**Cluster 1 mean (SD)**	**Statistics**	**Metrics**
Block 1 Level 1	1.90 (0.31)	1.84 (0.34)	U-test = 634.5, p-value = 0.488	RBC = -0.099, CLES = 0.549
Block 1 Level 2	1.88 (0.36)	1.81 (0.35)	U-test = 620, p-value = 0.455	RBC = -0.107, CLES = 0.554
Block 1 Level 3	1.80 (0.28)	1.79 (0.34)	U-test = 544, p-value = 0.990	RBC = -0.003, CLES = 0.501
Block 1 Level 4	1.83 (0.30)	1.82 (0.36)	U-test = 583, p-value = 0.778	RBC = -0.041, CLES = 0.521
Block 1 Level 5	1.94 (0.38)	1.89 (0.36)	U-test = 603, p-value = 0.594	RBC = -0.077, CLES = 0.538
Block 1 Level 6	1.97 (0.40)	1.93 (0.37)	U-test = 585.5, p-value = 0.754	RBC = -0.046, CLES = 0.523
Block 2 Level 1	1.92 (0.38)	1.85 (0.40)	U-test = 656.5, p-value = 0.326	RBC = -0.140, CLES = 0.570
Block 2 Level 2	1.90 (0.35)	1.86 (0.40)	U-test = 632, p-value = 0.635	RBC = -0.068, CLES = 0.534

**Block 2 Level 3**	**1.90 (0.36)**	**1.85 (0.35)**	**U-test = 643, p-value = 0.414**	**RBC = -0.116, CLES = 0.558**
**Block 2 Level 4**	**1.93 (0.42)**	**1.85 (0.41)**	**U-test = 632.5, p-value = 0.491**	**RBC = -0.098, CLES = 0.549**
**Block 2 Level 5**	**1.94 (0.44)**	**1.84 (0.39)**	**t-test = 0.97, p-value = 0.34**	**d = 0.234, CI d = [-0.249, 0.716]; Cl r = 0.101, Cl r = [-0.135, 0.337]**
**Block 2 Level 6**	**1.95 (0.43)**	**1.95 (0.44)**	**t-test = 0.01, p-value = 0.99**	**d = 0.002, CI d = [-0.479, 0.483]; Cl r = -0.020, Cl r = [-0.255, 0.216]**
Block 3 Level 1	1.97 (0.47)	1.88 (0.36)	t-test = 0.90, p-value = 0.37	d = 0.219, CI d = [-0.263, 0.702]; Cl r = 0.114, Cl r = [-0.122, 0.349]
Block 3 Level 2	1.91 (0.47)	1.85 (0.44)	t-test = 0.59, p-value = 0.56	d = 0.143, CI d = [-0.338, 0.625]; Cl r = 0.058, Cl r = [-0.178, 0.294]
Block 3 Level 3	1.87 (0.41)	1.84 (0.48)	t-test = 0.22, p-value = 0.83	d = 0.053, CI d = [-0.428, 0.534]; Cl r = 0.090, Cl r = [-0.146, 0.326]
Block 3 Level 4	1.82 (0.39)	1.84 (0.43)	U-test = 574, p-value = 1.00	RBC = -0.001, CLES = 0.500
Block 3 Level 5	1.94 (0.46)	1.84 (0.37)	t-test = 1.07, p-value = 0.29	d = 0.260, CI d = [-0.223, 0.743]; Cl r = 0.127, Cl r = [-0.109, 0.363]
Block 3 Level 6	1.85 (0.38)	1.94 (0.49)	t-test = -0.83, p-value = 0.41	d = -0.199, CI d = [-0.686, 0.287]; Cl r = -0.049, Cl r = [-0.287, 0.189]

RSP Phase Duration Inspiration

Table S23. Other ANS metrics, RSP Phase Duration Inspiration by levels.

**Variable**	**Cluster 0 mean (SD)**	**Cluster 1 mean (SD)**	**Statistics**	**Metrics**
Block 1 Level 1	1.48 (0.34)	1.44 (0.29)	U-test = 570.5, p-value = 0.910	RBC = -0.017, CLES = 0.508
Block 1 Level 2	1.52 (0.34)	1.46 (0.32)	U-test = 623, p-value = 0.433	RBC = -0.113, CLES = 0.556
Block 1 Level 3	1.45 (0.26)	1.41 (0.23)	U-test = 582, p-value = 0.474	RBC = -0.104, CLES = 0.552
Block 1 Level 4	1.53 (0.35)	1.39 (0.31)	U-test = 696, p-value = 0.092	RBC = -0.241, CLES = 0.620
Block 1 Level 5	1.57 (0.39)	1.47 (0.38)	U-test = 664.5, p-value = 0.124	RBC = -0.222, CLES = 0.611
Block 1 Level 6	1.53 (0.29)	1.45 (0.26)	U-test = 635, p-value = 0.246	RBC = -0.167, CLES = 0.584
Block 2 Level 1	1.53 (0.33)	1.55 (0.35)	U-test = 540, p-value = 0.979	RBC = 0.005, CLES = 0.498
Block 2 Level 2	1.58 (0.48)	1.56 (0.37)	U-test = 537, p-value = 0.778	RBC = 0.041, CLES = 0.479

Block 2 Level 3	1.61 (0.49)	1.57 (0.52)	U-test = 608.5, p-value = 0.547	RBC = -0.087, CLES = 0.543
Block 2 Level 4	1.55 (0.32)	1.45 (0.29)	U-test = 644, p-value = 0.202	RBC = -0.184, CLES = 0.592
Block 2 Level 5	1.61 (0.43)	1.55 (0.44)	U-test = 659, p-value = 0.361	d = 0.142, CI d = [-0.343, 0.627], Cl r = 0.110, Cl r = [-0.128, 0.348]
Block 2 Level 6	1.60 (0.41)	1.62 (0.55)	U-test = 542, p-value = 0.864	d = -0.039, CI d = [-0.523, 0.446], Cl r = 0.035, Cl r = [-0.203, 0.273]
Block 3 Level 1	1.54 (0.35)	1.60 (0.50)	U-test = 518, p-value = 0.651	d = -0.137, CI d = [-0.626, 0.352], Cl r = 0.002, Cl r = [-0.237, 0.242]
Block 3 Level 2	1.45 (0.31)	1.63 (0.52)	U-test = 490, p-value = 0.120	d = -0.427, CI d = [-0.921, 0.067], Cl r = -0.166, Cl r = [-0.405, 0.074]
Block 3 Level 3	1.51 (0.38)	1.46 (0.34)	U-test = 573, p-value = 0.550	d = 0.147, CI d = [-0.341, 0.636], Cl r = 0.054, Cl r = [-0.186, 0.293]
Block 3 Level 4	1.55 (0.38)	1.48 (0.39)	U-test = 625, p-value = 0.433	RBC = -0.113, CLES = 0.556
Block 3 Level 5	1.56 (0.41)	1.58 (0.49)	U-test = 518, p-value = 0.870	d = -0.041, CI d = [-0.522, 0.440], Cl r = 0.039, Cl r = [-0.197, 0.275]
Block 3 Level 6	1.55 (0.37)	1.58 (0.45)	U-test = 536, p-value = 0.799	d = -0.060, CI d = [-0.544, 0.425], Cl r = 0.022, Cl r = [-0.215, 0.260]

RSP Phase Duration Ratio

Table S24. Other ANS metrics, RSP Phase Duration Ratio by levels.

**Variable**	**Cluster 0 mean (SD)**	**Cluster 1 mean (SD)**	**Statistics**	**Metrics**
Block 1 Level 1	0.79 (0.17)	0.79 (0.14)	U-test = 547, p-value = 0.866	RBC = 0.025, CLES = 0.488
Block 1 Level 2	0.82 (0.17)	0.81 (0.17)	U-test = 595, p-value = 0.665	RBC = -0.063, CLES = 0.531
Block 1 Level 3	0.81 (0.18)	0.79 (0.13)	U-test = 558, p-value = 0.847	RBC = -0.029, CLES = 0.514
Block 1 Level 4	0.83 (0.18)	0.78 (0.19)	U-test = 689, p-value = 0.173	RBC = -0.193, CLES = 0.597
Block 1 Level 5	0.82 (0.17)	0.79 (0.19)	U-test = 631, p-value = 0.376	RBC = -0.127, CLES = 0.563
Block 1 Level 6	0.80 (0.17)	0.76 (0.16)	U-test = 641, p-value = 0.312	RBC = -0.145, CLES = 0.572
Block 2 Level 1	0.86 (0.29)	0.87 (0.24)	U-test = 523, p-value = 0.519	RBC = 0.092, CLES = 0.454
Block 2 Level 2	0.85 (0.25)	0.88 (0.22)	U-test = 509, p-value = 0.414	RBC = 0.116, CLES = 0.442

Block 2 Level 3	0.87 (0.28)	0.84 (0.21)	U-test = 586, p-value = 0.749	RBC = -0.046, CLES = 0.523
Block 2 Level 4	0.84 (0.24)	0.81 (0.20)	U-test = 595, p-value = 0.665	RBC = -0.063, CLES = 0.531
Block 2 Level 5	0.86 (0.28)	0.86 (0.28)	t-test = 0.04, p-value = 0.97	d = 0.009, CI d = [-0.472, 0.490], Cl r = 0.021, CI r [-0.215, 0.257]
Block 2 Level 6	0.85 (0.27)	0.83 (0.23)	t-test = 0.41, p-value = 0.68	d = 0.100, CI d = [-0.385, 0.585], Cl r = 0.077, CI r [-0.161, 0.314]
Block 3 Level 1	0.85 (0.28)	0.88 (0.25)	t-test = -0.41, p-value = 0.68	d = -0.099, CI d = [-0.580, 0.382], Cl r = -0.110, CI r [-0.346, 0.126]
Block 3 Level 2	0.83 (0.29)	0.90 (0.26)	t-test = -1.18, p-value = 0.24	d = -0.286, CI d = [-0.769, 0.197], Cl r = -0.171, CI r [-0.407, 0.065]
Block 3 Level 3	0.84 (0.25)	0.84 (0.25)	t-test = -0.06, p-value = 0.95	d = -0.014, CI d = [-0.499, 0.470], Cl r = -0.044, CI r [-0.282, 0.194]
Block 3 Level 4	0.86 (0.24)	0.83 (0.19)	U-test = 582.5, p-value = 0.782	RBC = -0.040, CLES = 0.520
Block 3 Level 5	0.83 (0.23)	0.86 (0.27)	t-test = -0.50, p-value = 0.62	d = -0.121, CI d = [-0.602, 0.361], Cl r = -0.039, CI r [-0.275, 0.197]
Block 3 Level 6	0.84 (0.20)	0.83 (0.25)	t-test = 0.28, p-value = 0.78	d = 0.068, CI d = [-0.417, 0.552], Cl r = 0.111, CI r [-0.127, 0.349]

RSP Rate Mean

Table S25. Other ANS metrics, RSP Rate Mean by levels.

Block	Cluster 0 mean (SD)	Cluster 1 mean (SD)	Statistics	Metrics
Block 1 Level 1	18.25 (3.05)	18.04 (3.28)	U-test = 577, p-value = 1.000	RBC = 0.001, CLES = 0.500
Block 1 Level 2	18.26 (3.09)	18.29 (3.49)	U-test = 525, p-value = 0.665	RBC = 0.063, CLES = 0.469
Block 1 Level 3	18.52 (3.09)	18.60 (3.28)	U-test = 548, p-value = 0.885	RBC = 0.021, CLES = 0.489
Block 1 Level 4	18.21 (3.11)	18.88 (3.88)	U-test = 524.5, p-value = 0.407	RBC = 0.117, CLES = 0.441
Block 1 Level 5	17.82 (3.09)	18.33 (3.46)	U-test = 487, p-value = 0.363	RBC = 0.130, CLES = 0.435
Block 1 Level 6	17.61 (2.58)	18.04 (2.98)	U-test = 512.5, p-value = 0.555	RBC = 0.085, CLES = 0.458
Block 2 Level 1	17.63 (2.99)	17.79 (3.53)	U-test = 559.5, p-value = 0.700	RBC = 0.055, CLES = 0.473
Block 2 Level 2	17.93 (2.85)	17.88 (3.60)	U-test = 592, p-value = 1.000	RBC = 0.000, CLES = 0.500

Block 2 Level 3	17.70 (2.79)	17.79 (3.99)	U-test = 518, p-value = 0.376	RBC = 0.125, CLES = 0.438
Block 2 Level 4	17.79 (2.70)	18.36 (3.79)	U-test = 493.5, p-value = 0.314	RBC = 0.143, CLES = 0.428
Block 2 Level 5	17.59 (3.13)	18.29 (4.08)	U-test = 504.5, p-value = 0.285	RBC = 0.151, CLES = 0.425
Block 2 Level 6	17.50 (2.98)	17.30 (3.87)	U-test = 579.5, p-value = 0.720	RBC = 0.051, CLES = 0.475
Block 3 Level 1	17.55 (3.27)	17.61 (3.67)	U-test = 583, p-value = 0.751	RBC = 0.045, CLES = 0.477
Block 3 Level 2	18.27 (3.32)	17.79 (4.47)	U-test = 661, p-value = 0.556	RBC = -0.083, CLES = 0.541
Block 3 Level 3	18.48 (3.05)	18.07 (4.51)	t-test = 0.45, p-value = 0.66	d = 0.105, CI d = [-0.373, 0.583], Cl r = -0.037, Cl r = [-0.272, 0.197]
Block 3 Level 4	18.26 (3.32)	18.47 (3.87)	U-test = 563, p-value = 0.732	RBC = 0.049, CLES = 0.476
Block 3 Level 5	17.94 (3.30)	17.94 (3.65)	U-test = 581.5, p-value = 0.737	RBC = 0.048, CLES = 0.476
Block 3 Level 6	18.01 (3.35)	17.37 (3.71)	U-test = 649.5, p-value = 0.651	RBC = -0.064, CLES = 0.532

SCR Peaks Amplitude Mean

Table S26. Other ANS metrics, SCR Peaks Amplitude Mean by levels.

Variable	Cluster 0 mean (SD)	Cluster 1 mean (SD)	Statistics	Metrics
Block 1 Level 1	2.24 (2.70)	2.30 (2.69)	U-test = 529.5, p-value = 0.857	RBC = 0.027, CLES = 0.487
Block 1 Level 2	2.28 (3.55)	2.40 (2.92)	U-test = 456, p-value = 0.354	RBC = 0.135, CLES = 0.433
Block 1 Level 3	2.71 (4.68)	2.88 (4.02)	U-test = 421.5, p-value = 0.122	RBC = 0.223, CLES = 0.388
Block 1 Level 4	3.89 (5.35)	3.37 (5.55)	U-test = 563.5, p-value = 0.970	RBC = -0.006, CLES = 0.503
Block 1 Level 5	4.14 (6.21)	2.91 (4.51)	U-test = 568.5, p-value = 0.920	RBC = -0.015, CLES = 0.508

Block 1 Level 6	2.89 (4.60)	3.40 (5.59)	U-test = 447, p-value = 0.308	RBC = 0.149, CLES = 0.426
Block 2 Level 1	2.73 (3.32)	2.82 (5.09)	U-test = 644.5, p-value = 0.192	RBC = -0.188, CLES = 0.594
Block 2 Level 2	2.22 (3.34)	2.99 (5.62)	U-test = 489.5, p-value = 0.520	RBC = 0.094, CLES = 0.453
Block 2 Level 3	3.76 (5.95)	3.32 (5.51)	U-test = 580, p-value = 0.634	RBC = -0.069, CLES = 0.535
Block 2 Level 4	2.75 (4.71)	3.08 (5.55)	U-test = 521.5, p-value = 0.817	RBC = 0.034, CLES = 0.483
Block 2 Level 5	4.79 (7.32)	3.35 (5.69)	t-test = 0.91, p-value = 0.37	d = 0.221, CI d = [-0.262, 0.703]; Cl r = -0.017, Cl r = [-0.253, 0.219]
Block 2 Level 6	4.71 (6.29)	3.65 (6.25)	t-test = 0.70, p-value = 0.49	d = 0.169, CI d = [-0.313, 0.651]; Cl r = 0.166, Cl r = [-0.070, 0.402]
Block 3 Level 1	2.05 (3.10)	2.97 (5.42)	t-test = -0.87, p-value = 0.39	d = -0.206, CI d = [-0.692, 0.281]; Cl r = -0.025, Cl r = [-0.263, 0.212]
Block 3 Level 2	2.58 (4.79)	3.35 (6.01)	t-test = -0.59, p-value = 0.56	d = -0.140, CI d = [-0.626, 0.347]; Cl r = -0.120, Cl r = [-0.358, 0.117]
Block 3 Level 3	2.83 (5.44)	2.41 (4.80)	t-test = 0.33, p-value = 0.74	d = 0.081, CI d = [-0.407, 0.570]; Cl r = -0.014, Cl r = [-0.253, 0.226]
Block 3 Level 4	2.76 (4.39)	2.45 (4.28)	U-test = 573.5, p-value = 0.850	RBC = -0.028, CLES = 0.514
Block 3 Level 5	4.20 (8.12)	3.08 (6.19)	t-test = 0.64, p-value = 0.53	d = 0.157, CI d = [-0.329, 0.643]; Cl r = -0.023, Cl r = [-0.261, 0.215]
Block 3 Level 6	3.60 (6.04)	2.45 (4.48)	t-test = 0.87, p-value = 0.39	d = 0.216, CI d = [-0.273, 0.706]; Cl r = 0.028, Cl r = [-0.212, 0.267]

SCR Peaks Number

Table S27. Other ANS metrics, SCR Peaks Number by levels.

Variable	Cluster 0 mean (SD)	Cluster 1 mean (SD)	Statistics	Metrics
Block 1 Level 1	16.66 (11.27)	18.50 (12.56)	U-test = 531, p-value = 0.584	RBC = 0.078, CLES = 0.461
Block 1 Level 2	17.80 (15.41)	19.33 (17.17)	U-test = 527, p-value = 0.872	RBC = 0.024, CLES = 0.488
Block 1 Level 3	19.03 (16.33)	21.28 (18.07)	U-test = 535.5, p-value = 0.782	RBC = 0.040, CLES = 0.480
Block 1 Level 4	16.91 (15.81)	22.35 (18.86)	U-test = 456.5, p-value = 0.264	RBC = 0.161, CLES = 0.420
Block 1 Level 5	15.29 (18.02)	20.71 (19.73)	U-test = 394.5, p-value = 0.058	RBC = 0.273, CLES = 0.364
Block 1 Level 6	20.66 (19.55)	22.49 (21.50)	U-test = 562.5, p-value = 0.980	RBC = -0.004, CLES = 0.502
Block 2 Level 1	13.13 (12.93)	15.58 (14.34)	U-test = 507, p-value = 0.525	RBC = 0.091, CLES = 0.454
Block 2 Level 2	18.53 (20.66)	17.57 (17.29)	U-test = 579.5, p-value = 0.885	RBC = 0.021, CLES = 0.489
Block 2 Level 3	16.74 (17.14)	21.46 (21.19)	U-test = 466, p-value = 0.187	RBC = 0.187, CLES = 0.406
Block 2 Level 4	21.75 (23.90)	25.38 (23.79)	U-test = 488, p-value = 0.213	RBC = 0.176, CLES = 0.412
Block 2 Level 5	23.00 (21.29)	28.68 (27.70)	t-test = -0.97, p-value = 0.34	d = -0.228, CI d = [-0.707, 0.251]; Cl r = -0.040, Cl r = [-0.274, 0.194]
Block 2 Level 6	16.70 (19.08)	26.36 (25.66)	t-test = -1.78, p-value = 0.08	d = -0.425, CI d = [-0.911, 0.062]; Cl r = -0.221, Cl r = [-0.457, 0.015]
Block 3 Level 1	11.52 (11.09)	17.05 (13.19)	t-test = -1.88, p-value = 0.06	d = -0.451, CI d = [-0.943, 0.041]; Cl r = -0.281, Cl r = [-0.518, -0.043]
Block 3 Level 2	17.34 (19.98)	18.70 (19.14)	t-test = -0.29, p-value = 0.77	d = -0.070, CI d = [-0.552, 0.412]; Cl r = -0.106, Cl r = [-0.342, 0.129]

Block 3 Level 3	14.48 (14.25)	20.97 (19.68)	t-test = -1.57, p-value = 0.12	d = -0.372, CI d = [-0.863, 0.118]; Cl r = -0.198, Cl r = [-0.436, 0.040]
Block 3 Level 4	16.70 (13.68)	17.72 (16.15)	U-test = 537, p-value = 0.974	RBC = 0.006, CLES = 0.497
Block 3 Level 5	17.34 (16.03)	19.56 (18.99)	t-test = -0.52, p-value = 0.60	d = -0.125, CI d = [-0.611, 0.360]; Cl r = -0.054, Cl r = [-0.291, 0.184]
Block 3 Level 6	18.94 (21.30)	20.49 (21.15)	t-test = -0.30, p-value = 0.76	d = -0.073, CI d = [-0.555, 0.409]; Cl r = -0.086, Cl r = [-0.322, 0.150]

Parameters by blocks

Table S28. Table of HRV parameters consolidated by blocks comparing statistical analysis between Cluster 0 and Cluster 1.

Variable	Cluster 0 mean (SD)	Cluster 1 mean (SD)	Statistics	Metrics
**HRV MeanNN block1**	**826.4 (130.7)**	**763.4 (160.7)**	**U-test = 762, p-value = 0.044**	**RBC = -0.283, CLES = 0.641**
**HRV MeanNN block2**	**855.9 (118.8)**	**785.8 (163.1)**	**U-test = 813, p-value = 0.017**	**RBC = -0.332, CLES = 0.666**
**HRV MeanNN block3**	**842.9 (118.6)**	**769.9 (156.0)**	**U-test = 818, p-value = 0.015**	**RBC = -0.340, CLES = 0.670**
HRV SDNN block1	51.87 (14.76)	46.12 (18.26)	U-test = 712, p-value = 0.096	RBC = -0.236, CLES = 0.618
HRV SDNN block2	52.91 (17.55)	45.66 (15.69)	t-test = 1.80, p-value = 0.077	Cohen's d = 0.438, CI d = [-0.050, 0.925], r = 0.197, CI r = [-0.039, 0.433]
**HRV SDNN block3**	**52.73 (15.71)**	**44.16 (16.26)**	**t-test = 2.22, p-value = 0.030**	**Cohen's d = 0.535, CI d = [0.045, 1.025], r = 0.272, CI r = [0.036, 0.508]**
**HRV RMSSD block1**	**41.38 (15.10)**	**30.45 (15.90)**	**U-test = 822, p-value = 0.003**	**RBC = -0.427, CLES = 0.714**
**HRV RMSSD block2**	**46.16 (24.36)**	**32.17 (18.56)**	**t-test = 2.65, p-value = 0.010**	**Cohen's d = 0.653, CI d = [0.158, 1.147], r = 0.345, CI r = [0.109, 0.581]**
**HRV RMSSD block3**	**44.08 (20.66)**	**31.31 (17.40)**	**t-test = 2.75, p-value = 0.008**	**Cohen's d = 0.673, CI d = [0.178, 1.169], r = 0.335, CI r = [0.099, 0.571]**
HRV LF block1	0.010 (0.007)	0.010 (0.009)	U-test = 641, p-value = 0.576	RBC = -0.079, CLES = 0.540
HRV LF block2	0.012 (0.008)	0.010 (0.008)	t-test = 0.88, p-value = 0.382	Cohen's d = 0.211, CI d = [-0.269, 0.690], r = 0.124, CI r = [-0.110, 0.359]

HRV LF block3	0.014 (0.009)	0.012 (0.009)	t-test = 0.47, p-value = 0.642	Cohen's d = 0.112, CI d = [-0.366, 0.590], r = 0.081, CI r = [-0.153, 0.315]
**HRV HF block1**	**0.010 (0.006)**	**0.006 (0.005)**	**U-test = 811, p-value = 0.001**	**RBC = -0.453, CLES = 0.727**
**HRV HF block2**	**0.012 (0.009)**	**0.007 (0.007)**	**t-test = 2.74, p-value = 0.008**	**Cohen's d = 0.663, CI d = [0.172, 1.154], r = 0.364, CI r = [0.129, 0.598]**
**HRV HF block3**	**0.012 (0.009)**	**0.007 (0.008)**	**t-test = 2.17, p-value = 0.034**	**Cohen's d = 0.531, CI d = [0.038, 1.025], r = 0.335, CI r = [0.098, 0.573]**
**HRV LFHF block1**	**1.16 (0.80)**	**1.96 (1.34)**	**U-test = 372, p-value = 0.012**	**RBC = 0.356, CLES = 0.322**
**HRV LFHF block2**	**1.21 (0.72)**	**2.00 (1.35)**	**t-test = -3.05, p-value = 0.004**	**Cohen's d = -0.718, CI d = [-1.214, -0.221], r = -0.268, CI r = [-0.503, -0.032]**
**HRV LFHF block3**	**1.39 (0.90)**	**2.31 (1.54)**	**t-test = -3.08, p-value = 0.003**	**Cohen's d = -0.717, CI d = [-1.214, -0.220], r = -0.281, CI r = [-0.517, -0.045]**

Table S29. Table of oculomotor parameters consolidated by blocks comparing statistical analysis between Cluster 0 and Cluster 1.

Variable	Cluster 0 mean (SD)	Cluster 1 mean (SD)	Statistics	Metrics
n pupil size max block1	0.52 (0.83)	0.27 (0.94)	t-test = 1.19, p-value = 0.240	Cohen's d = 0.282, CI d = [-0.198, 0.762], r = 0.131, CI r = [-0.103, 0.366]
n pupil size max block2	-0.15 (0.92)	0.16 (0.99)	t-test = -1.37, p-value = 0.174	Cohen's d = -0.327, CI d = [-0.808, 0.154], r = -0.158, CI r = [-0.392, 0.076]
n pupil size max block3	-0.37 (1.06)	-0.43 (0.96)	t-test = 0.25, p-value = 0.800	Cohen's d = 0.061, CI d = [-0.417, 0.539], r = 0.008, CI r = [-0.227, 0.242]
n pupil size mean block1	0.82 (0.75)	0.65 (0.86)	t-test = 0.89, p-value = 0.378	Cohen's d = 0.211, CI d = [-0.269, 0.690], r = 0.123, CI r = [-0.111, 0.357]
**n pupil size mean block2**	**-0.51 (0.74)**	**0.15 (0.82)**	**t-test = -3.50, p-value = 0.001**	**Cohen's d = -0.833, CI d = [-1.331, -0.335], r = -0.387, CI r = [-0.622, -0.153]**
**n pupil size mean block3**	**-0.32 (0.96)**	**-0.80 (0.74)**	**t-test = 2.33, p-value = 0.023**	**Cohen's d = 0.567, CI d = [0.079, 1.054], r = 0.295, CI r = [0.060, 0.529]**
n pupil size min block1	0.30 (0.99)	0.43 (0.93)	t-test = -0.54, p-value = 0.591	Cohen's d = -0.131, CI d = [-0.613, 0.351], r = -0.048, CI r = [-0.284, 0.188]
n pupil size min block2	-0.11 (0.89)	-0.04 (0.92)	t-test = -0.31, p-value = 0.758	Cohen's d = -0.074, CI d = [-0.552, 0.404], r = -0.055, CI r = [-0.289, 0.179]
n pupil size min block3	-0.23 (1.06)	-0.39 (1.01)	t-test = 0.64, p-value = 0.524	Cohen's d = 0.154, CI d = [-0.325, 0.632], r = 0.089, CI r = [-0.145, 0.324]
**saccade count block1**	**679.5 (230.8)**	**874.1 (220.2)**	**U-test = 313, p-value = 0.000**	**RBC = 0.487, CLES = 0.256**
**saccade count block2**	**666.2 (188.9)**	**868.4 (190.8)**	**t-test = -4.45, p-value = 0.000**	**Cohen's d = -1.065, CI d = [-1.575, -0.554], r = -0.481, CI r = [-0.715, -0.247]**

**saccade count block3**	**656.6 (198.2)**	**822.2 (166.5)**	**U-test = 293.5, p-value = 0.000**	**RBC = 0.519, CLES = 0.240**
saccade velocity mean block1	94.5 (19.0)	100.3 (23.6)	U-test = 503.5, p-value = 0.210	RBC = 0.175, CLES = 0.412
saccade velocity mean block2	94.3 (20.4)	102.4 (23.1)	t-test = -1.55, p-value = 0.125	Cohen's d = -0.369, CI d = [-0.851, 0.112], r = -0.191, CI r = [-0.425, 0.044]
saccade velocity mean block3	93.6 (20.0)	100.6 (20.1)	t-test = -1.46, p-value = 0.148	Cohen's d = -0.350, CI d = [-0.831, 0.131], r = -0.216, CI r = [-0.450, 0.018]
saccade duration mean block1	72.4 (27.1)	74.2 (26.2)	U-test = 587, p-value = 0.787	RBC = 0.038, CLES = 0.481
saccade duration mean block2	77.3 (34.3)	80.6 (25.7)	t-test = -0.45, p-value = 0.651	Cohen's d = -0.111, CI d = [-0.589, 0.368], r = -0.105, CI r = [-0.339, 0.129]
saccade duration mean block3	77.1 (30.6)	81.8 (27.7)	U-test = 535, p-value = 0.378	RBC = 0.124, CLES = 0.438
**saccade duration sum block1**	**49615.9 (25360.9)**	**61813.7 (23560)**	**t-test = -2.06, p-value = 0.043**	**Cohen's d = -0.499, CI d = [-0.988, -0.011], r = -0.252, CI r = [-0.488, -0.016]**
**saccade duration sum block2**	**50753.7 (24103.7)**	**70258.5 (26342.1)**	**t-test = -3.23, p-value = 0.002**	**Cohen's d = -0.771, CI d = [-1.266, -0.275], r = -0.366, CI r = [-0.601, -0.132]**
**saccade duration sum block3**	**49955.1 (23157.3)**	**66874.3 (23664.5)**	**t-test = -3.02, p-value = 0.004**	**Cohen's d = -0.722, CI d = [-1.215, -0.229], r = -0.337, CI r = [-0.571, -0.103]**
saccade amplitude mean block1	1.72 (0.44)	1.87 (0.47)	t-test = -1.43, p-value = 0.156	Cohen's d = -0.344, CI d = [-0.830, 0.141], r = -0.164, CI r = [-0.400, 0.072]
saccade amplitude mean block2	1.76 (0.40)	1.91 (0.45)	t-test = -1.46, p-value = 0.150	Cohen's d = -0.351, CI d = [-0.840, 0.137], r = -0.163, CI r = [-0.401, 0.075]
saccade amplitude mean block3	1.66 (0.38)	1.85 (0.41)	t-test = -1.96, p-value = 0.055	Cohen's d = -0.474, CI d = [-0.965, 0.018], r = -0.243, CI r = [-0.481, -0.005]
**fixation duration mean block1**	**446.9 (133.1)**	**354.9 (84.5)**	**t-test = 3.37, p-value = 0.001**	**Cohen's d = 0.839, CI d = [0.337, 1.342], r = 0.327, CI r = [0.091, 0.563]**
**fixation duration mean block2**	**450.5 (116.1)**	**348.4 (73.0)**	**t-test = 4.30, p-value = 0.000**	**Cohen's d = 1.071, CI d = [0.556, 1.586], r = 0.413, CI r = [0.177, 0.649]**
**fixation duration mean block3**	**441.2 (108.6)**	**353.7 (64.7)**	**t-test = 3.99, p-value = 0.000**	**Cohen's d = 0.997, CI d = [0.486, 1.507], r = 0.401, CI r = [0.165, 0.637]**
**fixation count block1**	**754.6 (222.8)**	**947.0 (207.1)**	**U-test = 311, p-value = 0.000**	**RBC = 0.491, CLES = 0.255**
**fixation count block2**	**736.7 (186.6)**	**937.2 (182.1)**	**U-test = 258, p-value = 0.000**	**RBC = 0.577, CLES = 0.211**
**fixation count block3**	**732.3 (193.2)**	**893.2 (159.8)**	**U-test = 292, p-value = 0.000**	**RBC = 0.522, CLES = 0.239**
**blink count block1**	**679.48 (230.80)**	**874.14 (220.15)**	**U test = 313, p-value = 0.000**	**RBC = 0.487, CLES = 0.256**
**blink count block2**	**666.21 (188.88)**	**868.41 (190.82)**	**t-test = -4.45, p-value = 0.000**	**Cohen's d = -1.065, CI d = [-1.575, -0.554], r = -0.481, CI r = [-0.715, -0.247]**
**blink count block3**	**656.61 (198.21)**	**822.22 (166.51)**	**U-test = 293.5, p-value = 0.000**	**RBC = 0.519, CLES = 0.240**

blink duration block1	28541.52 (19245.79)	32715.61 (16710.54)	t-test = -0.96, p-value = 0.342	Cohen's d = -0.232, CI d = [-0.715, 0.250], r = -0.143, CI r = [-0.379, 0.093]
blink duration block2	30876.00 (20122.05)	38546.50 (17593.79)	t-test = -1.68, p-value = 0.098	Cohen's d = -0.407, CI d = [-0.893, 0.079], r = -0.215, CI r = [-0.451, 0.020]
blink duration block3	30029.88 (18636.61)	38861.14 (18270.99)	t-test = -2.00, p-value = 0.050	Cohen's d = -0.479, CI d = [-0.963, 0.006], r = -0.241, CI r = [-0.475, -0.007]

Table S30. Table of cognitive parameters consolidated by blocks comparing statistical analysis between Cluster 0 and Cluster 1.

Variable	Cluster 0 mean (SD)	Cluster 1 mean (SD)	Statistics	Metrics
Self-estimation Sum block1	20.88 (3.98)	22.00 (3.32)	U-test = 514, p-value = 0.257	RBC = 0.158, CLES = 0.421
Self-estimation Sum block2	18.42 (3.71)	20.49 (3.15)	U-test = 444.5, p-value = 0.050	RBC = 0.272, CLES = 0.364
Self-estimation Sum block3	24.52 (2.56)	24.08 (2.82)	t-test = 0.67, p-value = 0.502	Cohen's d = 0.161, CI d = [-0.318, 0.639], r = 0.048, CI r = [-0.186, 0.282]
**Sum Time block1**	**368928.2 (27697.3)**	**387987.0 (24406.8)**	**U-test = 365, p-value = 0.004**	**RBC = 0.402, CLES = 0.299**
**Sum Time block2**	**368884.2 (22630.5)**	**384581.2 (21137.0)**	**U-test = 354, p-value = 0.003**	**RBC = 0.420, CLES = 0.290**
**Sum Time block3**	**360206.1 (21262.4)**	**375877.1 (22418.5)**	**U-test = 355, p-value = 0.004**	**RBC = 0.400, CLES = 0.300**
mistakes block1	13.58 (5.88)	11.89 (4.64)	U-test = 671, p-value = 0.231	RBC = -0.170, CLES = 0.585
mistakes block2	13.42 (4.52)	12.46 (4.76)	U-test = 629.5, p-value = 0.492	RBC = -0.098, CLES = 0.549
mistakes block3	9.52 (4.40)	9.11 (3.13)	U-test = 586, p-value = 0.882	RBC = -0.022, CLES = 0.511

Table S31. Table of respiratory and skin-galvanic reactions parameters consolidated by blocks comparing statistical analysis between Cluster 0 and Cluster 1.

Variable	Cluster 0 mean (SD)	Cluster 1 mean (SD)	Statistics	Metrics
RSP Amplitude Mean block1	18.68 (9.23)	19.53 (7.07)	U-test = 511, p-value = 0.543	RBC = 0.088, CLES = 0.456
RSP Amplitude Mean block2	18.01 (9.73)	20.08 (9.43)	t-test = -0.89, p-value = 0.375	Cohen's d = -0.216, CI d = [-0.699, 0.267], r = -0.116, CI r = [-0.352, 0.120]

RSP Amplitude Mean block3	17.85 (10.02)	18.96 (6.96)	t-test = -0.52, p-value = 0.604	Cohen's d = -0.130, CI d = [-0.615, 0.356], r = -0.121, CI r = [-0.358, 0.117]
RSP Phase Duration Expiration block1	1.90 (0.36)	1.85 (0.32)	U-test = 623.5, p-value = 0.577	RBC = -0.080, CLES = 0.540
RSP Phase Duration Expiration block2	1.93 (0.36)	1.86 (0.34)	t-test = 0.79, p-value = 0.433	Cohen's d = 0.190, CI d = [-0.292, 0.673], r = 0.087, CI r = [-0.148, 0.323]
RSP Phase Duration Expiration block3	1.86 (0.32)	1.86 (0.38)	t-test = -0.06, p-value = 0.955	Cohen's d = -0.014, CI d = [-0.499, 0.471], r = 0.037, CI r = [-0.200, 0.275]
RSP Phase Duration Inspiration block1	1.53 (0.31)	1.48 (0.35)	U-test = 651, p-value = 0.370	RBC = -0.127, CLES = 0.564
RSP Phase Duration Inspiration block2	1.60 (0.39)	1.52 (0.31)	t-test = 0.91, p-value = 0.364	Cohen's d = 0.224, CI d = [-0.265, 0.714], r = 0.086, CI r = [-0.154, 0.325]
RSP Phase Duration Inspiration block3	1.56 (0.38)	1.56 (0.40)	t-test = 0.02, p-value = 0.981	Cohen's d = 0.006, CI d = [-0.479, 0.490], r = 0.028, CI r = [-0.210, 0.265]
RSP Phase Duration Ratio block1	0.82 (0.16)	0.79 (0.14)	U-test = 648.5, p-value = 0.387	RBC = -0.123, CLES = 0.561
RSP Phase Duration Ratio block2	0.85 (0.24)	0.84 (0.16)	t-test = 0.31, p-value = 0.757	Cohen's d = 0.076, CI d = [-0.408, 0.561], r = 0.002, CI r = [-0.235, 0.240]
RSP Phase Duration Ratio block3	0.84 (0.22)	0.84 (0.20)	t-test = 0.16, p-value = 0.877	Cohen's d = 0.038, CI d = [-0.447, 0.522], r = 0.035, CI r = [-0.203, 0.273]
RSP Rate Mean block1	18.01 (2.81)	18.30 (3.49)	U-test = 555.5, p-value = 0.648	RBC = 0.065, CLES = 0.468
RSP Rate Mean block2	17.52 (2.76)	17.69 (3.76)	U-test = 547.5, p-value = 0.462	RBC = 0.103, CLES = 0.448
RSP Rate Mean block3	17.96 (3.06)	17.75 (3.72)	U-test = 594.5, p-value = 0.855	RBC = 0.026, CLES = 0.487
SCR Peaks Amplitude Mean block1	5.21 (6.78)	3.54 (4.92)	U-test = 582, p-value = 0.787	RBC = -0.039, CLES = 0.520
SCR Peaks Amplitude Mean block2	5.60 (7.68)	5.22 (8.29)	t-test = 0.20, p-value = 0.843	Cohen's d = 0.048, CI d = [-0.434, 0.530], r = 0.071, CI r = [-0.165, 0.307]
SCR Peaks Amplitude Mean block3	4.44 (6.55)	3.34 (5.79)	t-test = 0.73, p-value = 0.470	Cohen's d = 0.178, CI d = [-0.308, 0.664], r = 0.141, CI r = [-0.097, 0.378]
SCR Peaks N block1	62.63 (53.91)	92.72 (83.44)	U-test = 444, p-value = 0.106	RBC = 0.229, CLES = 0.385
SCR Peaks N block2	60.34 (73.10)	82.81 (86.99)	t-test = -1.17, p-value = 0.248	Cohen's d = -0.278, CI d = [-0.762, 0.206], r = -0.156, CI r = [-0.392, 0.080]
SCR Peaks N block3	44.81 (46.77)	64.94 (57.61)	t-test = -1.59, p-value = 0.117	Cohen's d = -0.381, CI d = [-0.871, 0.108], r = -0.267, CI r = [-0.504, -0.029]

Figures by blocks

Figure S1. HRV time domains by blocks with increasing cognitive load with false feedback in block 2 levels 3-6. (A) MeanNN, (B) SDNN, (C) RMSSD parameters.

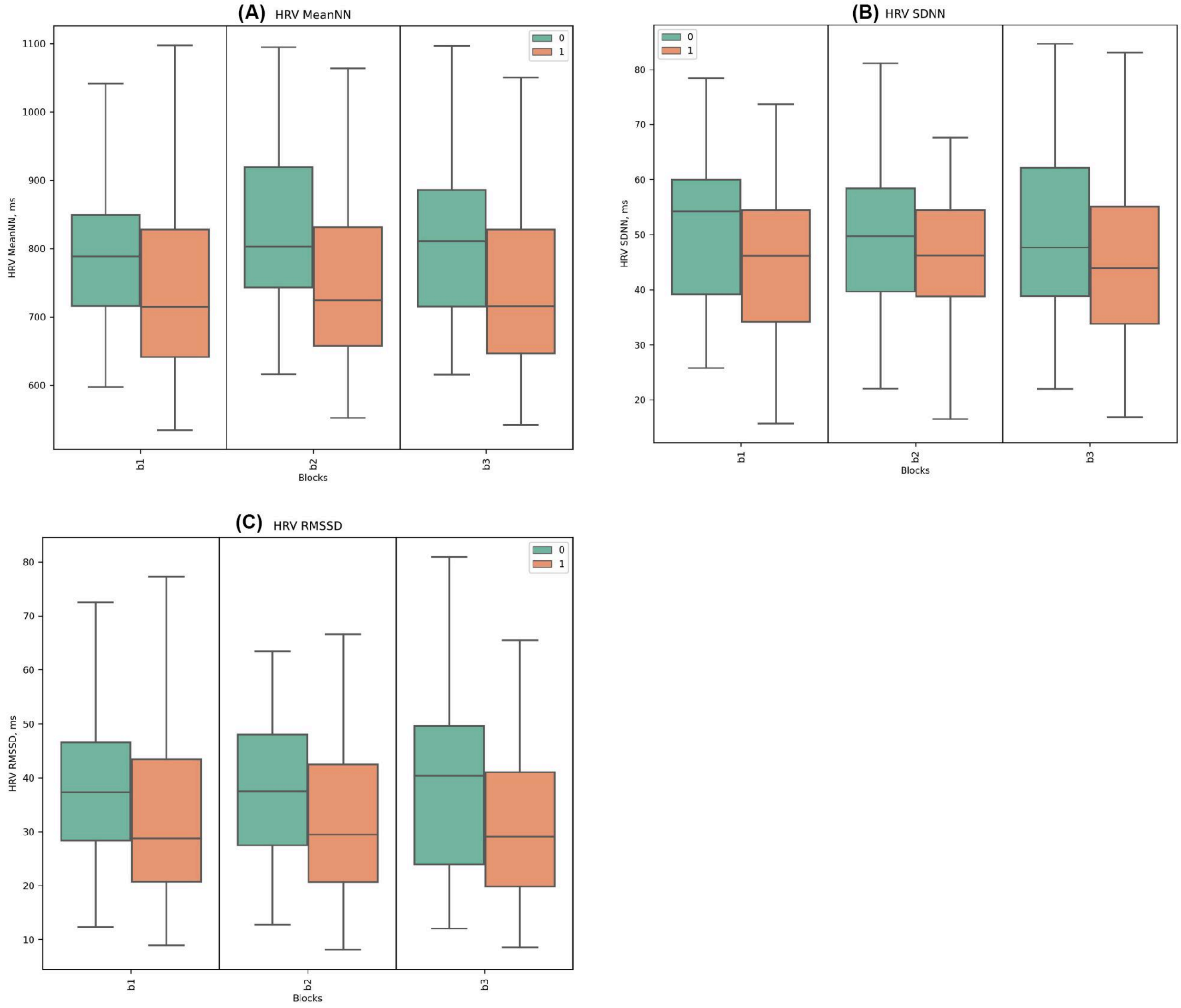

Figure S2. HRV frequency domains by blocks with increasing cognitive load with false feedback in block 2 levels 3-6. (A) HRV HF, (B) HRV LF, (C) HRV LF/HF ratio.

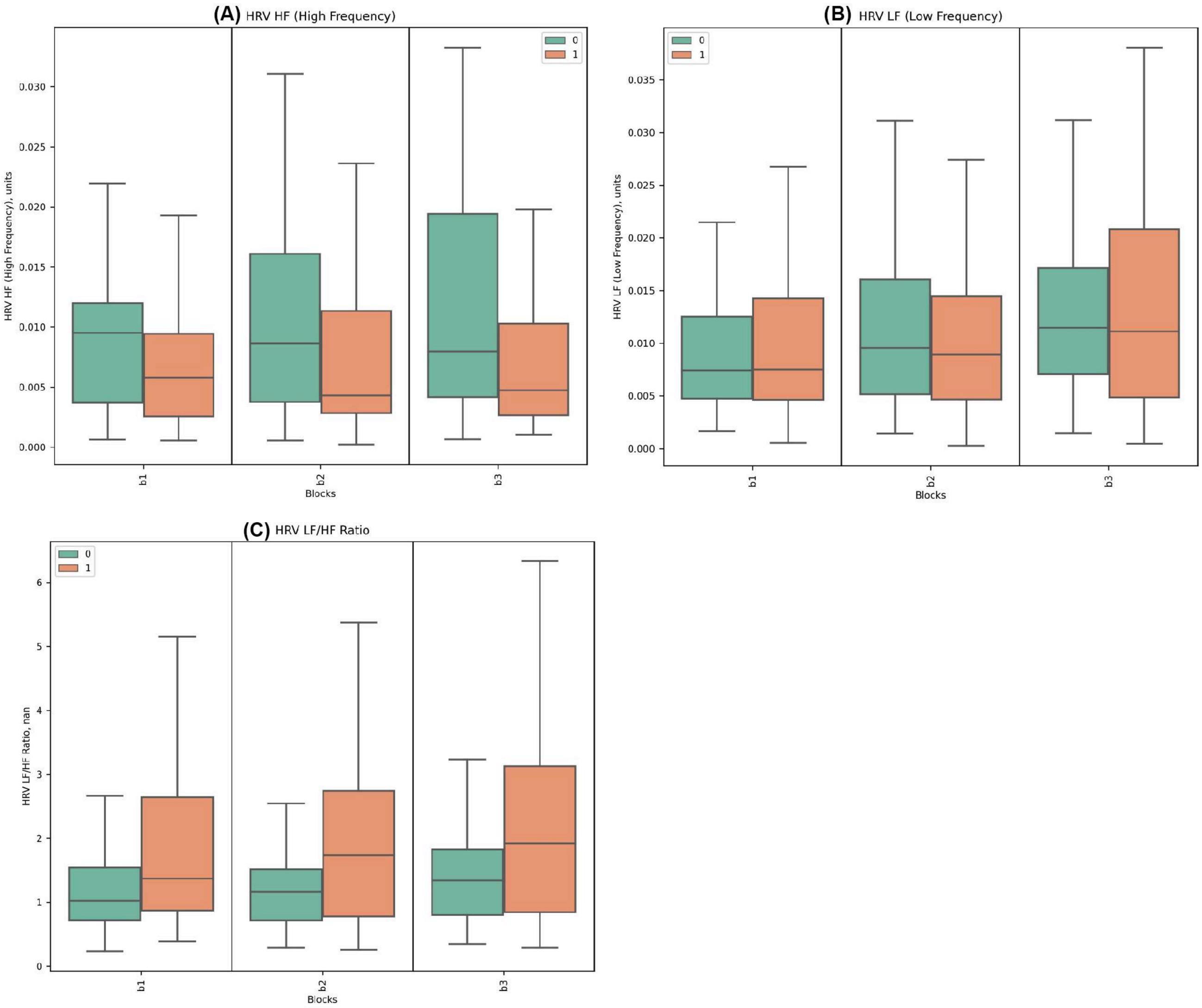

Figure S3. Pupillometry metrics by blocks with increasing cognitive load with false feedback in block 2 levels 3-6. (A) Normalized pupil size max, (B) Normalized pupil size mean, (C) Normalized pupil size min.

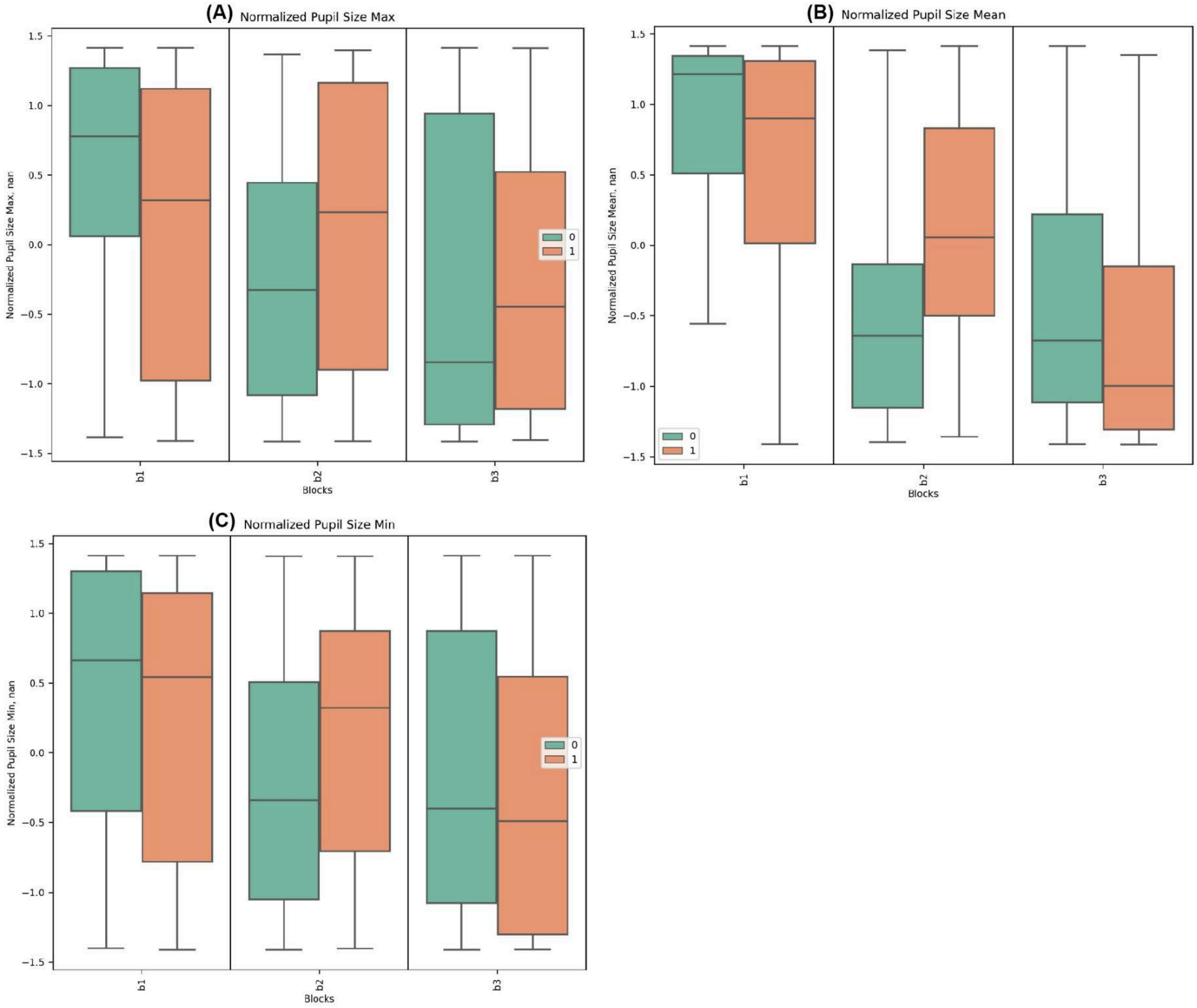

Figure S4. Saccade metrics by blocks with increasing cognitive load with false feedback in block 2 levels 3-6. (A) Saccade count, (B) Saccade velocity mean, (C) Saccade duration sum, (D) Saccade amplitude mean.

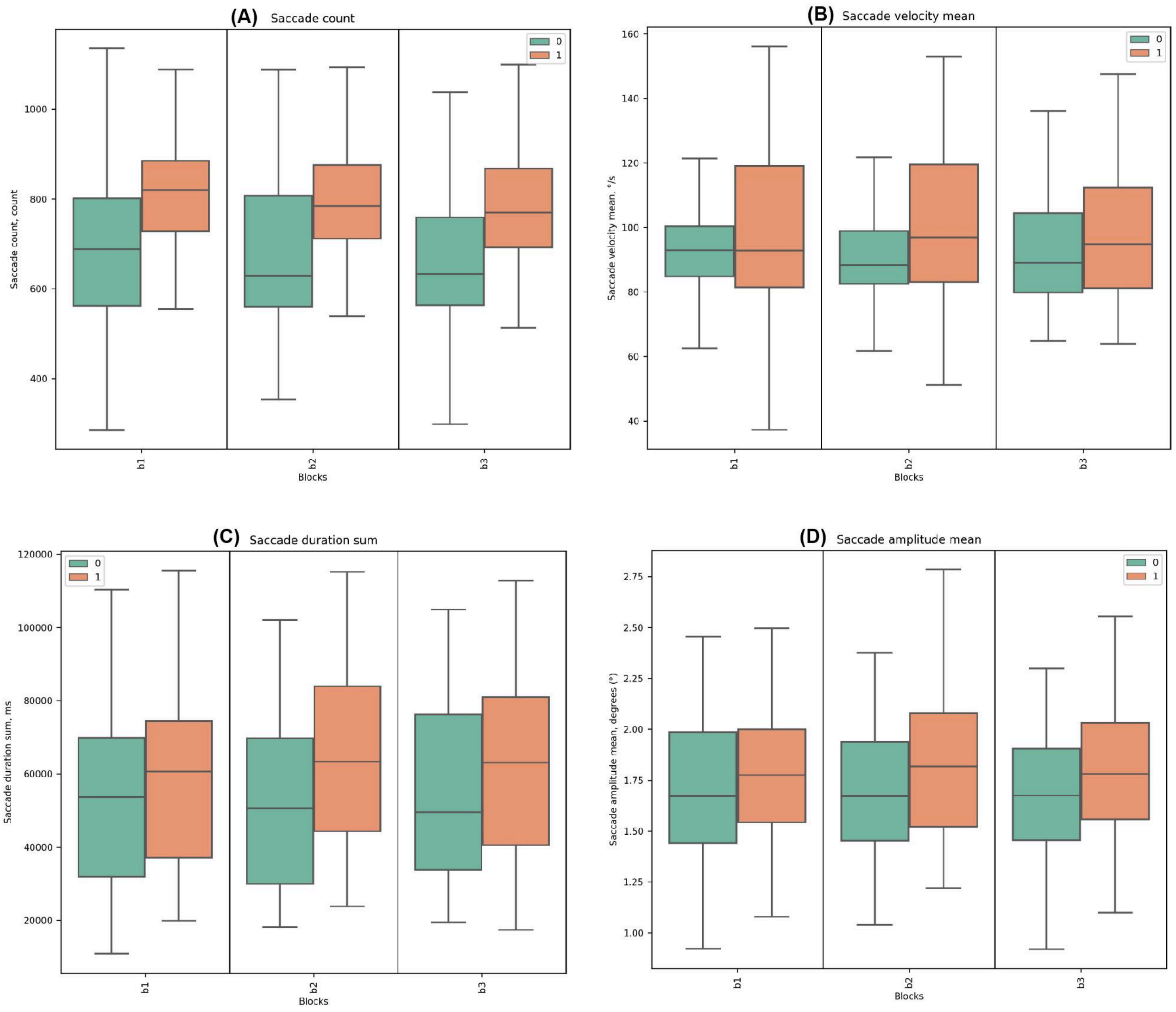

Figure S5. Fixations and blinks by blocks with increasing cognitive load with false feedback in block 2 levels 3-6. (A) Fixation duration mean, (B) Fixation count, (C) Blink count, (D) Blink duration.

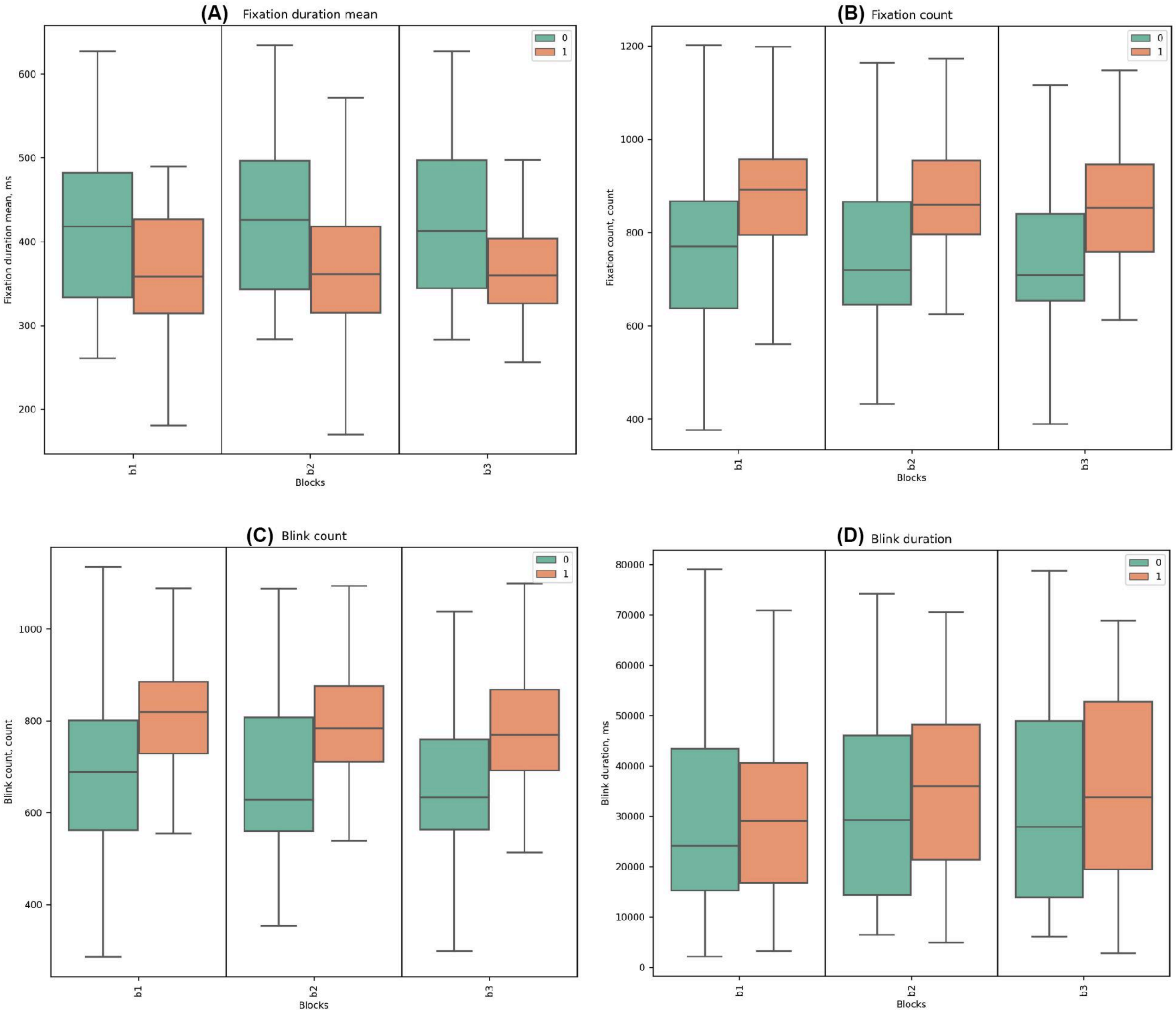

Figure S6. Cognitive metrics by blocks with increasing cognitive load with false feedback in block 2 levels 3-6. (A) Mistakes, (B) Time sum, (C) Self-estimation sum.

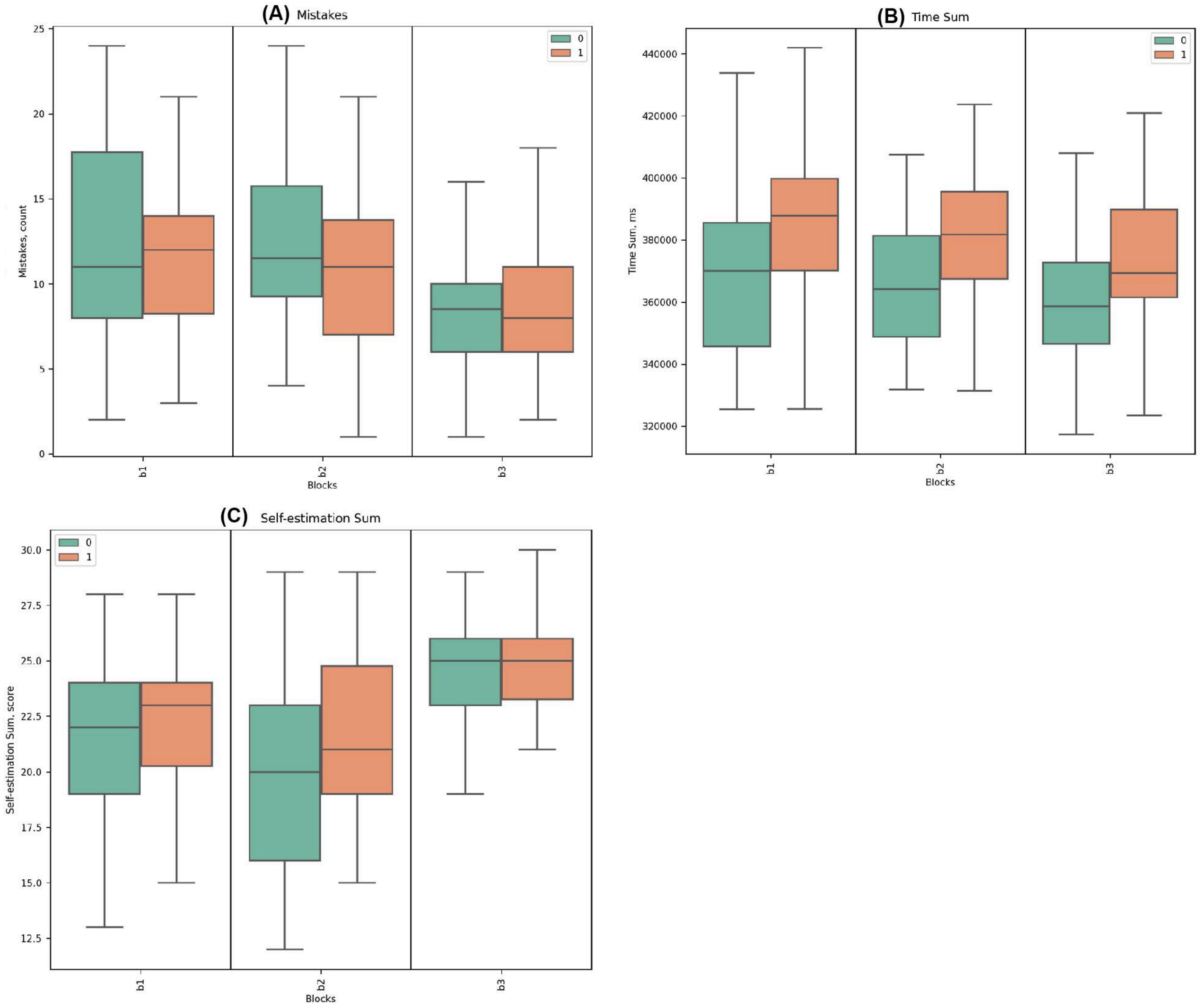

Figure S7. Respiratory metrics by blocks with increasing cognitive load with false feedback in block 2 levels 3-6. (A) RSP amplitude mean, (B) RSP phase duration (expiration), (C) RSP phase duration (inspiration), (D) RSP phase duration ratio (exp/insp), (F) RSP rate mean.

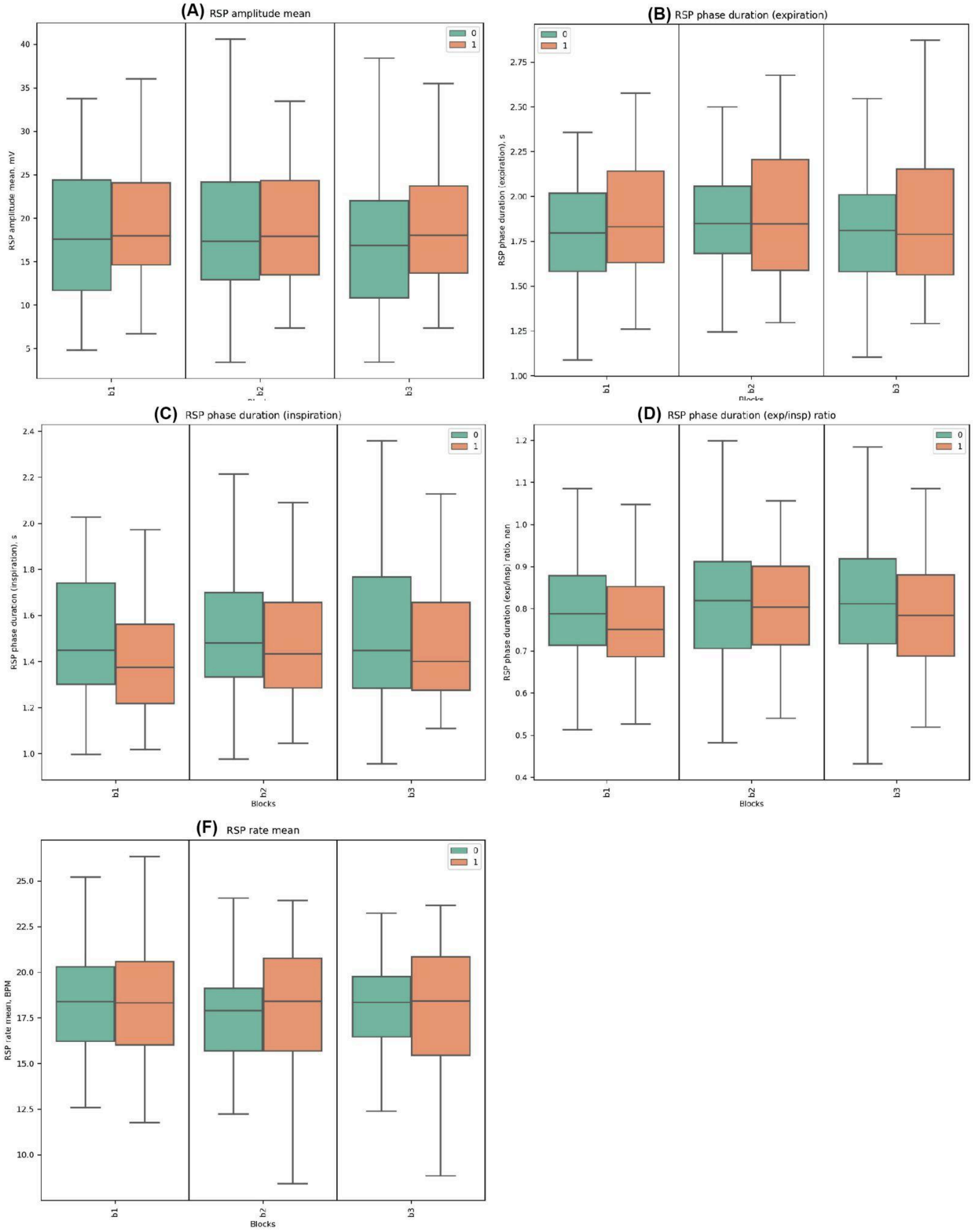

Figure S8. Skin-galvanic reactions metrics by blocks with increasing cognitive load with false feedback in block 2 levels 3-6. (A) SCR peaks amplitude mean, (B) SCR peaks count.

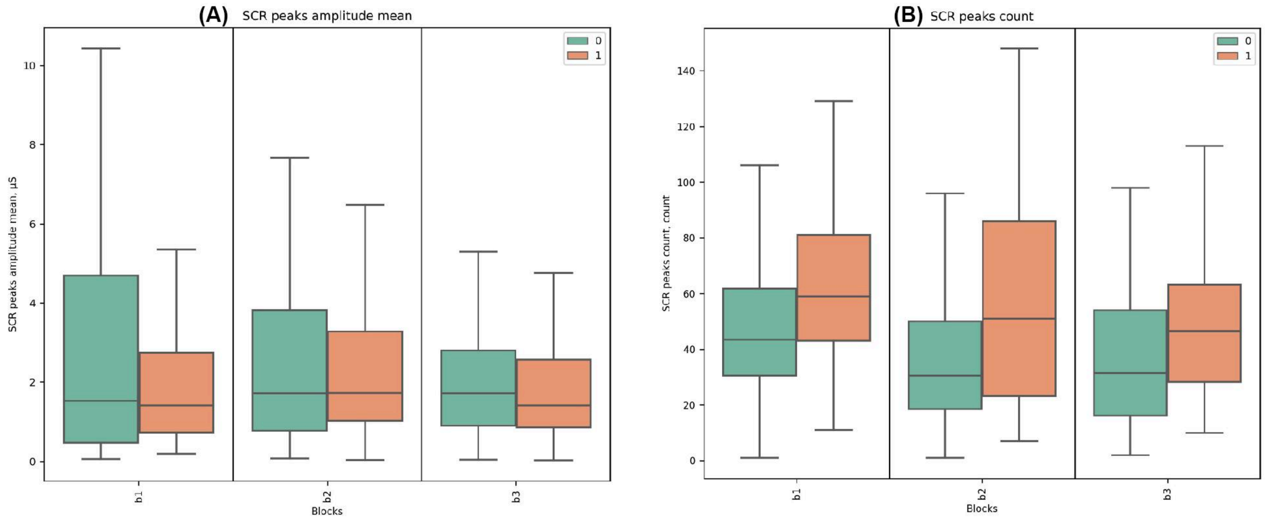

Supplement: Supplementary file 1 [file Data_Sheet_1.pdf]
